# N-Terminal selective modification of peptides and proteins using 2-ethynylbenzaldehydes

**DOI:** 10.1038/s42004-020-0309-y

**Published:** 2020-05-29

**Authors:** Jie-Ren Deng, Nathanael Chun-Him Lai, Karen Ka-Yan Kung, Bin Yang, Sai-Fung Chung, Alan Siu-Lun Leung, Man-Chung Choi, Yun-Chung Leung, Man-Kin Wong

**Affiliations:** 1 State Key Laboratory of Chemical Biology and Drug Discovery, and Department of Applied Biology and Chemical Technology, The Hong Kong Polytechnic University, Hung Hum, Hong Kong China; 2 Henry Cheng Research Laboratory for Drug Development and Lo Ka Chung Centre for Natural Anti-Cancer Drug Development, The Hong Kong Polytechnic University, Hung Hum, Hong Kong China

**Keywords:** Synthetic chemistry methodology, Chemical modification

## Abstract

Selective modification of the N-terminus of peptides and proteins is a promising strategy for single site modification methods. Here we report N-terminal selective modification of peptides and proteins by using 2-ethynylbenzaldehydes (2-EBA) for the production of well-defined bioconjugates. After reaction screening with a series of 2-EBA, excellent N-terminal selectivity is achieved by the reaction in slightly acidic phosphate-buffered saline using 2-EBA with electron-donating substituents. Selective modification of a library of peptides XSKFR (X = either one of 20 natural amino acids) by 2-ethynyl-4-hydroxy-5-methoxybenzaldehyde (**2d**) results in good-to-excellent N-terminal selectivity in peptides (up to >99:1). Lysozyme, ribonuclease A and a therapeutic recombinant *Bacillus caldovelox* arginase mutant (BCArg mutant) are N-terminally modified using alkyne- and fluorescein-linked 2-EBA. Alkyne-linked BCArg mutant is further modified by rhodamine azide via copper(I)-catalyzed [3 + 2] cycloaddition indicating that the reaction has high functional group compatibility. Moreover, the BCArg mutant modified by 2-ethynyl-5-methoxybenzaldehyde (**2b**) exhibits comparable activity in enzymatic and cytotoxic assays with the unmodified one.

## Abstract (web summary)

Selective functionalisation of the N-terminus of peptides and proteins may offer a useful tool for single-site modification. Here a general method for selective N-terminal labeling of peptides is reported, based on cyclisation of ethynylbenzaldehyde to form stable isoquinolinium salts.

## Introduction

Site-selective chemical modification of peptides and proteins has become an emerging research field in chemical biology, which allows the production of well-defined bioconjugates for biological studies and drug development^1–4^. Although a number of bioconjugation reactions for specific amino acid modification have been developed in the past decade, due to the prevalence of multiple targeted residues on protein surface, only a few of them are amenable to give single-site modification^5,6^. To achieve site-selective modification, current methods mainly focus on labeling of the low abundant free cysteine residue or non-canonical amino acids, which always require sophisticated sequence engineering^7–12^. Besides, a few examples targeting the C-terminus or specific lysine *ε*-amino group have also been reported^13–16^. Despite these advances, it is still of ongoing interest to develop new methods for site-selective protein functionalization.

Targeting the N-terminus of peptides and proteins is a promising strategy to achieve single-site modification as a single-chain protein contains only one N-terminal residue in its sequence and it is mostly solvent exposed for functionalization^17,18^. On the other hand, recent studies suggest that a change of the charge in the N-terminal region of the signal peptide would disrupt the translocation of the small secretory preproteins^19^. Thus, development of an efficient N-terminal modification method will not only be an important direction for site-selective protein bioconjugation but also provide a chemical biology approach to study the biological functions of the protein N-terminus.

To achieve site-selective modification on the N-terminus, the important strategy is to perform the modification using pH control. As the N-terminal *α*-amino group possesses a lower basicity (pKa ≈ 6–8) compared with the lysine *ε*-amino group (pKa ≈ 10), a reaction medium with well-controlled pH value could favor the modification on the N-terminal *α*-amino group^20^. Based on this mechanism, N-terminal azidation, acylation, oxidation, and reductive alkylation have been reported by our group and others^21–25^. Another strategy is to utilize the specific residues on the N-terminus. Following this strategy, pyridoxal-5-phosphate (PLP) or Rapoport’s salt (RS)-mediated transamination reaction involving tautomerization triggered by the lower pKa *α*-proton on the N-terminal amino acid^26–28^, as well as 2-pyridinecarboxyaldehyde (2-PCA)-mediated imidazolidinone formation via cyclization of the imine intermediate with the nearby amide group on the N-terminus^29^, have been reported by Francis and co-workers. Among those reactions, the one-step modifications using ketenes^22,23^ or 2-PCA^29^ without addition of oxidizing or reducing reagent are promising approaches to achieve regioselective modified bioconjugates. However, preparation of their derivatives requires multi-step synthesis and it hampered the further studies on their structure-reactivity relationship, stability of the conjugates, as well as their applications. Thus, it is still of importance to investigate new, efficient, and convenient approaches for site-specific labeling of the N-terminus.

**Fig. 1:**
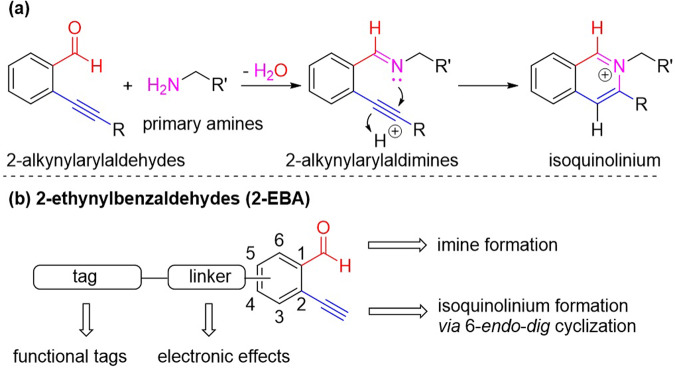
N-terminus selective modification. **a** General mechanism of isoquinolinium formation. **b** Our design of 2-ethynylbenzaldehydes for N-terminal modification.

2-Alkynylarylaldehydes are versatile building blocks in organic synthesis^30–33^. Under transition metal catalysis, the in situ generated 2-alkynylarylaldimines between 2-alkynylarylaldehydes and primary amines could undergo 6-*endo*-*dig* cyclizations to give the corresponding isoquinoliniums, which have been demonstrated as key intermediates for syntheses of complex heterocycles^31,32^. Despite the fact that this transformation has been extensively explored in organic synthesis, studies on its applicability on protein modification remain largely elusive. We hypothesize that the efficient imine formation and tandem cyclization would render 2-alkynylarylaldehydes amenable to selectively modify the N-terminal *α*-amino group (Fig. 1).

In this work, we first report a metal-free one-step N-terminal modification of peptides and proteins using 2-ethynylbenzaldehydes (2-EBA) under mild reaction conditions. The isoquinoliniums formed have been isolated and characterized by a model reaction. After a comprehensive study on the reaction conditions and the structure-reactivity relationship of the reagent, we demonstrate that, apart from the pH control, electronic effects also play important roles in controlling the N-terminal selectivity of the modification. We have also extended this reaction to protein modification, including labeling a therapeutic *Bacillus caldovelox* arginase mutant (BCArg mutant). The enzymatic and anti-cancer activities of the modified BCArg mutant have also been studied.

## Results and discussion

### 2-Ethynylbenzaldehydes as N-terminal selective reagents

**Fig. 2:**
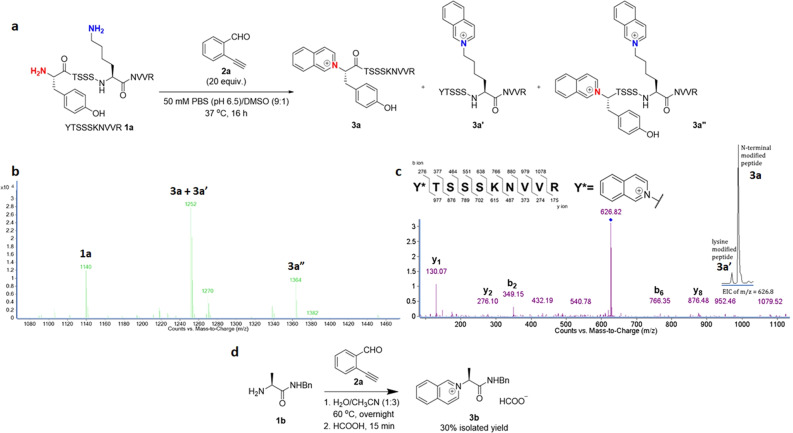
N-terminal modification of a model peptide. **a** N-terminal modification of YTSSSKNVVR **1a** with 2-ethynylbenzaldehyde **2a**. **b** Deconvoluted mass spectrum of the reaction mixture. **c** MS/MS spectrum of N-terminally modified peptide **3a** (inset: extracted ion chromatogram of mono-modified peptide). **d** Model reaction of isoquinolinium salt **3b** from l-alanine *β*-benzylamide **1b** and **2a**.

To begin our study, peptide YTSSSKNVVR **1a** (molecular mass of 1140 Da, 0.1 mM) was treated with 20 equivalents of 2-ethynylbenzaldehyde **2a** (2-EBA, 130 Da) in 50 mM phosphate-buffered saline (PBS)/DMSO (9:1) at pH 6.5 for 16 h (Fig. 2a). After the reaction, we found that peptide **1a** was modified to give mono-modified peptides (N-terminally modified peptide **3a** and lysine-modified peptide **3a′**) in 64% conversion and di-modified peptide **3a″** in 8% conversion (Fig. 2b). An increase of the molecular mass by 112 Da indicated that 2-EBA **2a** was incorporated on the peptide **1a** with loss of a H_2_O molecule, which was presumably ascribed to the formation of a quinolinium conjugate after the modification. As N-terminal selectivity^23–25^ is calculated based on the ratio of the mono-modified peptide at N-terminal *α*-amino group to lysine *ε*-amino group as determined by extracted ion chromatogram (EIC) of LC-MS analysis, we achieved N-terminal selectivity (**3a**:**3a′**) of 96:4 in the mono-modified peptides, with the corresponding MS/MS spectrum of N-terminally modified peptide **3a** as the major product (Fig. 2c). Therefore, the conversions of N-terminally modified peptide **3a** and lysine-modified peptide **3a′** were calculated as 61 and 3% respectively. To give an understanding on the proportion of N-terminally modified peptide in overall modified products, we included another method for determining the efficiency of N-terminal modification, referring to the ratio of the conversion of N-terminally modified peptide over the conversion of all modified peptides (i.e. **3a**/(**3a** + **3a′**+**3aʺ**)). Thus, the efficiency of N-terminal modification of YTSSSKNVVR **1a** with 2-EBA **2a** was 0.85.

To investigate the structure of N-terminally modified peptide **3a**, we conducted a model study by treatment of L-alanine *β*-benzylamide **1b** with 2-EBA **2a** (1.1 equiv.) in H_2_O/CH_3_CN (1:3) at 60 °C overnight, followed by addition of formic acid (2 equivalents) for 15 min (Fig. 2d). After the reaction, the corresponding isoquinolinium **3b** was isolated in 30% yield. The formation of **3b** suggested that the imine was first generated by reaction of **1b** and **2a** to give 2-ethynylbenzaldimine as a key intermediate. Then, the 2-ethynylbenzaldimine intermediate underwent subsequent intramolecular 6-*endo*-*dig* cyclization to give isoquinolinium **3b** as the product. Remarkably, the formation of isoquinolinium salts by reaction of 2-EBA and primary amines under a metal-free condition in aqueous media has not yet been reported previously^30–33^.

**Fig. 3:**
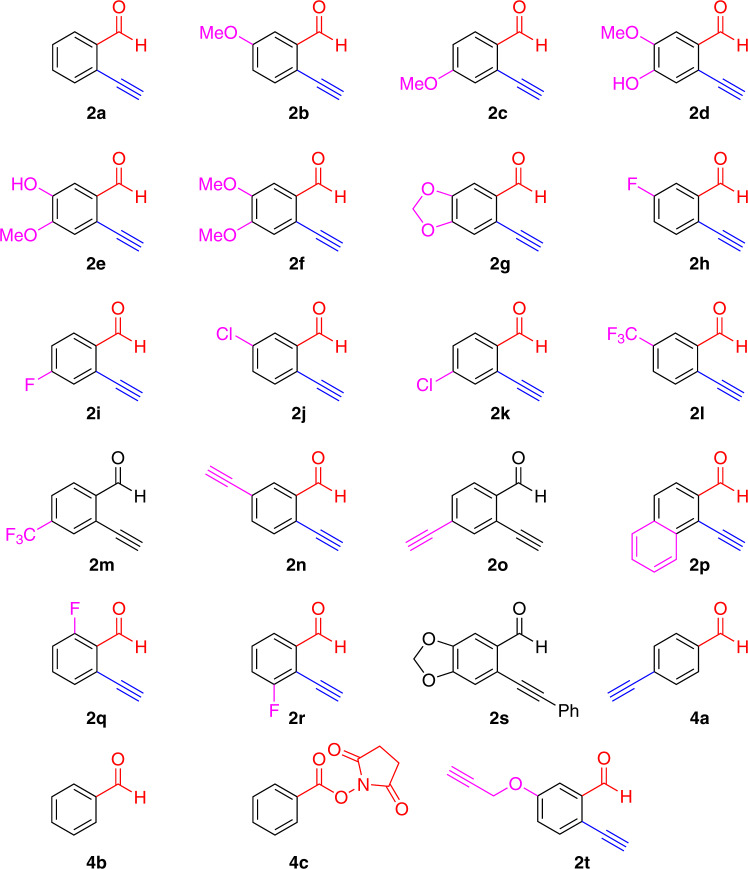
Substrate scope. Structures of 2-EBA **2a**–**t** and compounds **4a**–**c**.

With these promising findings, we next moved on to screen the peptide modification using a series of 2-EBA to improve the N-terminal selectivity of the modification and to study the structure-reactivity relationship of the 2-EBA (Table 1 and Fig. 3). In all, 2-EBA **2a** was commercially available and the others **2b–r** were easily prepared by Sonogashira coupling reaction of the commercially available aromatic halides with trimethylsilylacetylenes, followed by desilylation^34^. Screening reactions in 50 mM PBS at pH 6.5 indicated that the reaction could be conducted with good to high conversions (up to 86%) with excellent N-terminal selectivity (up to > 99:1). 2-EBA bearing electron-donating groups at 5- or 4-positions (**2b–g**) gave the highest conversions (up to 86%) with excellent N-terminal selectivity (up to >99:1) (Table 1, entries 2–7). Comparable conversions (up to 78%) and high N-terminal selectivity (up to >99:1) were obtained when using 2-EBA with weakly electron-withdrawing groups (fluoro or chloro) at 5- or 4- positions (**2h–k**, entries 8–11). Employment of 2-EBA with strongly electron-withdrawing groups (trifluoromethyl) at 5- or 4- positions (**2l** and **2m**) lead to moderate conversions (65 and 72%) and lower N-terminal selectivity (95:5 and 93:7). Incorporation of an alkyne moiety on the 2-EBA (**2n** and **2o**) also gave 41–64% conversions with up to 96:4 of N-terminal selectivity, indicating that the present reaction has high compatibility with unsaturated C-C bond (Entries 14–15). Modification using 1-ethynyl-2-naphthaldehyde (**2p**) gave poor conversion (5%), which was probably attributed to the poor solubility of the compound (Entry 16). Incorporation of fluoro group at 6-position (**2q**) resulted in high N-terminal selectivity (>99:1) but the high proportion of the di-modified products (74%) hampered its application (Entry 17). Moreover, introduction of fluoro group at 3-position (**2r**) lead to a lower N-terminal selectivity (93:7) (Entry 18). These findings revealed that 2-EBA were promising reagents for selective modification of the peptide N-terminus and incorporation of electron-donating groups or weakly electron-withdrawing groups would give high N-terminal selectivity of the modification.

**Table 1:** Screening of 2-EBA **2a–t** and compounds **4a–c** for modification of YTSSSKNVVR **1a**^a^.

Entry	Reagent	Conversion (%)^b^				N-terminal selectivity of mono-modified peptide^d^	Efficiency of N-terminal modification^e^
		Mono-modified^c^		Di-modified	Total		
		N-terminus	Lysine				
1	**2a**	61	3	8	72	96:4	0.85
2	**2b**	64	2	6	71	98:2	0.90
3	**2c**	60	3	5	68	96:4	0.88
4	**2d**	73	-	13	86	>99:1	0.85
5	**2e**	58	2	7	67	96:4	0.87
6	**2f**	27	1	-	28	97:3	0.96
7	**2g**	39	1	1	41	98:2	0.95
8	**2h**	64	-	4	68	>99:1	0.94
9	**2i**	69	2	7	78	98:2	0.88
10	**2j**	46	1	2	49	97:3	0.94
11	**2k**	53	2	3	58	97:3	0.91
12	**2l**	64	3	5	72	95:5	0.89
13	**2m**	58	4	3	65	93:7	0.89
14	**2n**	52	2	3	57	96:4	0.91
15	**2o**	56	3	5	64	95:5	0.88
16	**2p**	4.7	0.4	–	5	93:7	0.94
17	**2q**	26	–	74	100	>99:1	0.26
18	**2r**	65	5	25	95	93:7	0.68
19	**2s**	–	–	–	–	–	–
20	**4a**	–	–	–	–	–	–
21	**4b**	–	–	–	–	–	–
22^f^	**4c**	55	20	25	100	73:28	0.55
23	**2t**	70	1	9	80	98:2	0.88

^a^Conditions: YTSSSKNVVR **1a** (0.1 mM) and reagent (2 mM) in 50 mM PBS (pH 6.5)/DMSO (9:1) solution (100 μL), 37 °C, 16 h.

^b^Determined by total ion count (TIC) of LC–MS analysis.

^c^Conversion of N-terminal modified peptide (or lysine-modified peptide) is determined by the conversion of mono-modified peptide and N-terminal selectivity of mono-modified peptide.

^d^N-terminal selectivity is obtained by ratio of mono-modified peptide at N-terminal *α*-amino group to lysine *ε*-amino group as determined by extracted ion chromatogram (EIC) of LC-MS analysis.

^e^Efficiency of N-terminal modification is equal to the conversion of N-terminal modified peptide over the total conversion.

^f^Modification was conducted at room temperature for 15 min.

We have also conducted some control experiments (Table 1 and Fig. 3). No reaction was observed when 2-EBA **2s** bearing an internal alkyne was used which was ascribed to the lower reactivity of the internal alkyne for the intramolecular cyclization (Table 1, entry 19). Using 4-ethynylbenzaldehyde (**4a**) and benzaldehyde (**4b**) gave no peptide conversion suggesting that a terminal ethynyl group located at the *ortho*-position of benzaldehyde played a key role for isoquinolinium formation to give the resulting conjugate (Entries 20–21). We have also compared our reagents with the N-(benzoyloxy)succinimide (**4c**) which was widely used for amine modification (Entry 22). It was found that N-(benzoyloxy)succinimide (**4c**) was highly reactive towards the amine groups on the peptide and poor N-terminal selectivity (73:28) was achieved even though we conducted the modification in a slightly acidic condition (pH 6.5), indicating that 2-EBA displayed unique properties towards N-terminal modification.

With the aforementioned findings, we designed and synthesized 2-EBA **2t** for N-terminal modification of the peptides and proteins. The propargyl ether structure would provide an electron-donating effect towards the 2-EBA core structure to improve the N-terminal selectivity, while the free alkyne moiety allows attachment of versatile functional tags after the modification. Treatment of **2t** with peptide **1a** gave the high conversion (80%) with good N-terminal selectivity (98:2), suggesting that it would be a promising reagent for N-terminal modification of peptides and proteins.

### Optimization of reaction conditions

We next sought to examine the effects of the reagent amount, temperature, and pH values of the media on the N-terminal selectivity of the modification. With the highest N-terminal selectivity (Table 1, entry 5), 2-EBA **2d** was selected for further screening reactions. Different concentrations of **2d** were employed in the present reaction at 37 °C (Supplementary Table 2). Good conversion (86%) was observed using 20 equivalents of **2d** with excellent N-terminal selectivity (>99:1). When the reaction temperature was reduced to 25 °C and 4 °C respectively, lower conversions were observed. Noticeably, poor N-terminal selectivity (60:40) was found at 4 °C, suggesting that the low temperature would favor the reaction of the 2-EBA with the less hindered lysine *ε*-amino group.

**Fig. 4:**
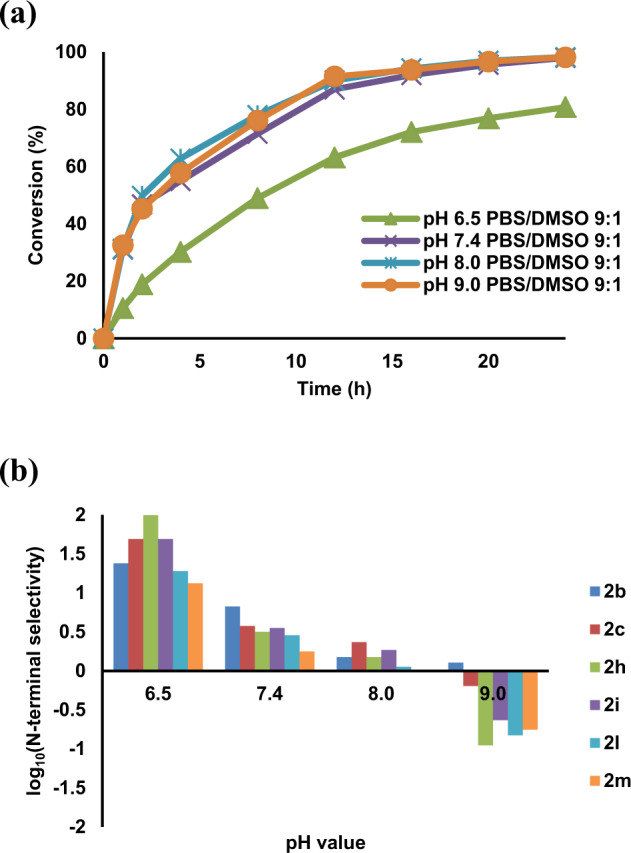
Effect of pH and substrate substituents. **a** Time course of N-terminal modification of YTSSSKNVVR **1a** using **2d** at pH 6.5–9.0. **b** Electronic effects of the substituents on 2-EBA towards the N-terminal modification of **1a** at pH 6.5–9.0.

We conducted time course experiments to test the effect of pH values on the modification. Bioconjugation reactions of peptide YTSSSKNVVR **1a** with **2d** (20 equiv.) in different pH values of 50 mM PBS and DMSO (9:1) were studied (Fig. 4a). At pH 6.5, excellent N-terminal selectivity (>99:1) was observed. As pH increased from 7.4 to 9, an increasing amount of mono-internal lysine-modified peptide and di-modified peptide as well as lower N-terminal selectivity were found at higher pH, indicating that the N-terminal selectivity of the modification was strongly influenced by the pH effects. Besides, the present reaction was optimized at 16 h to reach the highest conversion during the time course experiments.

To provide more insights on electronic effects of the substituents on the phenyl moieties of the 2-EBA towards the N-terminal selectivity of the modification, we performed the modification in different pH values of the media using 2-EBA with strongly electron-donating methoxy group (**2b** and **2c**), weakly electron-withdrawing fluoro group (**2h** and **2i**) as well as strongly electron-withdrawing trifluoromethyl group (**2l** and **2m**). As shown in Fig. 4b, by increasing the pH of the medium, the N-terminal selectivity of the modification decreased. Surprisingly, 2-EBA bearing electron-donating substituents still gave moderately N-terminal selective modification (**2b**) or weakly lysine selective modification (**2c**) at pH 9.0 PBS medium, while 2-EBA bearing electron-withdrawing substituents (**2h**, **2i**, **2l**, and **2m**) changed to give lysine selective modification. For example, modification of peptide **1a** with 2-EBA **2h** in pH 9.0 PBS/DMSO medium afforded mono-modified peptide with 9:81 of N-terminal selectivity, indicating the labeling was highly selective to the lysine *ε*-amino group. The above findings implicated that the site-selectivity of the modification was not only controlled by the pH effects but also by the electronic effects of the substituents on 2-EBA.

After screening of the effects towards the N-terminal modification, the stability of the bioconjugates was then studied by incubating the **2d**-modified YTSSSKNVVR with excess amount of reducing or oxidizing reagents (glutathione (GSH), homocysteine, l-cysteine, dl-dithiothreitol (DTT), 2-mercaptoethanol, tris(2-carboxyethyl)phosphine (TCEP), ascorbic acid, and hydrogen peroxide) in 50 mM PBS (pH 6.5)/DMSO (9:1) at 37 °C for 2 h (Supplementary Fig. 49). LC-MS/MS analysis revealed that the **2d**-modified YTSSSKNVVR was stable towards the additives with no significant decomposition or scrambling product.

### Screening of a peptide library

We next studied the applicability of this reaction on modification of a library of 20 unprotected peptides, XSKFR (X = either one of 20 natural amino acids). The peptide sequences with nucleophilic Ser and Lys were chosen for examination of the N-terminal selectivity of this reaction. As shown in Table 2, peptides with N-terminal Ala, Cys, Asp, Glu, Gly, His, Lys, Asn, Gln, Ser, or Tyr gave excellent N-terminal selectivity (>99:1) (Table 2, entries 1–11). Moderate-to-high N-terminal selectivities (86:14 to 98:2) were obtained for the N-terminal Ile, Leu, Trp, Phe, Val, Met, Thr, and Arg peptides (Entries 12–19). However, a low N-terminal selectivity of 46:54 was observed for PSKFR having N-terminal proline residue (Entry 20), which is presumably due to the iminium intermediate formed between proline and 2-EBA cannot undergo subsequent cyclization with the proximal alkyne group.

**Table 2:** Modification of a peptide library XSKFR using 2-EBA **2d**^a^.

Entry	Peptide	Conversion (%)^b^				N-terminal selectivity of mono-modified peptide^d^	Efficiency of N-terminal modification^e^
		Mono-modified^c^		Di-modified	Total		
		N-terminus	Lysine				
1^f^	CSKFR	73	–	0	73	>99:1	1.0
2	ASKFR	84	–	3	87	>99:1	0.97
3	YSKFR	56	–	2	58	>99:1	0.97
4	GSKFR	50	–	2	52	>99:1	0.96
5	HSKFR	70	–	4	74	>99:1	0.95
6	DSKFR	39	–	3	42	>99:1	0.93
7	ESKFR	32	–	3	35	>99:1	0.91
8	NSKFR	62	–	6	68	>99:1	0.91
9	SSKFR	42	–	6	48	>99:1	0.88
10	QSKFR	79	–	15	94	>99:1	0.84
11	KSKFR	54	–	13	67	>99:1	0.81
12	LSKFR	51	1	3	55	98:2	0.93
13	ISKFR	59	1	10	70	98:2	0.84
14	WSKFR	38	1	1	40	97:3	0.95
15	FSKFR	58	2	7	67	96:4	0.87
16	VSKFR	61	3	2	66	95:5	0.92
17	MSKFR	60	4	2	66	94:6	0.91
18	TSKFR	51	6	7	64	90:10	0.80
19	RSKFR	56	9	5	70	86:14	0.80
20	PSKFR	10	12	1	23	46:54	0.43

^a^Conditions: XSKFR **1a** (0.1 mM) and reagent **2d** (2 mM) in 50 mM PBS (pH 6.5)/DMSO (9:1) solution (100 μL), 37 °C, 16 h.

^b^Determined by total ion count (TIC) of LC–MS analysis.

^c^Conversion of N-terminal modified peptide (or lysine-modified peptide) is determined by the conversion of mono-modified peptide and N-terminal selectivity of mono-modified peptide.

^d^N-terminal selectivity is obtained by ratio of mono-modified peptide at N-terminal *α*-amino group to lysine *ε*-amino group as determined by extracted ion chromatogram (EIC) of LC–MS analysis.

^e^Efficiency of N-terminal modification is equal to the conversion of N-terminal modified peptide over the total conversion.

^f^Tris(2-carboxyethyl)phosphine (TCEP, 0.5 mM) was added to prevent the formation of disulfide linkage.

To further study the selectivity of the present bioconjugation reaction, we used peptides with cysteine at different positions (ASCGTN, AYEMWCFHQR, and KSTFC). Exclusive N-terminal modification with 70%, 27 and 15% conversions, respectively, was found with the cysteine residue remaining intact (Supplementary Figs. 77–79). In addition, sole modification at the internal lysine in an N-terminally acetylated peptide Ac-YTSSSKNVVR with 19% conversion was observed, indicating that the present bioconjugation reaction was highly chemoselective to the amino group of peptides as only the amino group of lysine was modified when the N-terminus is acetylated (Supplementary Fig. 80). For a peptide containing a second proline residue (YPSSSKNVVR) which has no reactivity towards 2-PCA^29^, it was found that the bioconjugation proceeded smoothly with **2d** to afford 54% conversion with excellent N-terminal selectivity (>99:1) (Supplementary Fig. 81).

### Protein modification using 2-ethynylbenzaldehydes

**Fig. 5:**
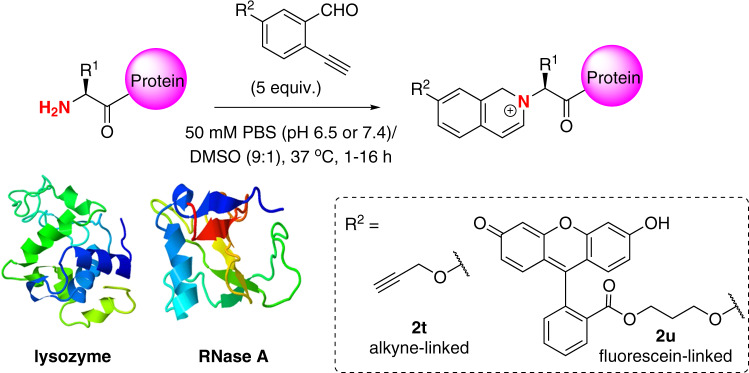
N-terminal protein modification. Modification of lysozyme and RNase A with functionalized 2-EBA **2t** and **2u**.

After studying the N-terminal peptide modification, we further explored the present reaction for protein modification (Fig. 5) employing alkyne-linked and fluorescein-linked 2-EBA (**2t** and **2u**, respectively). The presence of the ether linkage was to improve the N-terminal selectivity of the reagent, suggested by the aforementioned findings. In total, 0.1 mM of lysozyme (PDB ID: 1DPX) was treated with **2t** (0.5 mM, 5 equivalents) in 50 mM PBS (pH 6.5) at 37 °C for 16 h, giving the **2t**-modified lysozyme with 52% conversion (Supplementary Fig. 82a). LC–MS analysis of the reaction mixtures of lysozyme showed peaks at 14470 Da and 14489 Da, which were assigned to the mono-modified lysozyme. Upon trypsin digestion, the modification by **2t** was found to selectively occur at the N-terminus, as depicted by LC–MS/MS analysis of the tryptic peptide fragment of KVFGR (Supplementary Fig. 83). Increasing the pH to 7.4 in the N-terminal modification of lysozyme with **2t** generally resulted in excellent conversion (92%) (Supplementary Fig. 82b) with mono- and di-modified lysozyme products observed. Note that the N-terminal selectively is still considerably high as depicted in LC-MS/MS analysis. The reaction of RNase A (PDB ID: 3DH5) was performed at pH 7.4 to afford up to 44% protein conversion (Supplementary Figs. 84–85). Modification of lysozyme and RNase A with fluorescein-linked 2-EBA **2u** in 50 mM PBS (pH 7.4) gave 34 and 10% conversions with high N-terminal selectivity, respectively (Supplementary Figs. 86–87). As the imidazolidinone conjugate of the 2-PCA-modified RNase A was found to have partially (20–30%) decomposition at 37 °C after 12 h, we also tested the stability of the **2t**-modified RNase A by treatment of the modified RNase A in PBS with different pH values (pH 3–11) at 37 °C (Supplementary Fig. 88). Noticeably, no decomposition was found by LC-MS analysis, indicating that the quinolinium conjugate formed was highly stable and this modification would be amenable for preparation of bioconjugates for drug development.

Human arginase, which is a manganese-dependent enzyme that degrades arginine into urea, has been reported to treat advanced hepatocellular carcinoma and metastatic melanoma where prior immunotherapy failed in early phase of clinical trial^35^. PEGylated human arginase I was developed as the first generation of therapeutic proteins with long half-life, which is now undergoing phase II clinical trials^36^. However, current PEGylation usually involved non-specific lysine/cysteine modification via NHS/maleimide chemistry. The second-generation therapeutic protein, *Bacillus caldovelox* arginase mutant (BCArg mutant), has been reported to induce a sustained complete remission in a patient with immunotherapy-resistant cancer^37,38^. In addition to lysozyme and RNase A, we also extended this newly developed N-terminal modification to modify the BCArg mutant. BCArg mutant (0.1 mM) was treated with alkyne- and fluorescein-linked 2-EBA (**2t** and **2u**, 10 equivalent) in PBS (pH 7.4)/DMSO (9:1) at 37 °C for 16 h to give the corresponding **2t**- and **2u**-modified BCArg mutant in 40 and 19% conversions, respectively (Supplementary Fig. 89). High N-terminal selectivity as revealed by the tryptic peptide fragments MKPISIIGVPMDLGQTR of **2t**- and **2u**-modified BCArg mutants were observed by LC-MS/MS analysis (Supplementary Fig. 90).

**Fig. 6:**
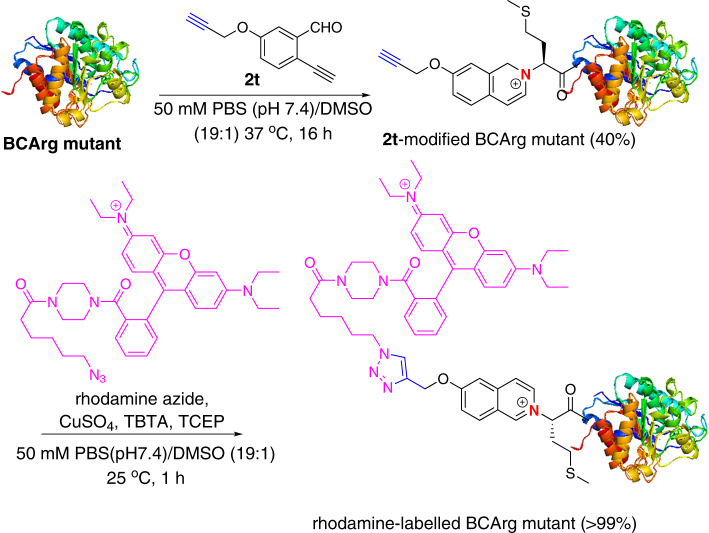
N-terminal protein modification with an alkyne. Modification of the BCArg mutant with alkyne-functionalized 2-EBA **2t** and sequential copper(I)-catalyzed [3 + 2] cycloaddition reaction with rhodamine azide.

As depicted in Fig. 6, the **2t**-modified BCArg mutant containing an alkyne handle (32615 Da) could be smoothly modified with a rhodamine-azide *via* copper(I)-catalyzed [3 + 2] cycloaddition reaction to give the rhodamine-labeled BCArg mutant (33268Da and 33296Da) in >99% conversion (Supplementary Fig. 91). SDS-PAGE analysis revealed that the rhodamine-labeled BCArg mutant gave a strongly green fluorescent signal while the **2t**-modified BCArg mutant had no fluorescent signal at UV 365 nm (Supplementary Fig. 92). Coomassie blue staining on the same gel gave deep blue color signals of unmodified, alkyne-linked as well as rhodamine-labeled proteins, indicating that the fluorescent tag has been successfully labeled on the proteins using the N-terminal selective alkyne-linked 2-EBA **2t** and a sequential azide-alkyne click reaction. These results indicated that the present reaction has high compatibility with click chemistry.

### Biological studies of N-terminally modified BCArg mutants

To study the influence of the quinolinium conjugates on the biological properties of the therapeutic protein, we compared the enzyme activities and anti-cancer properties of the modified BCArg mutant with the unmodified analogue (Table 3). We prepared **2b**-modified BCArg mutant (32%) by bioconjugation with 2-EBA **2b** in 50 mM PBS (pH 7.4) at 37 °C for 16 h (Supplementary Figs. 93–94). The enzymatic properties of the **2b**-modified BCArg mutant was slightly lower than that of the unmodified BCArg mutant. The anti-cancer properties of the unmodified and **2b**-modified BCArg mutants were then examined using breast cancer cell lines MDA-MB-231 and MDA-MB-468. Experimental IC_50_ values indicated that the antitumor efficacy of the **2b**-modified BCArg mutant was comparable to that of the unmodified one. These findings indicated that the **2b**-modified BCArg mutant retained its biological activities after the bioconjugation.

**Table 3:** Enzymatic activities and IC_50_ values of BCArg mutant and **2b**-modified BCArg mutant.

Sample	BCArg mutant	**2b**-modified BCArg mutant
Specific activity (U/mg)	443.96 ± 27.50	313.68 ± 4.01
IC_50_ values for MDA-MB-231 (U/mL)	4.608 ± 0.995	5.491 ± 1.564
IC_50_ values for MDA-MB-468 (U/mL)	4.772 ± 1.137	5.631 ± 2.410

In summary, we have discovered that 2-ethynylbenzaldehydes (2-EBA) are a useful reagent for N-terminal modification of peptides and proteins via isoquinolinium formation with the N-terminal *α*-amino group. After a comprehensive screening of the reaction conditions and the structure-reactivity relationship of the 2-EBA, we have found that apart from the pH control, the electronic properties of the substituents on the 2-EBA could also strongly affect the N-terminal selectivity of the modification. Under slightly acidic condition (pH 6.5) and employing 2-EBA with electron-donating and weakly electron-withdrawing groups, the modification has achieved excellent N-terminal selectivity. Conducting the reaction in basic medium (pH 9) and using 2-EBA with electron-withdrawing groups can switch the modification to become lysine selective. To help other researchers who are interested in using this bioconjugation reaction, the reaction conditions, substituent effects, and functional group tolerance for various N-terminal residues are summarized in Table 4. The present method is successfully applied on selective modification of proteins, including the therapeutic protein (BCArg mutant). The resulting modified bioconjugates are of good stability and the biological properties of the modified BCArg mutant are comparable to those of the unmodified one.

**Table 4:** Guide for using the 2-ethynylbenzaldehydes for N-terminal selective modification.

Reaction conditions	Peptides	Proteins
Equivalent of 2-EBA	20 equivalents	≤10 equivalents
pH	6.5	6.5–7.4
Reaction temperature	37 °C	
Reaction time	≤16 h	
Substituent effects of 2-EBA	N-terminal selectivity is excellent (up to >99:1) when 2-EBA with electron-donating group at 5- or 4-positions.	
Functional group tolerance	Highly compatible with 19 of the 20 natural amino acids, except for N-terminal proline.	

## Methods

### Synthesis and characterization

The synthetic procedures and characterization for compounds as well as chromatography and mass spectrometry data are presented in in Supplementary Methods, Supplementary Figs. 1–48, and Supplementary Figs. 50–76.

### General procedure for modification of peptides using 2-ethynylbenzaldehydes

To an eppendorf tube (1.5 mL) with 80 µL of 50 mM PBS buffer pH 6.5, 10 µL of YTSSSKNVR (**1a**, 1 mM in Milli-Q® water) was added to the buffer, followed by 10 µL of 2-ethynylbenzaldehyde (**2a–2t**, 20 mM in DMSO). The reactive mixture was allowed to react in a 37 °C water bath for 16 h. 10 µL of the mixture was drawn, diluted with 10 µL of Milli-Q® water and subjected to LC/MS–MS analysis.

Unless otherwise specified, all peptides were treated as same as the above procedure.

### Reporting summary

Further information on research design is available in the Nature Research Reporting Summary linked to this article.

## Supplementary information

Supplementary Information

Reporting Summary

## Data Availability

All principal data with detailed experimental procedure and characterization of this work are included in this article, and its Supplementary Information or are available from the corresponding author upon reasonable request.
