# Supplementary material for: N-Terminal selective modification of peptides and proteins using 2-ethynylbenzaldehydes
Source: Commun Chem. 2020 May 29;3:67. doi: 10.1038/s42004-020-0309-y (PMC9814395; doi:10.1038/s42004-020-0309-y)
Supplement: Supplementary file 1 — Supplementary Information [file 42004_2020_309_MOESM1_ESM.pdf]

N-Terminal Selective Modification of Peptides and Proteins using 2-Ethynylbenzaldehydes

Jie-Ren Deng,[+,1] Nathanael Chun-Him Lai,[+,1] Karen Ka-Yan Kung,[1,*] Bin Yang,[1] Sai-Fung Chung,[2] Alan Siu-Lun Leung,[2] Man-Chung Choi,[2] Yun-Chung Leung[2,*] and Man-Kin Wong[1,*]

[+]These authors contributed equally to this work

[1]State Key Laboratory of Chemical Biology and Drug Discovery, and Department of Applied Biology and Chemical Technology, The Hong Kong Polytechnic University, Hung Hom, Hong Kong, China.

[2]Henry Cheng Research Laboratory for Drug Development and Lo Ka Chung Centre for Natural Anti-Cancer Drug Development, The Hong Kong Polytechnic University, Hung Hom, Hong Kong, China.

E-mail: mankin.wong@polyu.edu.hk thomas.yun-chung.leung@polyu.edu.hk

karen.kung@polyu.edu.hk

Supporting Information

Supplementary Table 1. Literature References

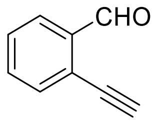 **2a**	Ouyang, H. C.; Tang, R. Y.; Zhong, P.; Zhang, X. G.; Li, J. H. *J. Org. Chem.*, **2011**, *76*, 223.
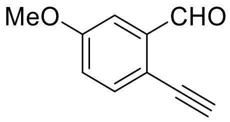 **2b**	Ohta, Y.; Kubota, Y.; Watabe, T.; Chiba, H.; Oishi, S.; Fujii, N.; Ohno, H. *J. Org. Chem.*, **2009**, *16*, 6299.
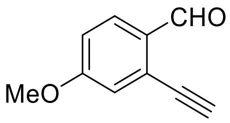 **2c**	Li, S. Y.; Luo, Y.; Wu, J. *Org. Lett.*, **2011**, *13*, 3190.
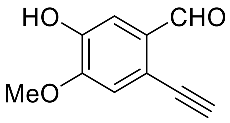 **2e**	Cao, Z. P.; Zhang, H. Q.; Zhang, X. X.; Zhang, L. D.; Meng, X.; Chen, G.; Zhao, X.-E.; Sun, X. J.; You, J. M. *RSC Adv.*, **2015**, *5*, 103155.
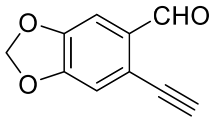 **2g**	Takahashi, H.; Yoshida, K.; Yanagisawa, A. *J. Org. Chem.*, **2009**, *74*, 3632.
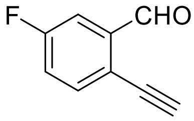 **2h**	Ohta, Y.; Kubota, Y.; Watabe, T.; Chiba, H.; Oishi, S.; Fujii, N.; Ohno, H. *J. Org. Chem.*, **2009**, *16*, 6299.
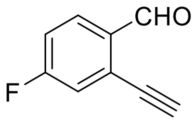 **2i**	Ohta, Y.; Kubota, Y.; Watabe, T.; Chiba, H.; Oishi, S.; Fujii, N.; Ohno, H. *J. Org. Chem.*, **2009**, *16*, 6299.
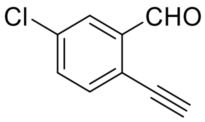 **2j**	Li, M. M.; Xing, P.; Huang, Z. G.; Jiang, B. *Chin. J. Chem.*, **2013**, *31*, 49.

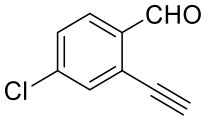 **2k**	Wu, X.; Li, M. L.; Chen, D. F.; Chen, S.-S. *J. Org. Chem.*, **2014**, *79*, 4743.
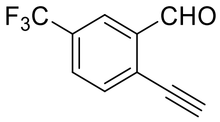 **2l**	He, J.; Shi, Y. P.; Cheng, W. L.; Man, Z. M.; Yang, D. D.; Li, C. Y. *Angew. Chem., Int. Ed.*, **2016**, *55*, 4557.
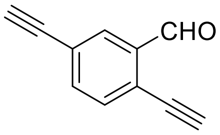 **2n**	Zhou, N. Z.; Wang, L.; Thompson, D. W.; Zhao, Y. M. *Tetrahedron*, **2011**, *67*, 125.
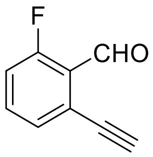 **2q**	He, J.; Shi, Y. P.; Cheng, W. L.; Man, Z. M.; Yang, D. D.; Li, C. Y. *Angew. Chem., Int. Ed.*, **2016**, *55*, 4557.
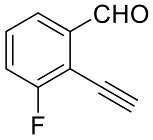 **2r**	He, J.; Shi, Y. P.; Cheng, W. L.; Man, Z. M.; Yang, D. D.; Li, C. Y. *Angew. Chem., Int. Ed.*, **2016**, *55*, 4557.
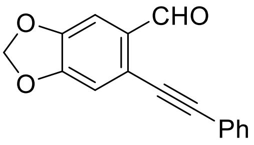 **2s**	Huang, Q. H.; Hunter, J. A.; Larock, R. C. *J. Org. Chem.*, **2002**, *67*, 3437.

Supplementary Methods

General Procedure

All reagents were commercially available and used without further purification. Milli-Q® water used as reaction solvent in peptide and protein modification, as well as LC-MS analysis, was deionised using a Milli-Q® Gradient A10 system (Millipore, Billerica, USA). Flash column chromatography was performed using silica gel 60 (230-400 mesh ASTM) with *n*-hexane/ethyl acetate or dichloromethane/methanol as eluent. ^{1}H and ^{13}C NMR spectra were recorded on a Bruker DPX-400 spectrometer. All chemical shifts are quoted on the scale in ppm using TMS or residual solvent as the internal standard. Coupling constants (J) are reported in Hertz (Hz) with the following splitting abbreviations: s = singlet, br s = broad singlet, d = doublet, dd = double doublet, t = triplet and m = multiplet. All the mass spectra were obtained on an ESI source of Agilent 6540 Ultra High Definition (UHD) Accurate-Mass Q-TOF LC/MS systems in the positive ion mode.

ESI–MS Analysis of Peptide Modification

LC-MS analyses for peptide identification were performed by using an Agilent 6540 UHD Accurate-Mass Q-TOF LC/MS system equipped with an ion spray source and an Agilent 1290 Infinity LC, using an Agilent ZORBAX RRHD SB300-C18 (1.8 μm, 2.1 x 100 mm) column. 3 μL of sample was injected with a flow rate of the flow rate was 0.2 mL/min. Mobile phase A was made of Milli-Q® water containing 0.1% formic acid. Mobile phase B was made of HPLC grade acetonitrile containing 0.1% formic acid. The initial conditions for separation were 5% B for 3 min, followed by a linear gradient to 95% B by 17 min. The composition was maintained for 10 min, followed by a linear gradient to 5% B by 1 min. The composition was maintained for 4 min. Operating conditions optimized for the detection of reaction mixture were the following: Gas temperature: 300 ^{o}C, Drying gas: 8 L/min, Nebulizer: 35 psig, Sheath gas temperature: 270 ^{o}C, Sheath gas flow: 11 L/min, VCap: 3500 V, Nozzle voltage: 1000 V.

ESI–MS Analysis of Protein Modification

LC-MS analyses for protein identification were performed by using an Agilent 6540 UHD Accurate-Mass Q-TOF LC/MS system equipped with an ion spray source and an Agilent 1290 Infinity LC, using an Agilent ZORBAX RRHD SB300-C3 (1.8 μm, 2.1 x 100 mm) column. 3 μL of sample was injected with a flow rate of the flow rate was 0.2 mL/min. Mobile phase A was made of Milli-Q® water containing 0.1% formic acid. Mobile phase B was made of HPLC grade acetonitrile containing 0.1% formic acid. The initial conditions for separation were 5% B for 3 min, followed by a linear gradient to 95% B by 17 min. The composition was maintained for 10 min, followed by a linear gradient to 5% B by 1 min. The composition was maintained for 4 min. Operating conditions optimized for the detection of reaction mixture were the followings: Gas temperature: 300 ^{o}C, Drying gas: 8 L/min, Nebulizer: 35 psig, Sheath gas temperature: 350 ^{o}C, Sheath gas flow: 11 L/min, VCap: 3500 V, Nozzle voltage: 1000 V.

General Procedure for Preparation of 2-Ethynylbenzaldehydes 2a-2r[1]

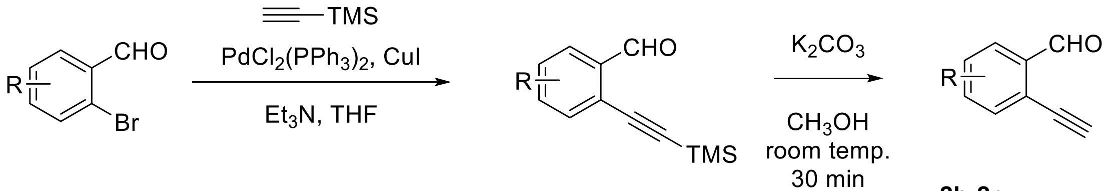

2b'-2r'
yield (56-95%)

2b-2r
yield (80-99%)

To a Schlenk flask with bis(triphenylphosphine)palladium(II) dichloride (5 mol%) and copper iodide (2.5 mol%) under N_2 atmosphere, a solution of *o*-bromobenzaldehyde derivatives (5 mmol) in tetrahydrofuran (50 mL) and trimethylamine (0.4 M) was added at room temperature, followed by trimethylsilylacetylene (6 mmol, 1.2 equiv.). The mixture was then heated at 80 ^{o}C for overnight. After filtration of the mixture with celite, the filtrate was concentrated under reduced pressure and the residue was purified by column chromatography over silica gel with *n*-hexane/ethyl acetate (50:1) as the eluent to give the corresponding reaction intermediate **2b'-2r'**.

The corresponding reaction intermediate (**2b'-r'**) was reacted with potassium carbonate (0.50 g, 3.64 mmol) in methanol (30 mL) for 30 min at room temperature, and the solvent was removed under reduced pressure. The residue was extracted with dichloromethane and washed with saturated aqueous sodium carbonate, and dried over $MgSO_4$. The filtrate was concentrated under reduced pressure and purified by flash column chromatography over silica gel with *n*-hexane/ethyl acetate (50:1) to give 2-ethynylbenzaldehyde derivatives **2b-2r**.

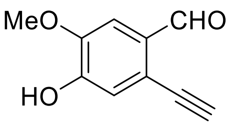

2-Ethynyl-4-hydroxy-5-methoxybenzaldehyde (2d)

White solid, 45% yield

^{1}H NMR (400 MHz, $(CD_3)_2SO$): δ 10.37 (s, 1H), 7.42 (s, 1H), 7.11 (s, 1H), 6.15 (s, 1H), 3.96 (s, 3H), 3.35 (s, 1H). **^{13}C NMR** (100 MHz, $(CD_3)_2SO$): δ 189.54, 152.96, 149.43, 129.43, 119.80, 119.64, 109.61, 86.21, 79.53, 56.16. HRMS (ESI): calcd. for $C_{10}H_9O_3$ $(M + H)^+$: 177.0552, found: 177.0543.

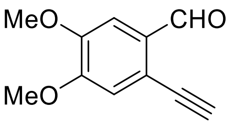

2-Ethynyl-4,5-dimethoxybenzaldehyde (2f)

White solid, 70% yield

^{1}H NMR (400 MHz, $CDCl_3$): δ 10.39 (s, 1H), 7.40 (s, 1H), 7.02 (s, 1H), 3.96 (s, 3H), 3.95 (s, 3H), 3.38 (s, 1H). **^{13}C NMR** (100 MHz, $CDCl_3$): δ 190.19, 153.53, 150.12, 130.99, 120.07, 114.94, 108.23, 82.85, 79.17, 56.23, 56.15. HRMS (ESI): calcd. for $C_{11}H_{11}O_3$ $(M + H)^+$: 191.0630, found: 191.0700.

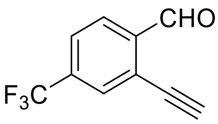

2-Ethynyl-4-(trifluoromethyl)benzaldehyde (2m)

Pale yellow solid, 66% yield

^{1}H NMR (400 MHz, CD_3CN): δ 10.51 (s, 1H), 8.03 (d, J = 8.2 Hz, 1H), 7.97 (s, 1H), 7.83 (d, J = 8.1 Hz, 1H), 3.97 (s, 1H). **^{13}C NMR** (100 MHz, CD_3CN): δ 190.28, 139.00, 134.36 (q, J = 32.9 Hz), 130.84 (q, J = 3.9 Hz), 128.17, 126.03 (q, J = 3.6 Hz), 125.64, 123.29 (q, J = 272.4 Hz), 86.65, 77.59. **^{19}F NMR** (376 MHz, CD_3CN): δ -64.03. HRMS (ESI): calcd. for $C_{10}H_6F_3O$ $(M + H)^+$: 199.0371, found: 199.0359.

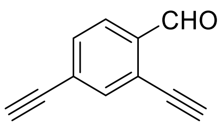

2,4-Diethynylbenzaldehyde (2o)

White solid, 75% yield

^{1}H NMR (400 MHz, $CDCl_3$): δ 10.48 (s, 1H), 7.86 (d, *J* = 7.9 Hz, 1H), 7.69 (s, 1H), 7.53 (d, *J* = 7.7 Hz, 1H), 3.48 (s, 1H), 3.30 (s, 1H). **^{13}C NMR** (100 MHz, $CDCl_3$): δ 190.49, 137.27, 135.97, 132.59, 127.92, 127.23, 125.57, 84.98, 81.73, 78.31. HRMS (ESI): calcd. for $C_{11}H_7O$ $(M + H)^+$: 155.0497, found: 155.0482.

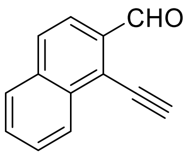

1-Ethynyl-2-naphthaldehyde (2p)

Pale yellow solid, 63% yield

^{1}H NMR (400 MHz, $CDCl_3$): δ 10.79 (s, 1H), 8.55 – 8.53 (m, 1H), 7.99 – 7.96 (m, 1H), 7.92 – 7.88 (m, 2H), 7.70 – 7.65 (m, 2H), 3.92 (s, 1H). **^{13}C NMR** (100 MHz, $CDCl_3$): δ 191.93, 135.66, 135.30, 133.42, 129.50, 129.44, 128.47, 127.89, 127.11, 126.03, 121.90, 90.10, 77.14. HRMS (ESI): calcd. for $C_{13}H_9O$ $(M + H)^+$: 181.0653, found: 181.0640.

^{1}H NMR

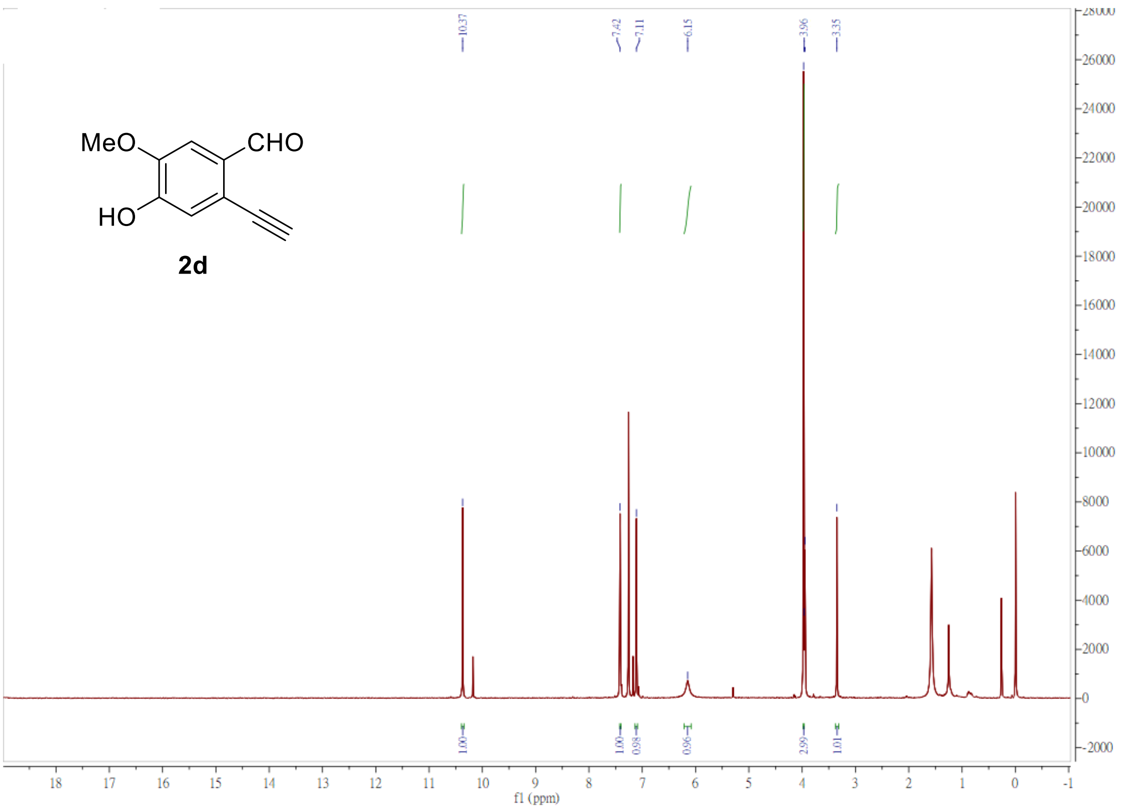

^{13}C NMR

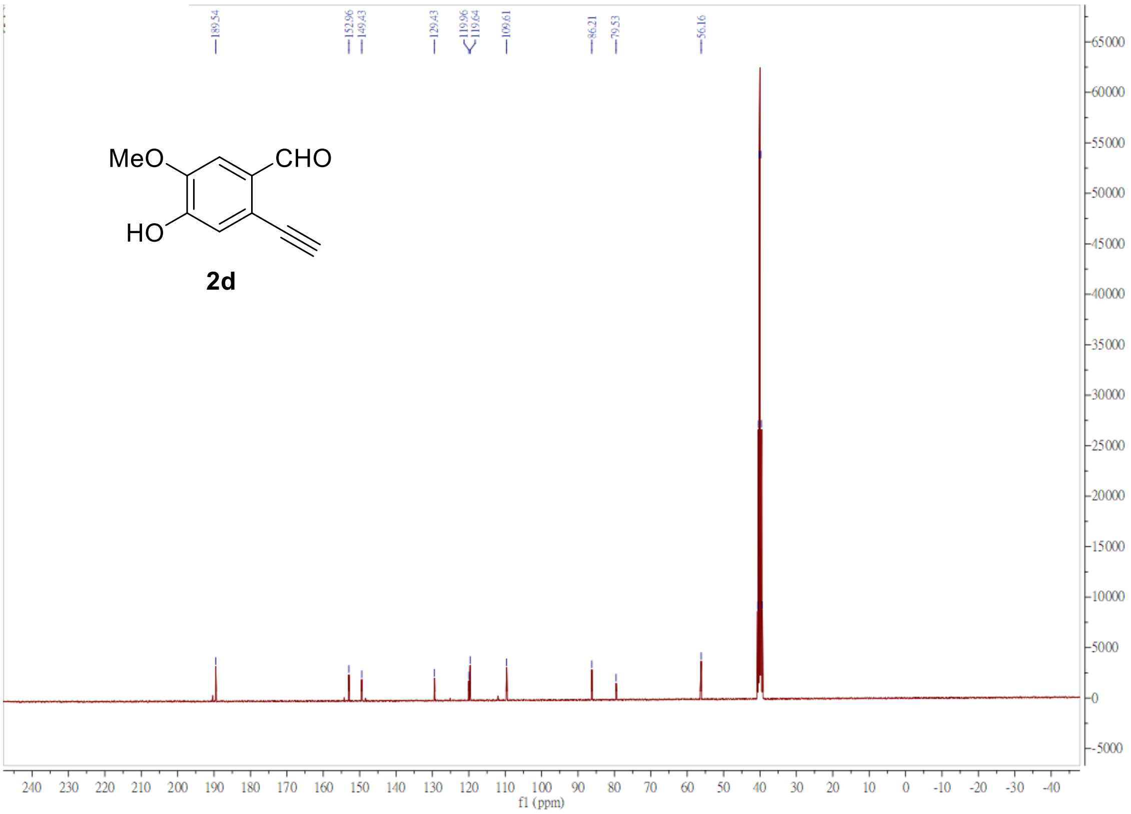

Supplementary Fig. 1 ^{1}H NMR and ^{13}C NMR spectra of compound **2d**.

^{1}H NMR

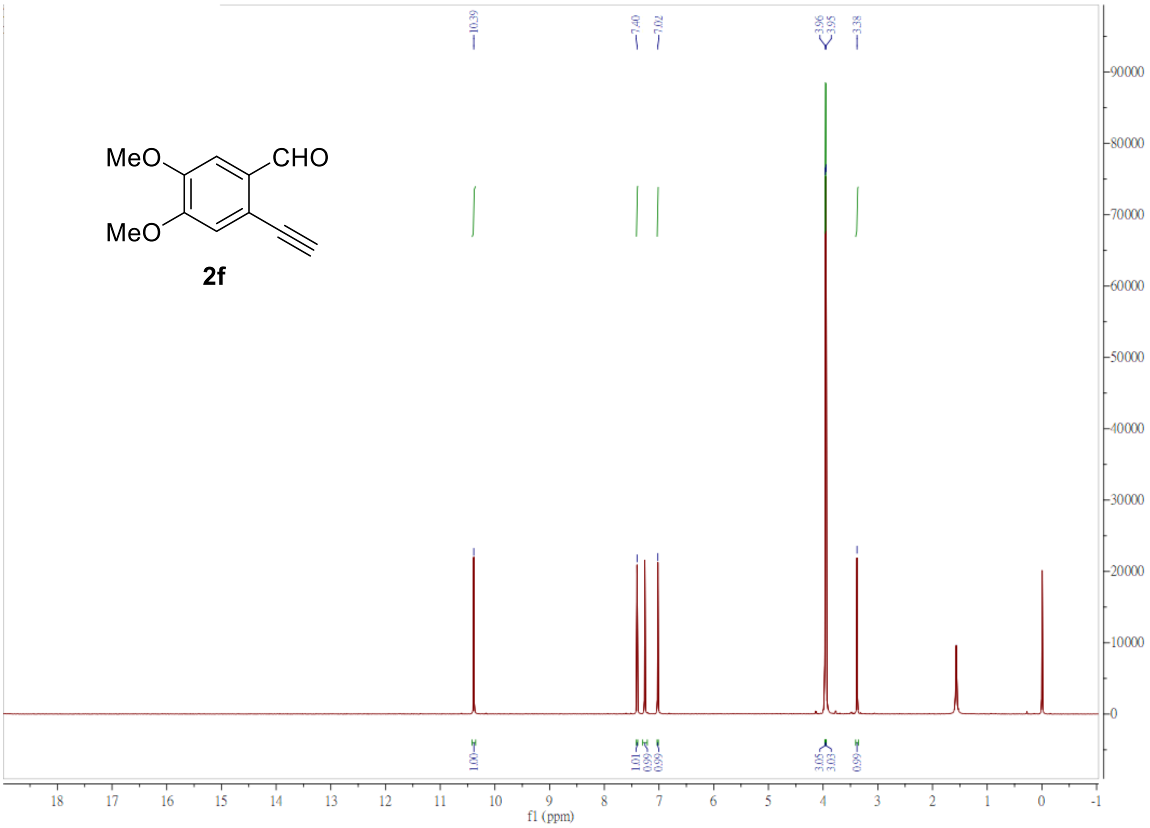

^{13}C NMR

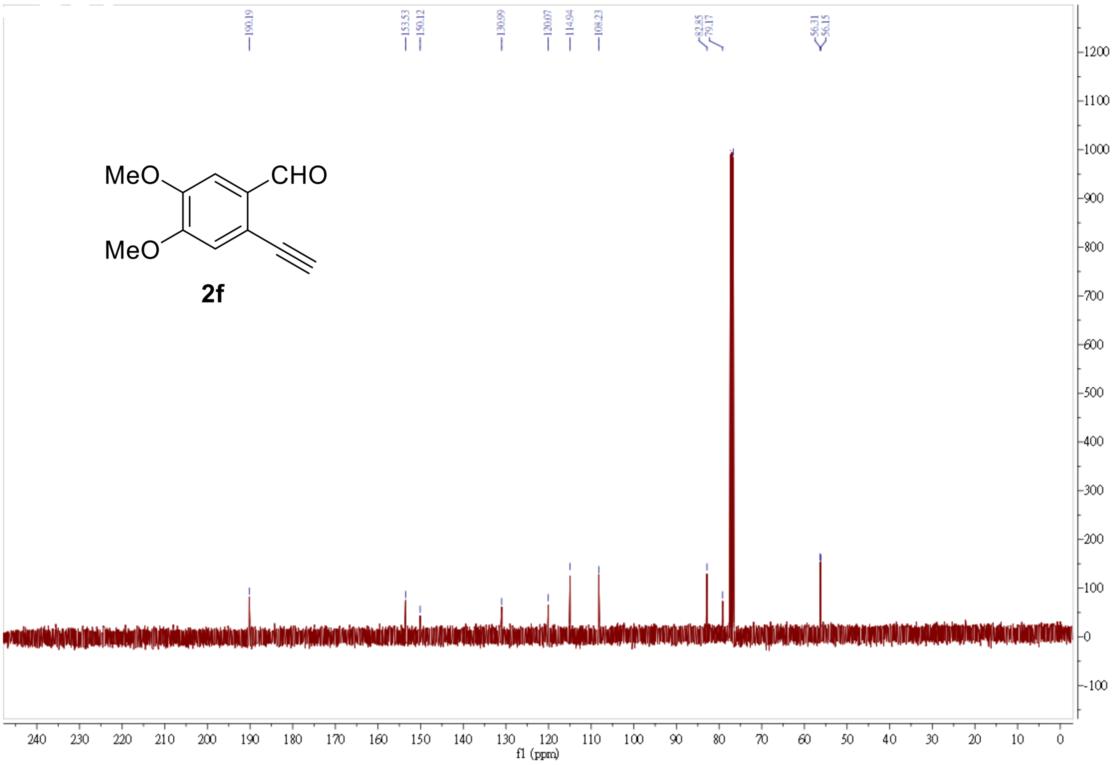

Supplementary Fig. 2 ^{1}H NMR and ^{13}C NMR spectra of compound **2f**.

^{1}H NMR

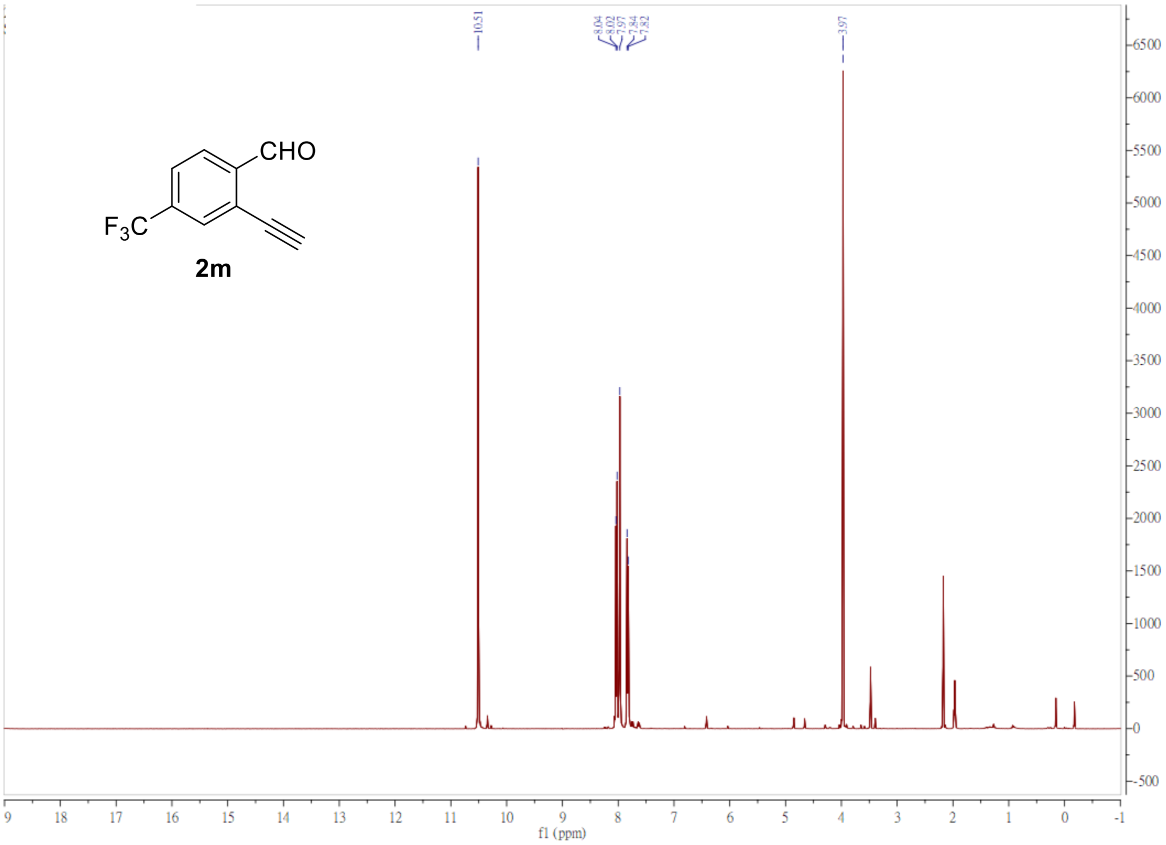

^{13}C NMR

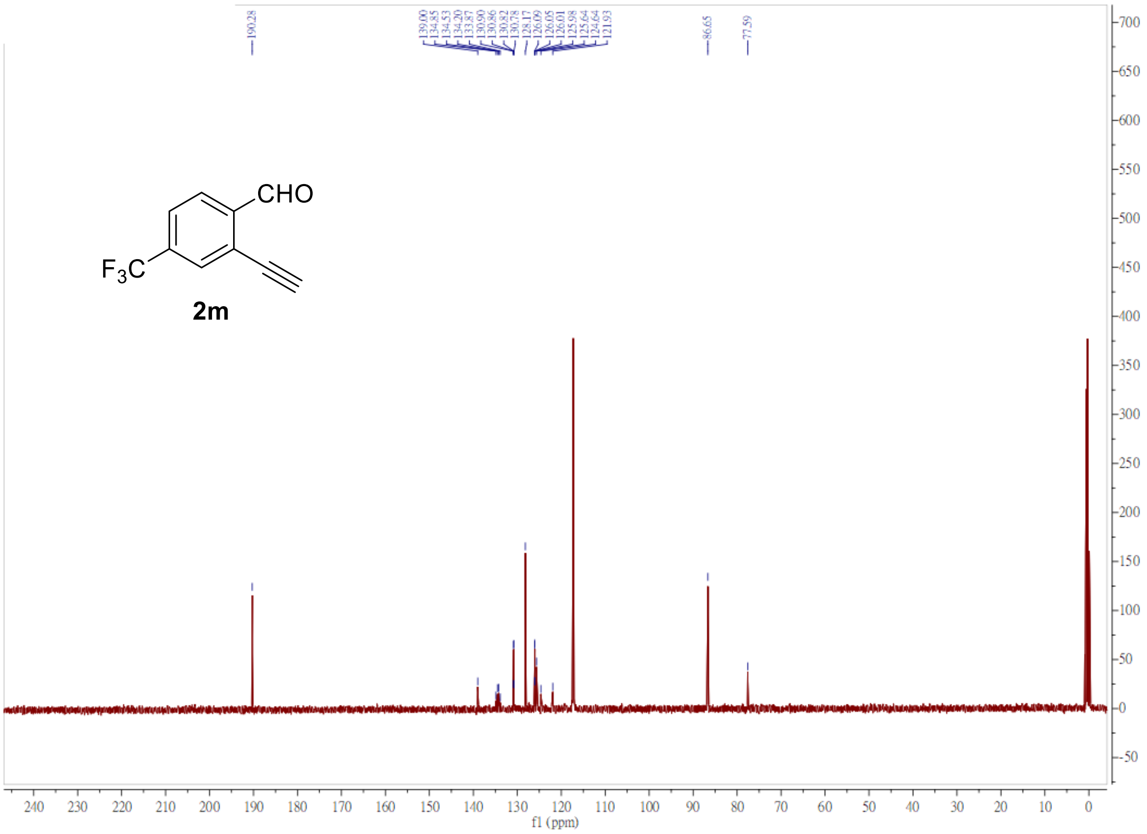

^{19}F NMR

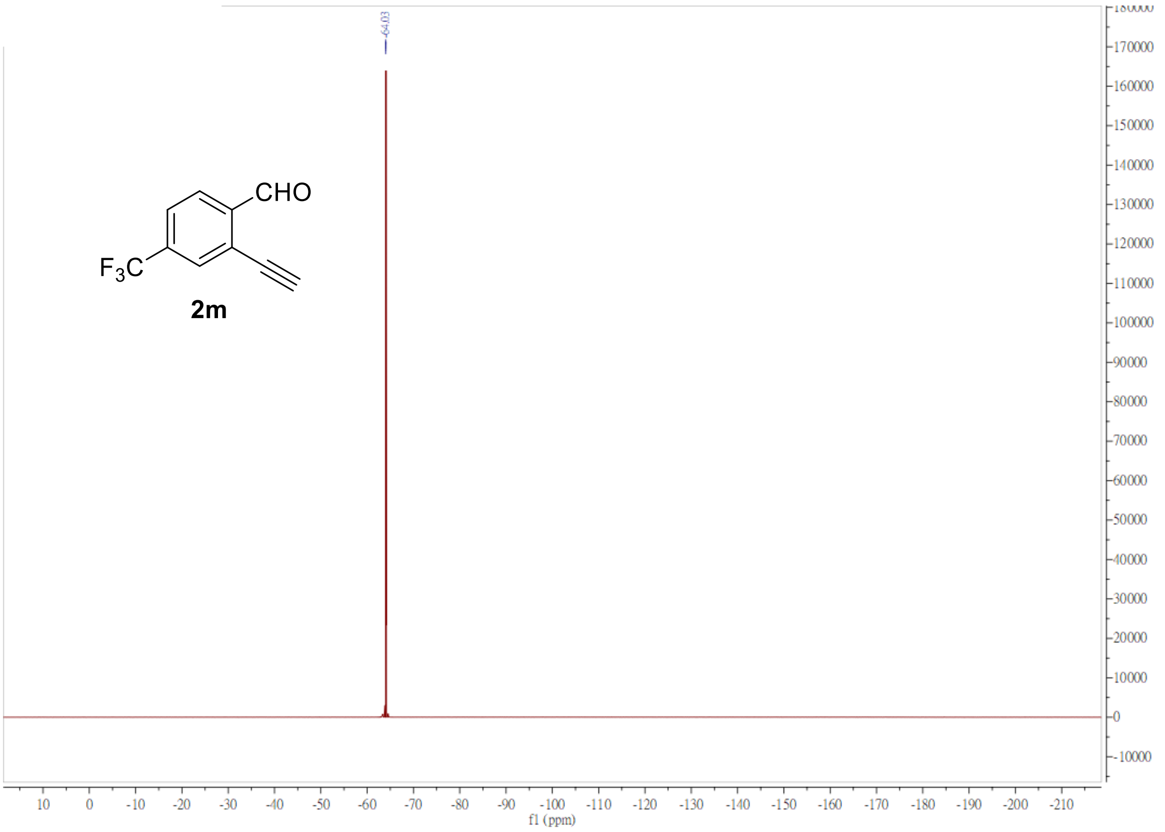

Supplementary Fig. 3 ^{1}H NMR, ^{13}C NMR and ^{19}F NMR spectra of compound **2m**.

^{1}H NMR

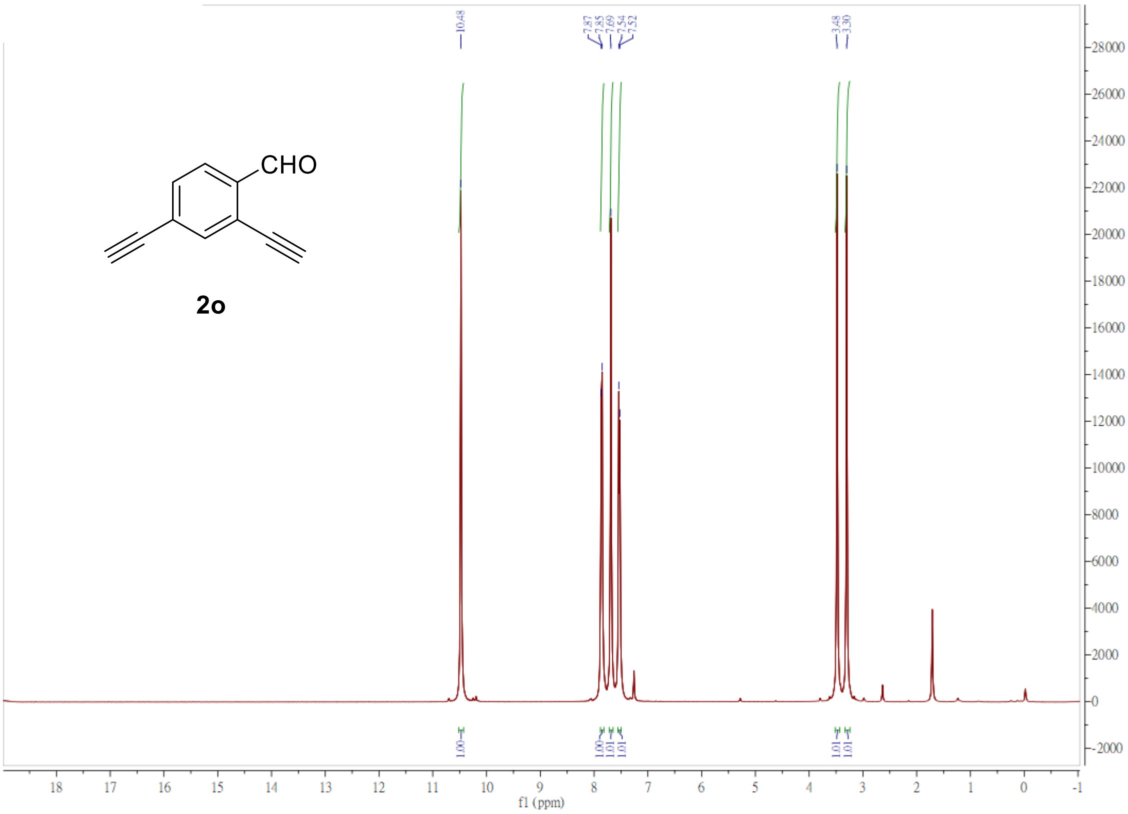

^{13}C NMR

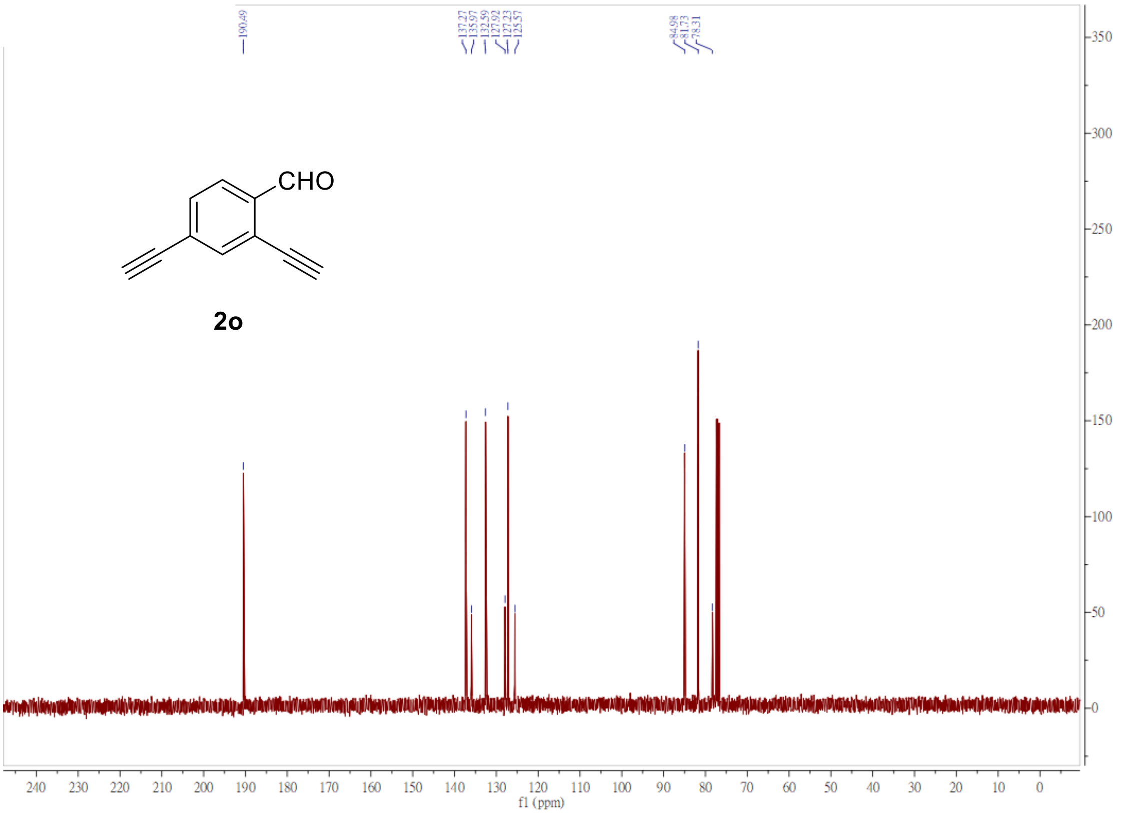

Supplementary Fig. 4 ^{1}H NMR and ^{13}C NMR spectra of compound **2o**.

^{1}H NMR

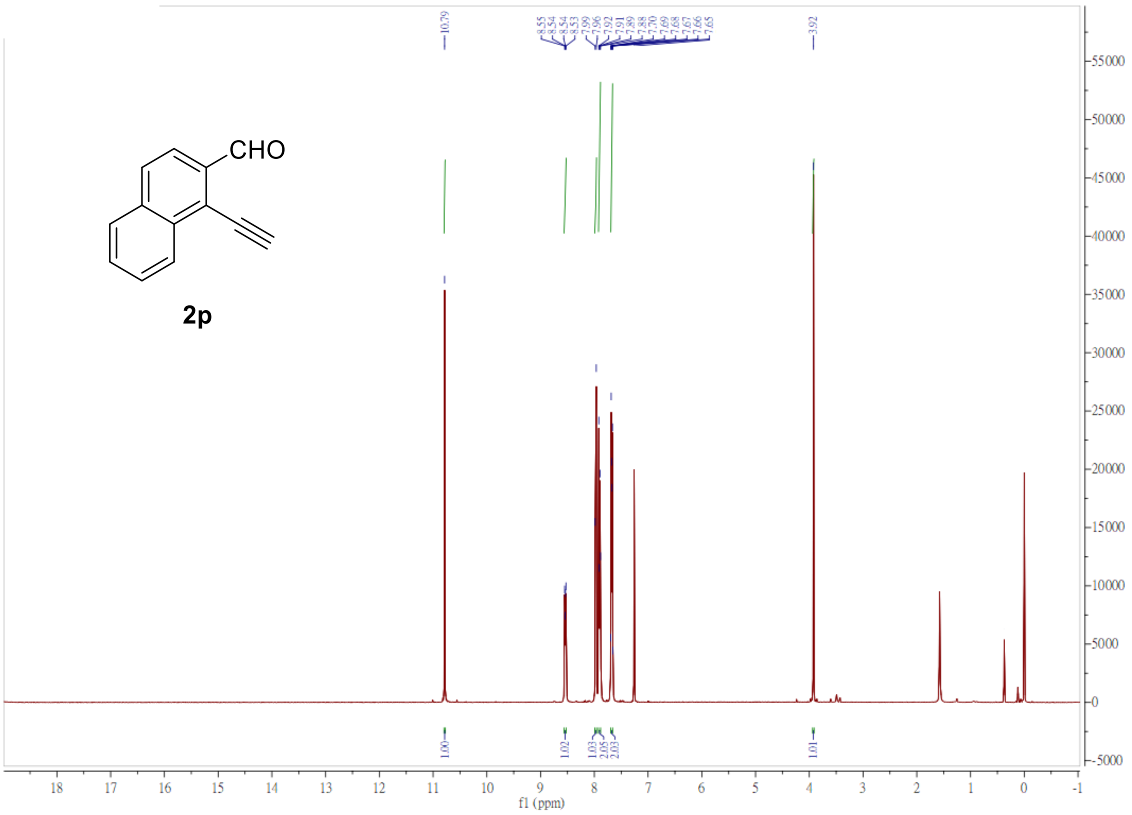

^{13}C NMR

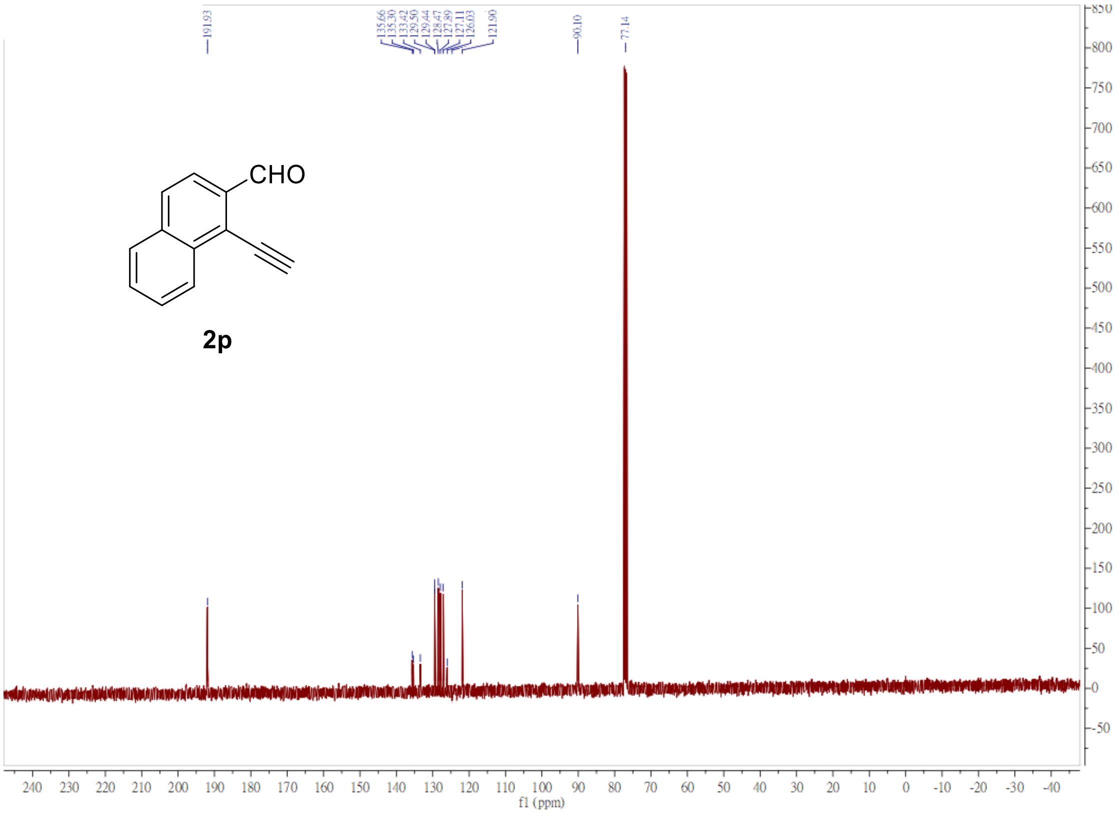

Supplementary Fig. 5 ^{1}H NMR and ^{13}C NMR spectra of compound **2p**.

Preparation of Isoquinolinium Product 3b

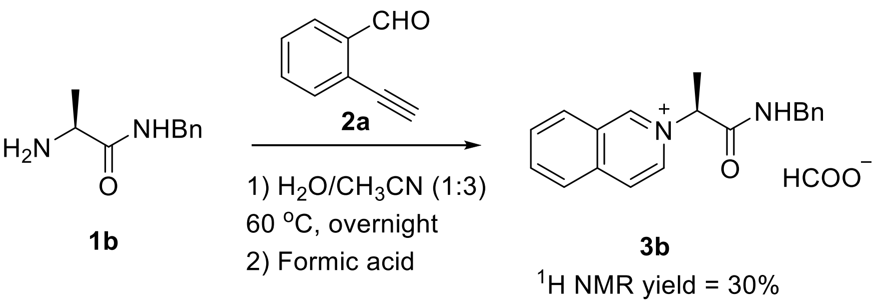

To a 25 mL round bottom flask with (*S*)-2-amino-*N*-benzylpropanamide **1b** (1 mmol, 178 mg) and 2-ethynylbenzaldehyde **2a** (1.1 mmol, 143 mg), a mixture of acetonitrile and distilled water (3:1, 20 mL) was added. The reaction mixture was heated at 60 °C overnight. Then, 2 equiv. of formic acid (2 mmol, 75.4 μL) was added to the mixture and stirred for 15 min. After that, the reaction mixture was concentrated under reduced pressure, and 10 mL of water was added. After filtration of the mixture with filter paper, the filtrate was concentrated under reduced pressure and the residue was purified by column chromatography over silica gel with dichloromethane/methanol (9:1) as the eluent to give the isoquinolinium product **3b**.

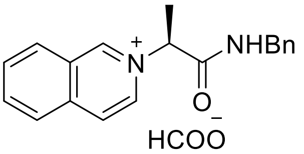

Isoquinolinium product 3b

White solid, 30% yield

^{1}H NMR (400 MHz, CD_3OD): δ 10.03 (s, 1H), 8.71 (d, *J* = 6.6 Hz, 1H), 8.55 (d, *J* = 8.3 Hz, 1H), 8.49 (d, *J* = 6.6 Hz, 1H), 8.38 (s, 1H), 8.31 (m, 2H), 8.10 (t, *J* = 7.4 Hz, 1H), 7.36 – 7.24 (m, 5H), 5.79 (d, *J* = 6.7 Hz, 1H), 4.45 (q, *J* = 14.8 Hz, 2H), 2.10 (d, *J* = 6.5 Hz, 3H). **^{13}C NMR** (100 MHz, CD_3OD): δ 167.61, 149.65, 138.15, 137.77, 137.46, 134.06, 131.24, 130.62, 128.30, 128.29, 128.28, 128.27, 127.63, 127.39, 127.17, 127.08, 125.55, 68.46, 43.41, 17.60. HRMS (ESI): calcd. for $C_{19}H_{19}N_2O^+$ (M - COOH)$^+$: 291.1498, found: 291.1493.

^{1}H NMR

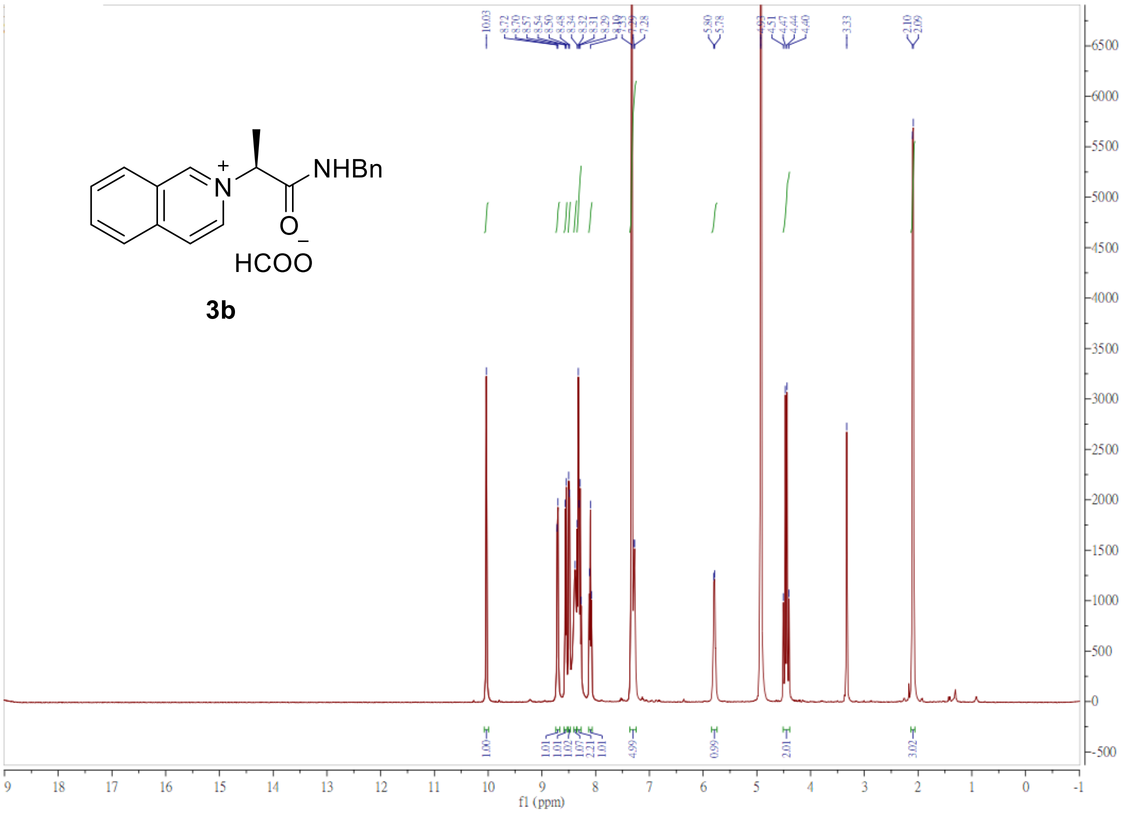

^{13}C NMR

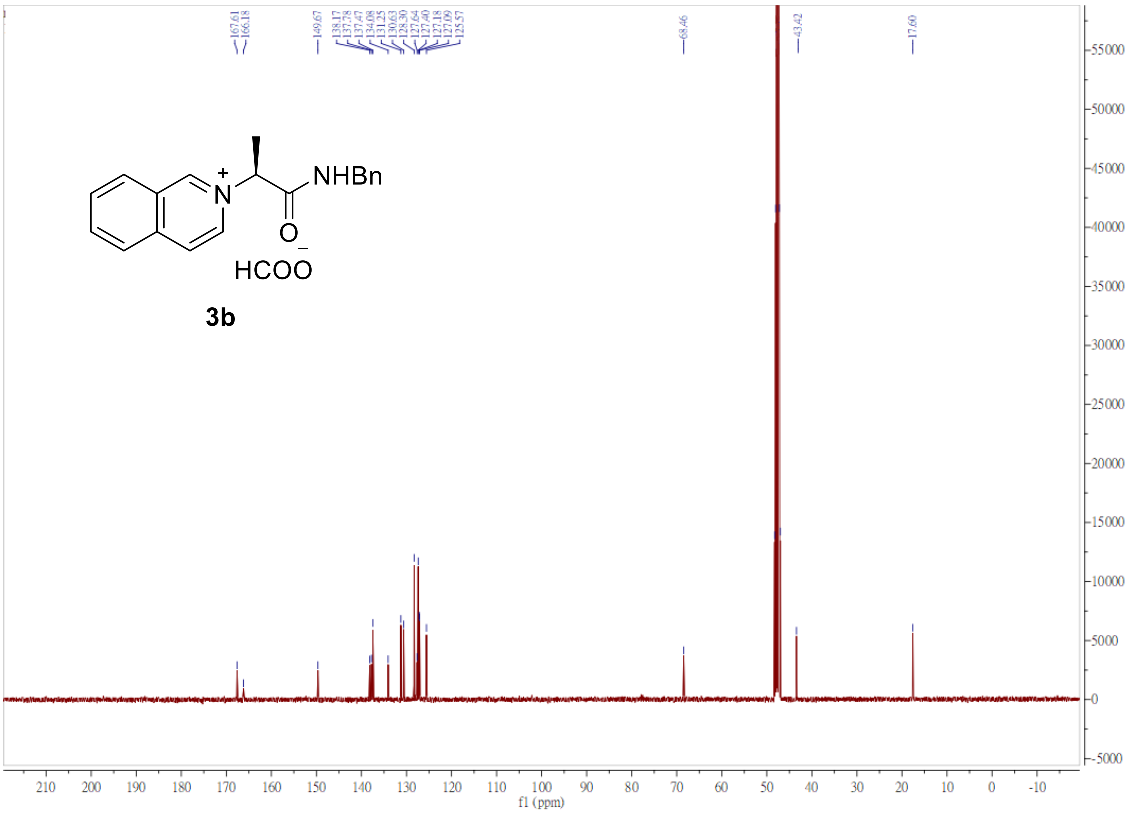

Supplementary Fig. 6 ^{1}H NMR and ^{13}C NMR spectra of compound **3b**.

Preparation of Alkyne-linked 2-Ethynylbenzaldehyde 2t

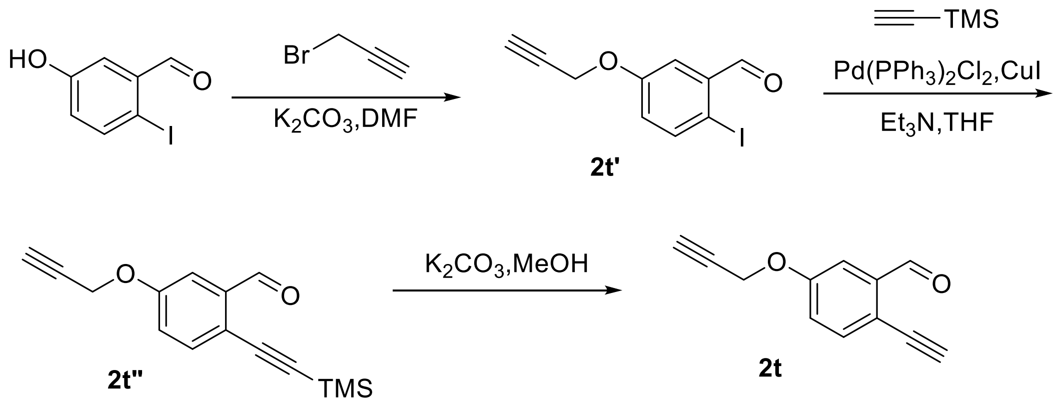

To a 25 mL round bottom flask of 5-hydroxy-2-iodobenzaldehyde (5 mmol, 1.24 mg) and potassium carbonate (15 mmol, 2.07 mg) with 15 mL *N*,*N*-dimethylformamide, propargyl bromide solution (~80% in toluene, 7.5 mmol, 1.5 mL) was added. The reaction mixture was stirred at room temperature overnight. After that, the reaction mixture was filtered and concentrated under reduced pressure. The residue was purified by column chromatography over silica gel with n-hexane/ethyl acetate (15.1) as the eluent to give 2-iodo-5-(prop-2-yn-1-yloxy)benzaldehyde (**2t'**).

To a Schlenk flask with bis(triphenylphosphine)palladium(II) dichloride (5 mol%) and copper iodide (2.5 mol%) under N_2 atmosphere, a solution of **2t'** (3.5 mmol) in tetrahydrofuran (10 mL) and trimethylamine (0.4 M) was added at room temperature, followed by trimethylsilylacetylene (4.2 mmol, 1.2 equiv.). The reaction mixture was stirred at room temperature for 1 h. Then, the mixture was filtered with celite. The filtrate was concentrated under reduced pressure and purified by column chromatography over silica gel with *n*-hexane/ethyl acetate (20:1) as the eluent to give **2t"**.

2t" was treated with potassium carbonate (0.50 g, 3.64 mmol) in methanol (30 mL) for 30 min at room temperature, and the solvent was removed under reduced pressure. The residue was extracted with dichloromethane and washed with saturated aqueous sodium carbonate, and dried over $MgSO_4$. The filtrate was concentrated under reduced pressure and purified by flash column chromatography over silica gel with *n*-hexane/ethyl acetate (20:1) to give propargyl-linked 2-ethynylbenzaldehyde **2t** as the product.

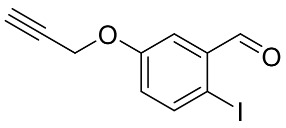

2-Iodo-5-(prop-2-yn-1-yloxy)benzaldehyde (2t')

White solid, 45% yield

^{1}H NMR (400 MHz, $CDCl_3$): δ 9.96 (s, 1H), 7.78 (d, *J* = 8.7 Hz, 1H), 7.45 (d, *J* = 3.2 Hz, 1H), 6.95 (dd, *J* = 8.7, 3.2 Hz, 1H), 4.70 (d, *J* = 2.3 Hz, 2H), 2.55 (t, *J* = 2.3 Hz, 1H). **^{13}C NMR** (100 MHz, $CDCl_3$): δ 195.31, 158.11, 141.18, 135.68, 123.84, 115.01, 90.77, 77.54, 76.56, 56.09. HRMS (ESI): calcd. for $C_{10}H_8IO_2^+$ $(M + H)^+$: 286.9563, found: 286.9570.

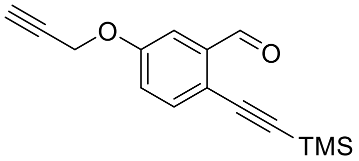

5-(prop-2-yn-1-yloxy)-2-((trimethylsilyl)ethynyl)benzaldehyde (2t")

White solid, 55% yield

^{1}H NMR (400 MHz, $CDCl_3$): δ 10.50 (s, 1H), 7.51 (d, *J* = 8.6 Hz, 1H), 7.46 (d, *J* = 2.8 Hz, 1H), 7.15 (dd, *J* = 8.6, 2.8 Hz, 1H), 4.75 (d, *J* = 2.4 Hz, 2H), 2.54 (t, *J* = 2.4 Hz, 1H), 0.26 (s, 9H). **^{13}C NMR** (100 MHz, $CDCl_3$): δ 191.48, 157.73, 137.57, 134.96, 121.99, 120.29, 110.96, 100.97, 99.91, 77.50, 76.33, 56.06, 30.91, -0.17. HRMS (ESI): calcd. for $C_{15}H_{17}O_2Si^+$ $(M + H)^+$: 257.0992, found:257.0994.

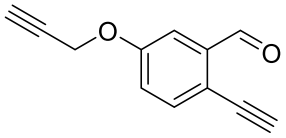

Alkyne-linked 2-ethynylbenzaldehyde (2t)

White solid, 80% yield

^{1}H NMR (400 MHz, $CDCl_3$): δ 10.50 (s, 1H), 7.56 (d, *J* = 8.5 Hz, 1H), 7.49 (d, *J* = 2.8 Hz, 1H), 7.18 (dd, *J* = 8.5, 2.8 Hz, 1H), 4.76 (d, *J* = 2.4 Hz, 2H), 3.38 (s, 1H), 2.55 (s, 1H). **^{13}C NMR** (100 MHz, $CDCl_3$): δ 190.95, 157.94, 137.92, 135.28, 121.81, 118.82, 111.38, 83.19, 79.01, 77.52, 76.50, 56.04. HRMS (ESI): calcd. for $C_{12}H_9O_2^+$ $(M + H)^+$: 185.0597, found:185.0594.

^{1}H NMR

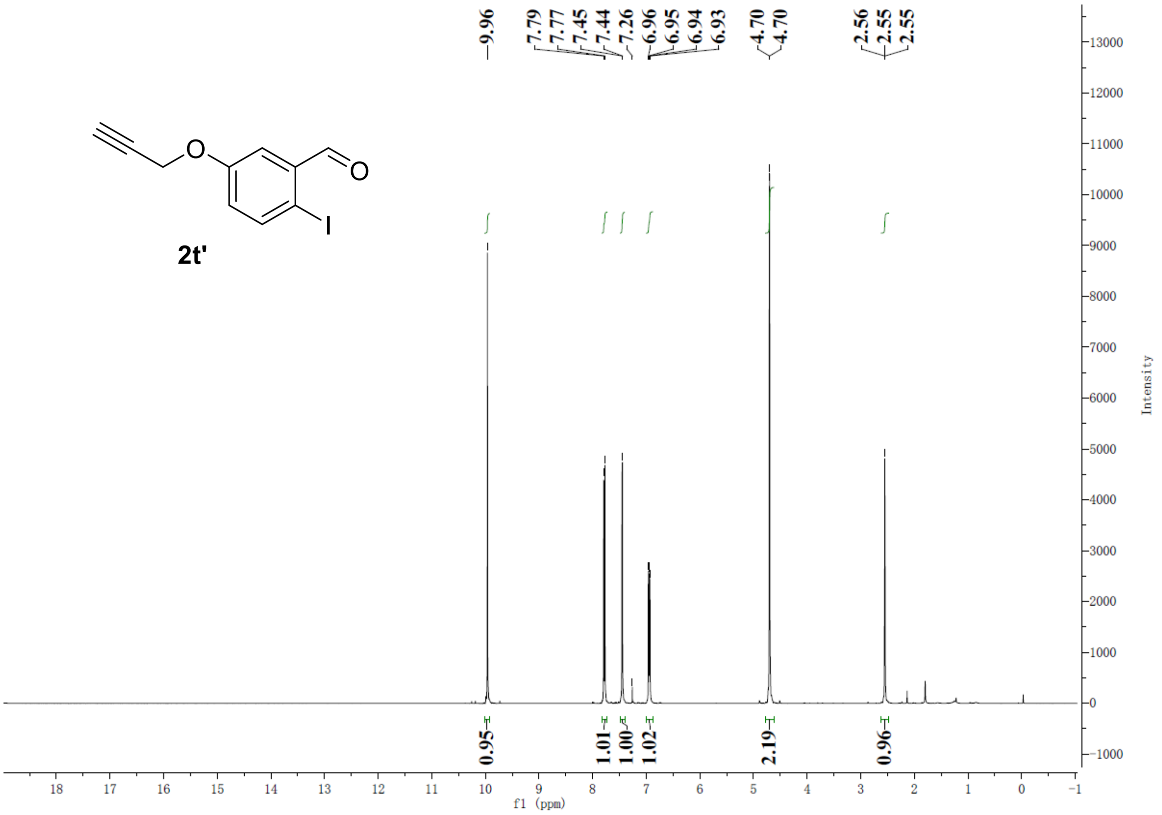

^{13}C NMR

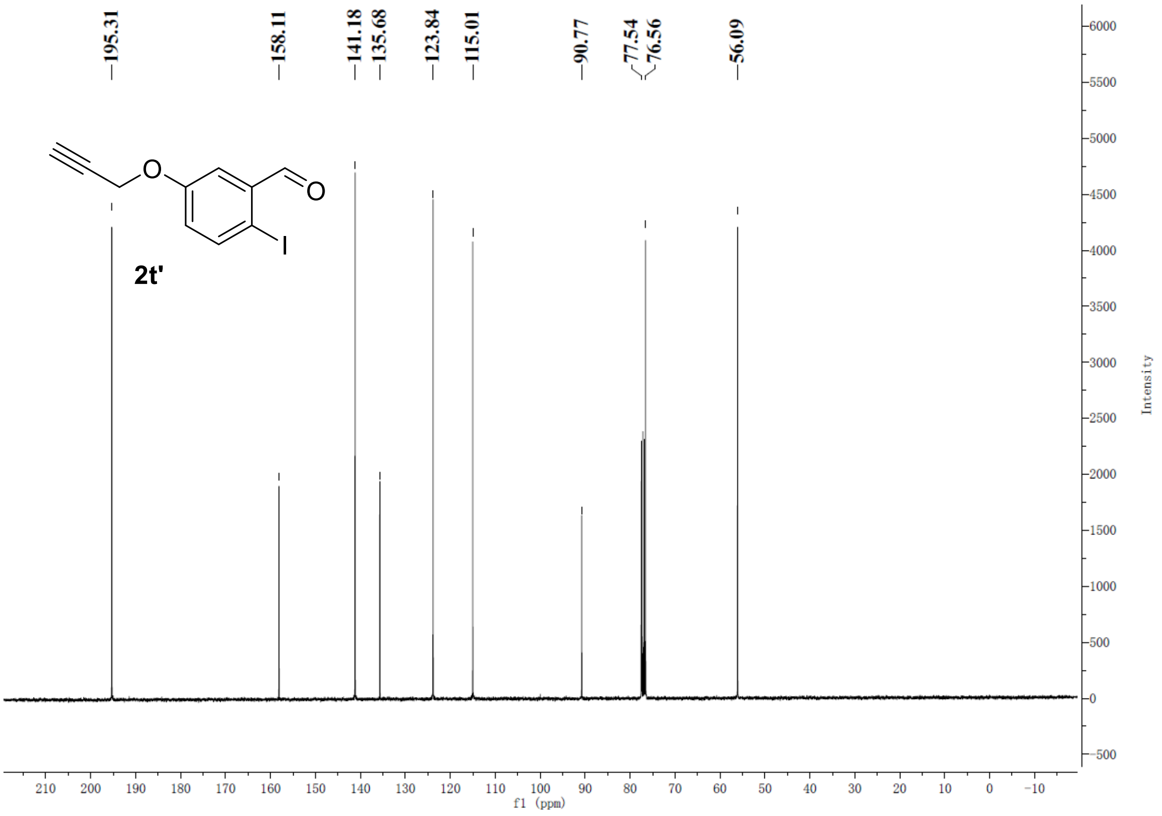

Supplementary Fig. 7 ^{1}H NMR and ^{13}C NMR spectra of compound **2t'**.

^{1}H NMR

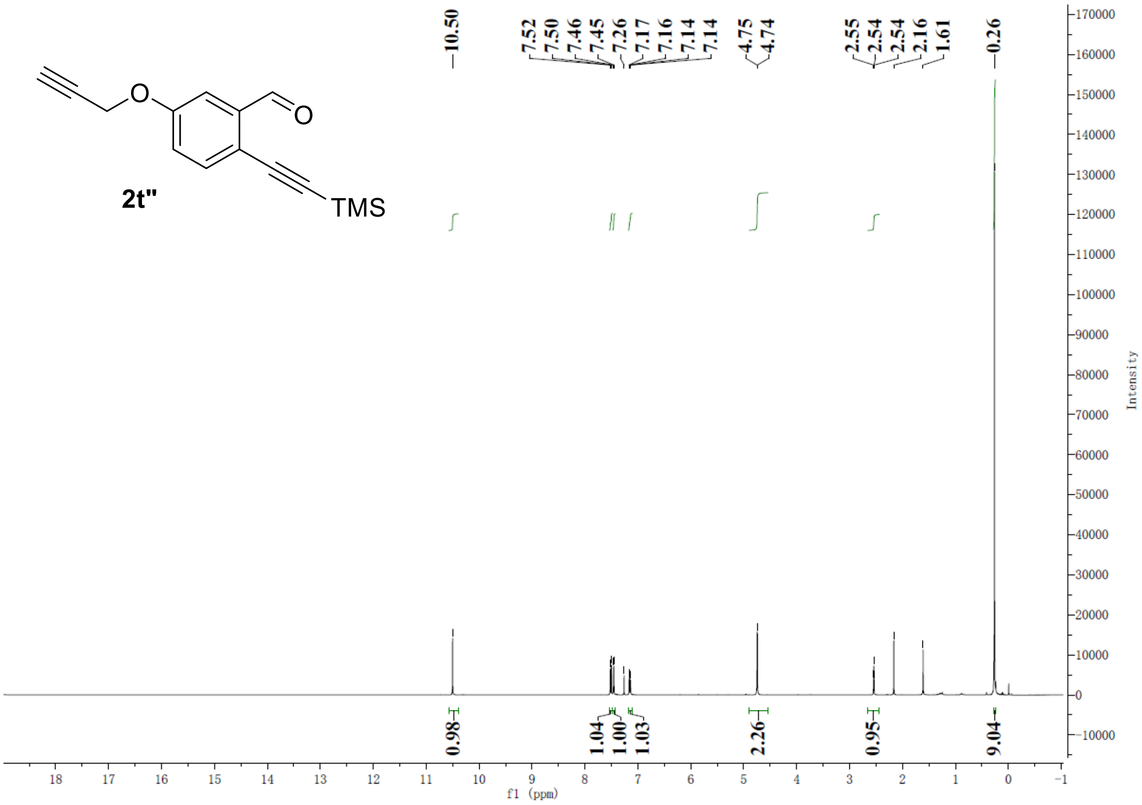

^{13}C NMR

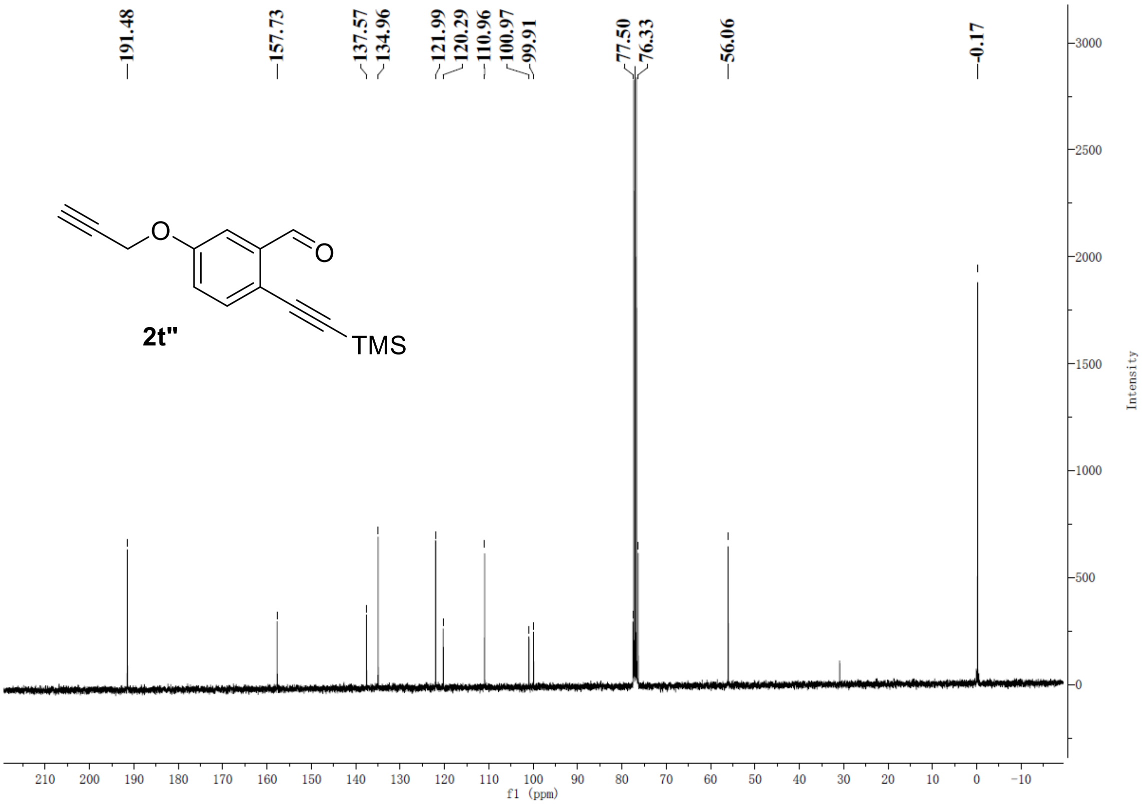

Supplementary Fig. 8 ^{1}H NMR and ^{13}C NMR spectra of compound **2t”**.

^{1}H NMR

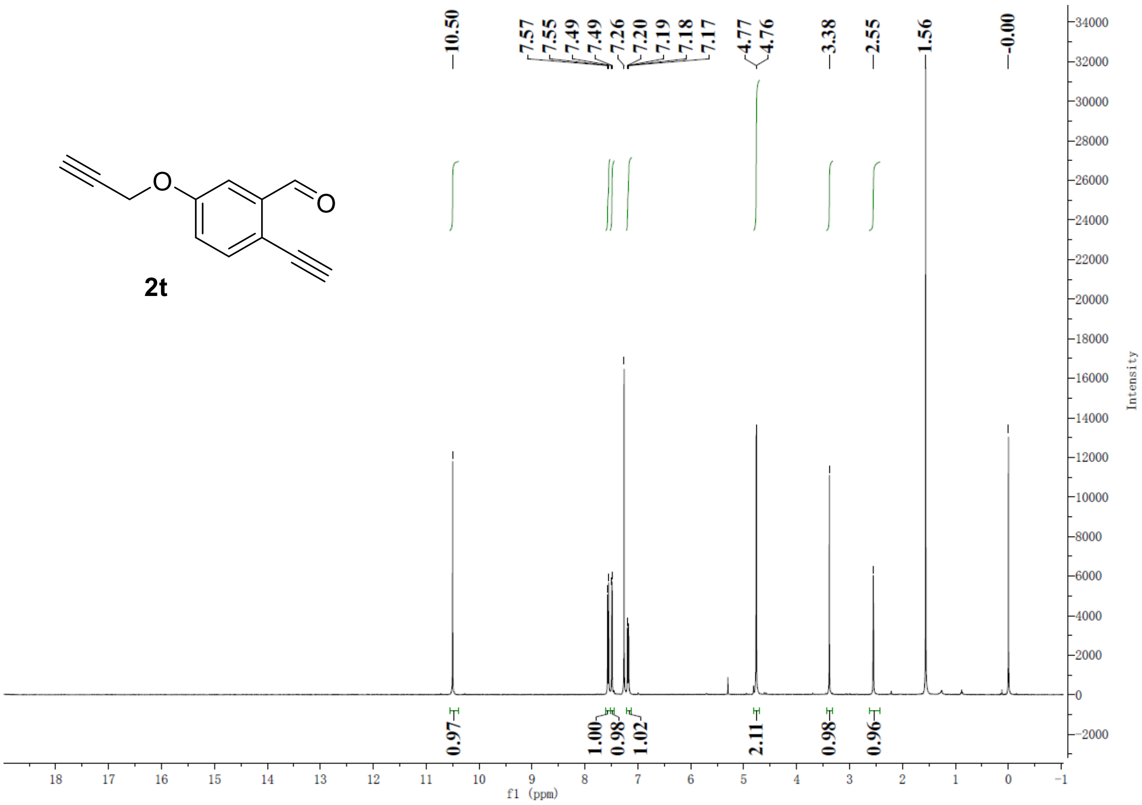

^{13}C NMR

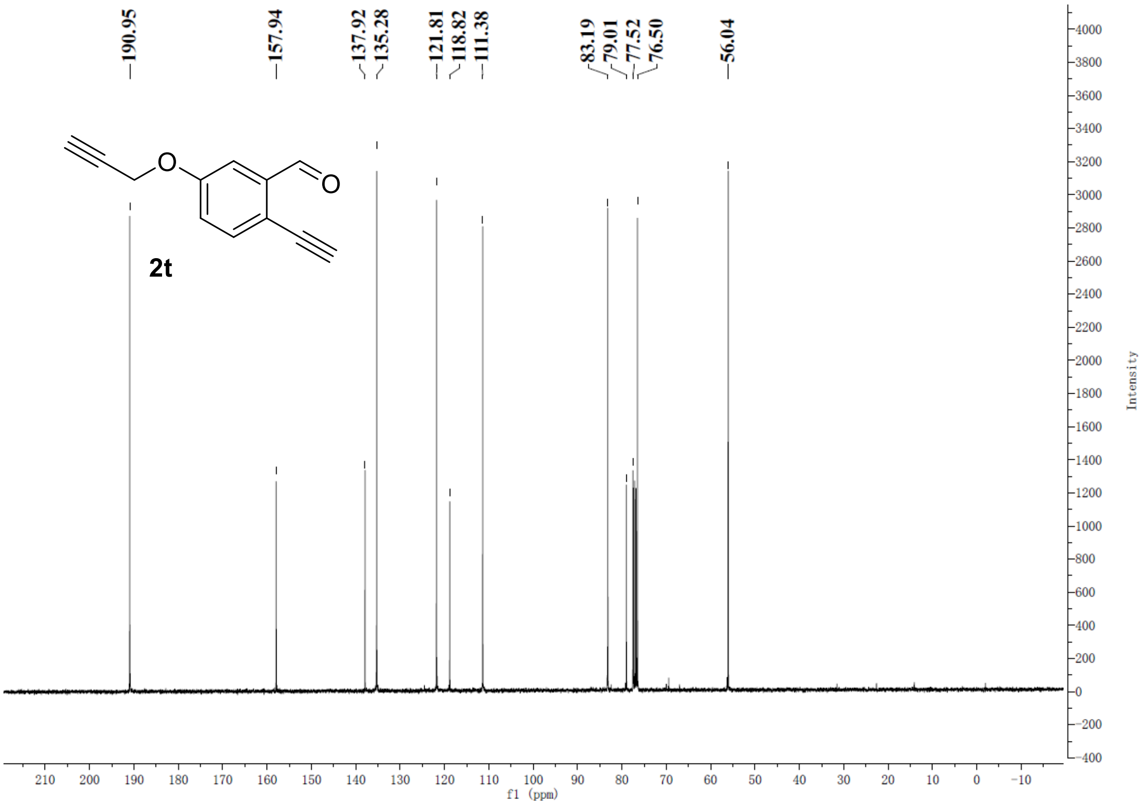

Supplementary Fig. 9 ^{1}H NMR and ^{13}C NMR spectra of compound **2t**.

Preparation of Fluorescein-linked 2-Ethynylbenzaldehyde 2u

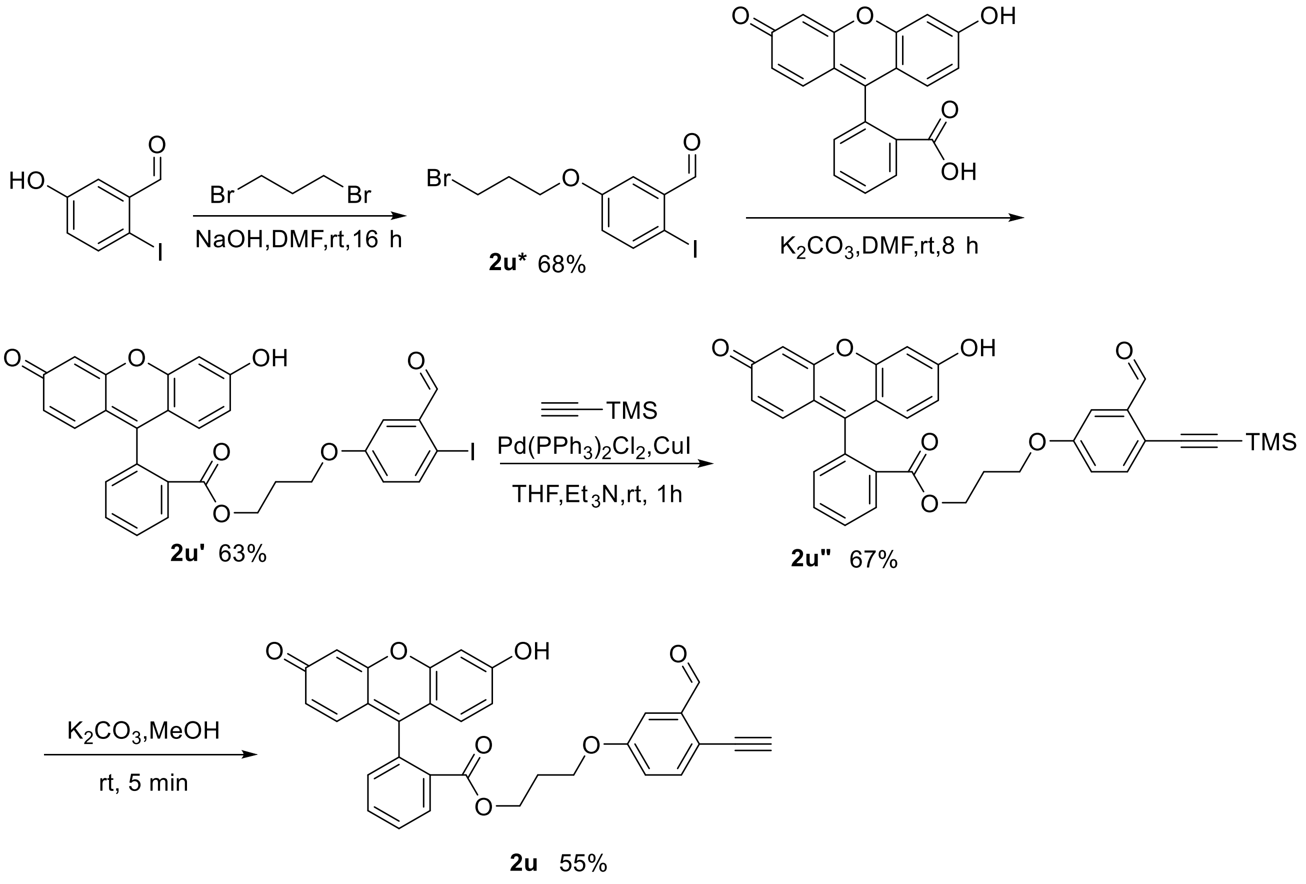

To a 25 mL round bottom flask of 5-hydroxy-2-iodobenzaldehyde (5 mmol, 1.24 g) and sodium hydroxide (5 mmol, 200 mg) with 15 mL *N*,*N*-dimethylformamide, 1,3-dibromopropane (11 mmol, 1.12 mL) was added. The reaction mixture was stirred at room temperature for 16 h. After that, the reaction mixture was filtered and concentrated under reduced pressure. The residue was purified by column chromatography over silica gel with n-hexane/ethyl acetate (15:1) as the eluent to give 5-(3-bromopropoxy)-2-iodobenzaldehyde (**2u***).

To a 25 mL round bottom flask of **2u*** (2 mmol, 738 mg) and potassium carbonate (6 mmol, 828 mg) with 10 mL *N*,*N*-dimethylformamide, fluorescein (2.4 mmol, 797 mL) was added. The reaction mixture was stirred at room temperature for 16 h. After that, the reaction mixture was filtered and concentrated under reduced pressure. The residue was purified by column chromatography over silica gel with dichoromethane/methanol (10:1) as the eluent to give compound **2u'**.

To a Schlenk flask with bis(triphenylphosphine)palladium(II) dichloride (5 mol%) and copper iodide (2.5 mol%) under N_2 atmosphere, a solution of **2u'** (1.5 mmol) in tetrahydrofuran (10 mL) and trimethylamine (0.4 M) was added at room temperature, followed by trimethylsilylacetylene (1.8 mmol, 1.2 equiv.). The reaction mixture was stirred at room temperature for 1 h. Then, the mixture was filtered with celite. The filtrate was concentrated under reduced pressure and purified by column chromatography over silica gel with *n*-hexane/ethyl acetate (15:1) as the eluent to give **2u"**.

2u" was treated with potassium carbonate (414 mg, 3 mmol) in methanol (10 mL) for 30 min at room temperature, and the solvent was removed under reduced pressure. The residue was extracted with dichloromethane and washed with saturated aqueous sodium carbonate, and dried over $MgSO_4$. The filtrate was concentrated under reduced pressure and purified by flash column chromatography over silica gel with dichoromethane/methanol (10:1) to give propargyl-linked 2-ethynylbenzaldehyde **2u** as the product.

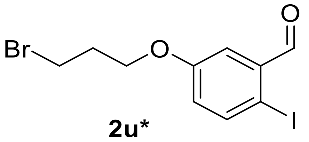

2u*

5-(3-bromopropoxy)-2-iodobenzaldehyde (2u*)

White solid, 68% yield

^{1}H NMR (400 MHz, $CDCl_3$): δ 9.96 (d, *J* = 4.5 Hz, 1H), 8.11 – 7.92 (m, 1H), 7.35 – 7.26 (m, 1H), 7.22 (dd, *J* = 7.8, 1.6 Hz, 1H), 4.27 (t, *J* = 6.0 Hz, 2H), 3.73 (t, *J* = 6.0 Hz, 2H), 2.50 – 2.35 (m, 2H).

HRMS (ESI): calcd. for $C_{10}H_{11}BrIO_2^+$ $(M + H)^+$: 368.8982, found:368.8980.

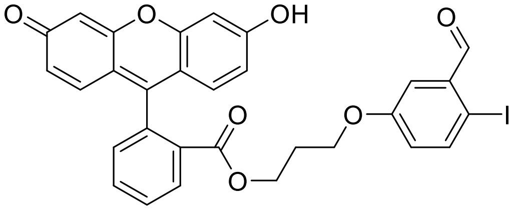

2u'

Orange solid, 63% yield

^{1}H NMR (400 MHz, $CDCl_3$): δ 9.89 (s, 1H), 9.64 (s, 1H), 8.24 (d, *J* = 6.9 Hz, 1H), 7.75 – 7.65 (m, 3H), 7.29 (d, *J* = 6.9 Hz, 1H), 7.20 (d, *J* = 3.1 Hz, 1H), 6.97 (d, *J* = 9.0 Hz, 2H), 6.86 – 6.77 (m, 4H), 6.72 (dd, *J* = 9.0, 3.1 Hz, 1H), 4.12 (t, *J* = 6.0 Hz, 2H), 3.67 (t, *J* = 6.0 Hz, 2H), 1.83 – 1.71 (m, 2H). HRMS (ESI): calcd. for $C_{30}H_{22}IO_7^+$ $(M + H)^+$: 621.0405, found:621.0409.

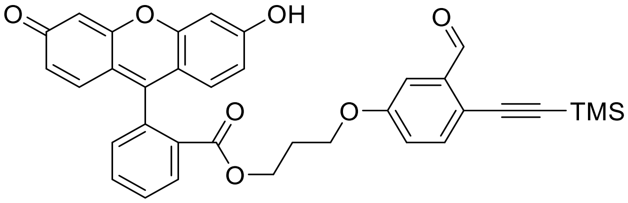

2u''

Orange solid, 67% yield

^{1}H NMR (400 MHz, $CDCl_3$): δ 10.46 (s, 1H), 8.24 (d, *J* = 7.3 Hz, 1H), 7.70 (dt, *J* = 23.2, 7.1 Hz, 2H), 7.46 (d, *J* = 8.5 Hz, 1H), 7.39 (s, 2H), 7.30 (d, *J* = 7.3 Hz, 1H), 7.20 (d, *J* = 2.6 Hz, 1H), 6.95 (d, *J* = 9.2 Hz, 3H), 6.84 (s, 2H), 6.79 (d, *J* = 9.1 Hz, 2H), 4.15 (s, 2H), 3.73 (s, 2H), 1.82 (d, *J* = 5.9 Hz, 2H), 0.24 (s, 9H). HRMS (ESI): calcd. for $C_{35}H_{31}O_7Si^+$ $(M + H)^+$: 591.1834, found:591.1837.

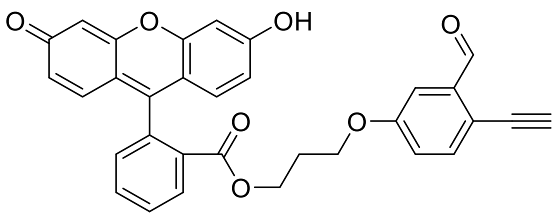

2u

Orange solid, 55% yield

^{1}H NMR (400 MHz, $CDCl_3$) δ 10.40 (s, 1H), 9.08 (s, 2H), 8.24 (d, *J* = 7.0 Hz, 1H), 7.70 (m, 3H), 7.61 (d, *J* = 8.6 Hz, 1H), 7.46 (d, *J* = 8.6 Hz, 1H), 7.34 – 7.23 (m, 2H), 7.20 (d, *J* = 2.7 Hz, 1H), 6.97 (d, *J* = 9.2 Hz, 2H), 6.88 – 6.83 (m, 2H), 6.80 (dd, *J* = 9.2, 1.7 Hz, 3H), 4.14 (t, *J* = 5.9 Hz, 2H), 3.71 (t, *J* = 5.9 Hz, 2H), 3.33 (s, 1H), 1.79 (dd, *J* = 11.8, 5.9 Hz, 2H). HRMS (ESI): calcd. for $C_{32}H_{23}O_7^+$ (M + H)$^+$: 519.1438, found: 519.1441.

^{1}H NMR

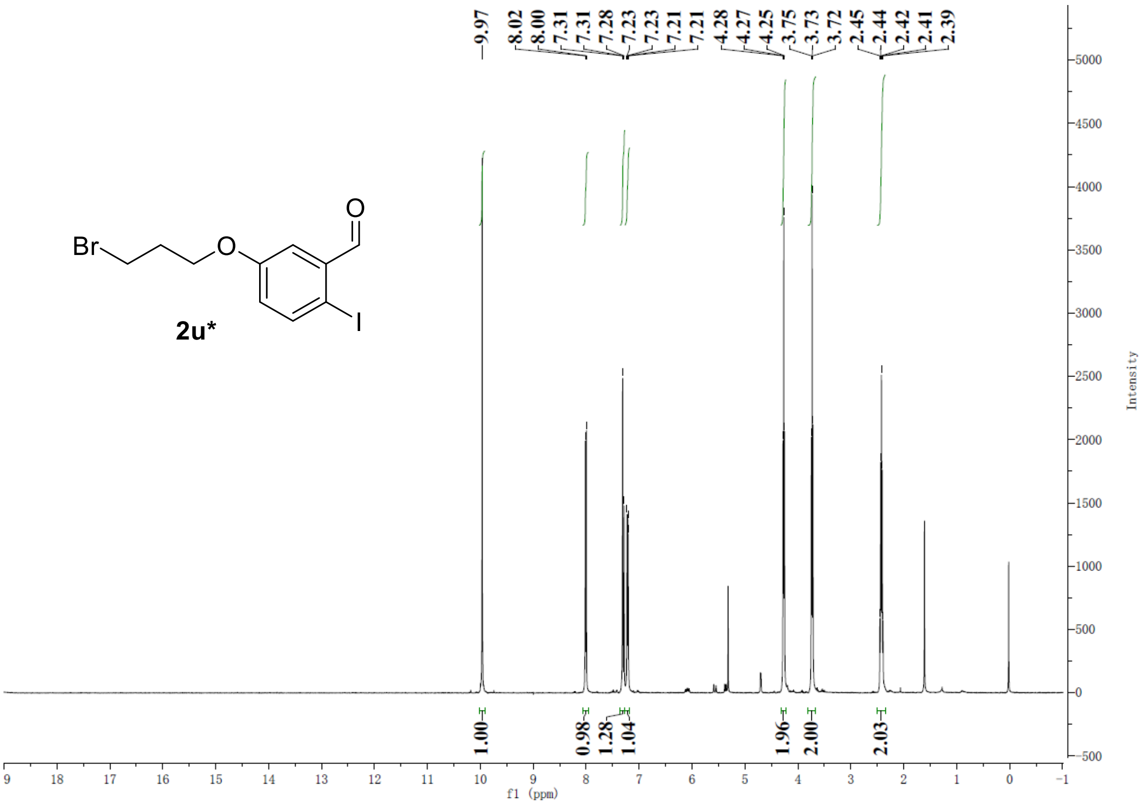

^{1}H NMR

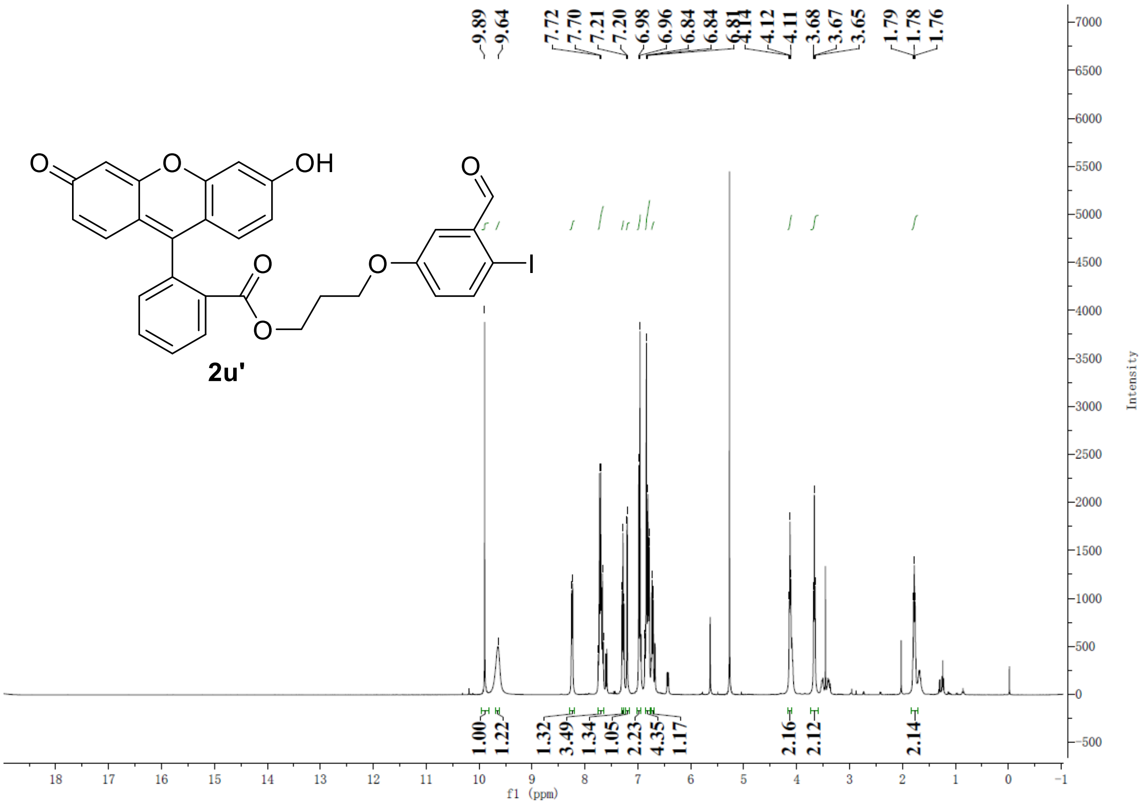

Supplementary Fig. 10 ^{1}H NMR and ^{13}C NMR spectra of compound **2u’**.

¹H NMR

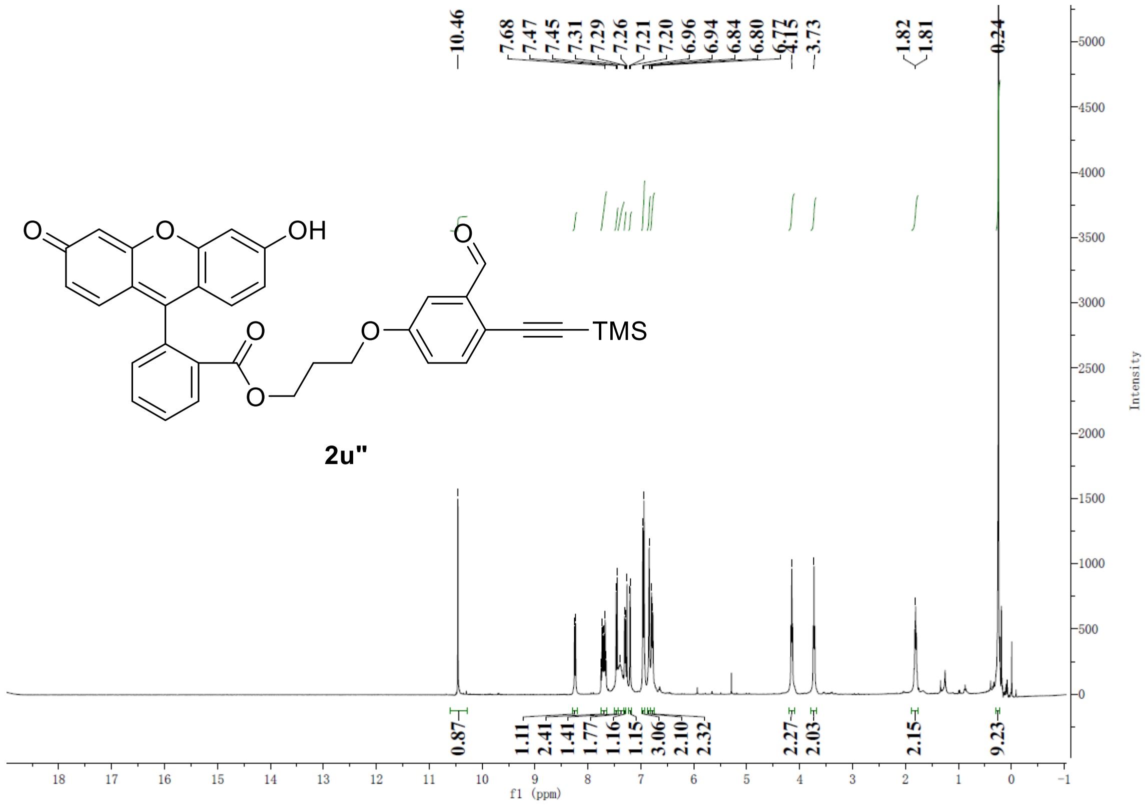

¹H NMR

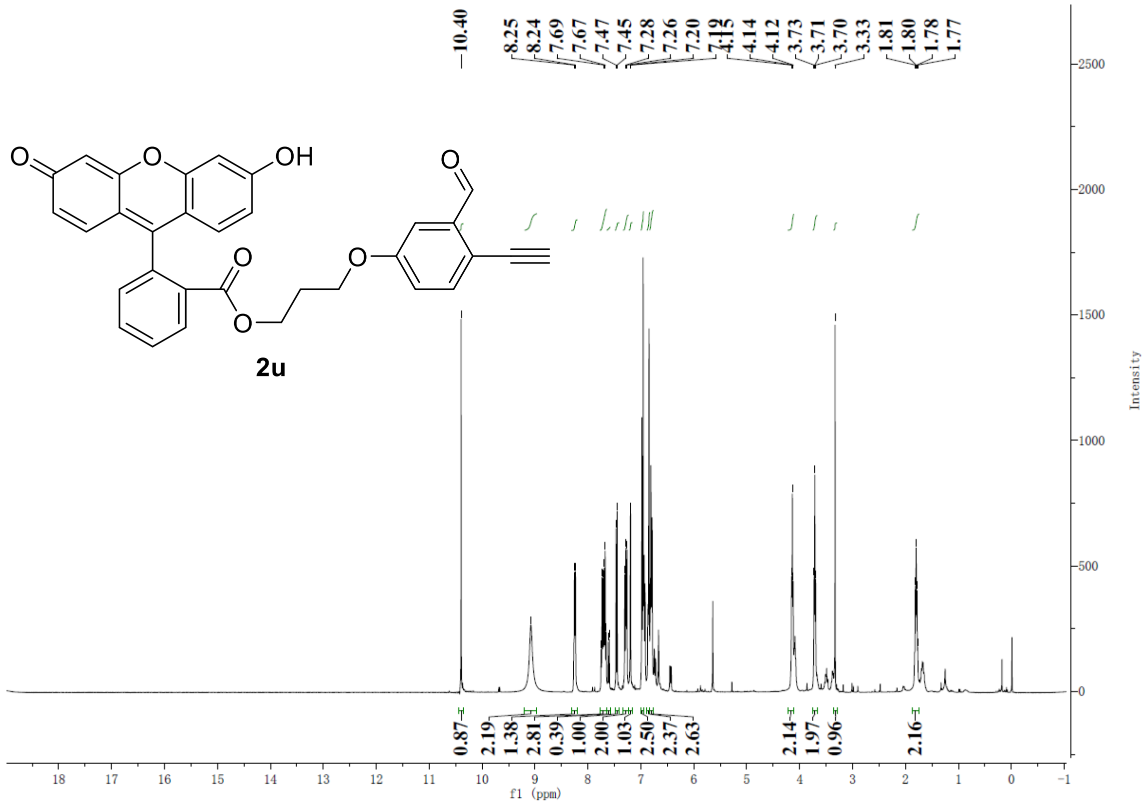

Supplementary Fig. 11 ¹H NMR and ¹³C NMR spectra of compound **2u**.

General Procedure for Modification of Peptides using 2-Ethynylbenzaldehydes

To an eppendorf tube (1.5 mL) with 80 µL of 50 mM PBS buffer pH 6.5, 10 µL of YTSSSKNVR (**1a**, 1 mM in Milli-Q® water) was added to the buffer, followed by 10 µL of 2-ethynylbenzaldehyde (**2a-2t**, 20 mM in DMSO). The reactive mixture was allowed to react in a 37 ^{o}C water bath for 16 h. 10 µL of the mixture was drawn, diluted with 10 µL Milli-Q® and subjected to LC/MS-MS analysis.

Unless otherwise specified, all peptides were treated as same as the above procedure.

MS/MS Analysis of YTSSSKNVVR (1a) with 2-Ethynylbenzaldehydes

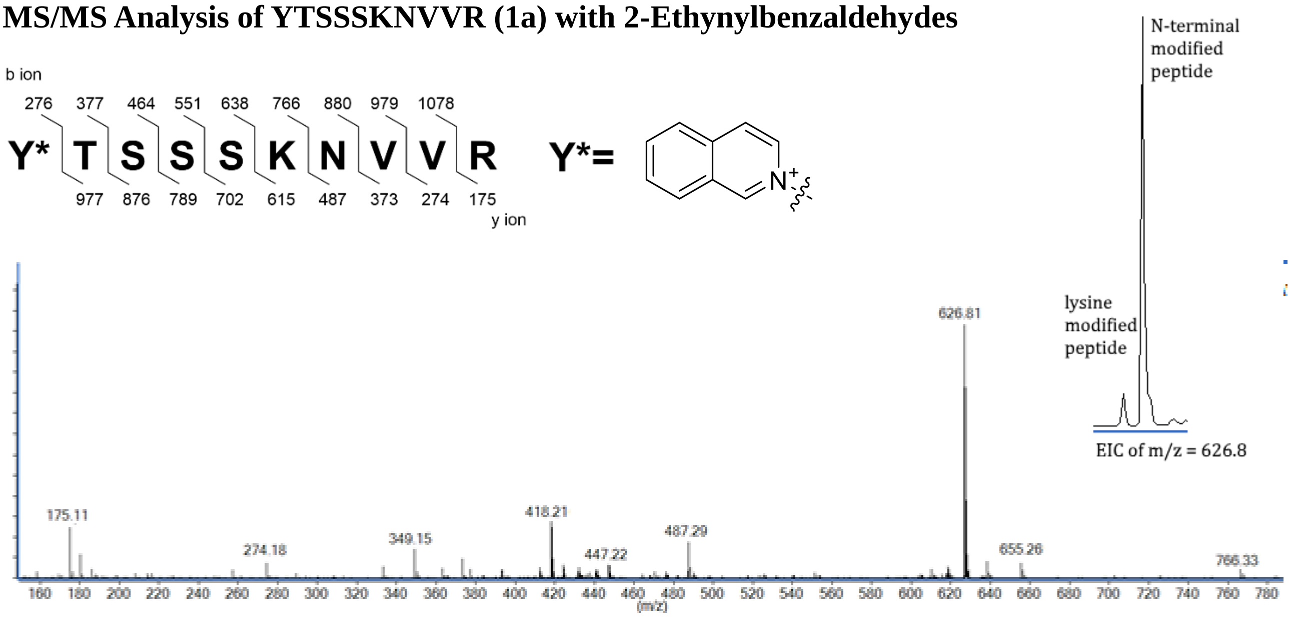

Supplementary Fig. 12 Q-TOF MS/MS spectrum of N-terminal **2a**-modified YTSSSKNVVR (ESI source, doubly charged ion of *m/z* = 626.8). Inset: the extracted ion chromatogram (EIC) of doubly charged ion of *m/z* = 626.8.

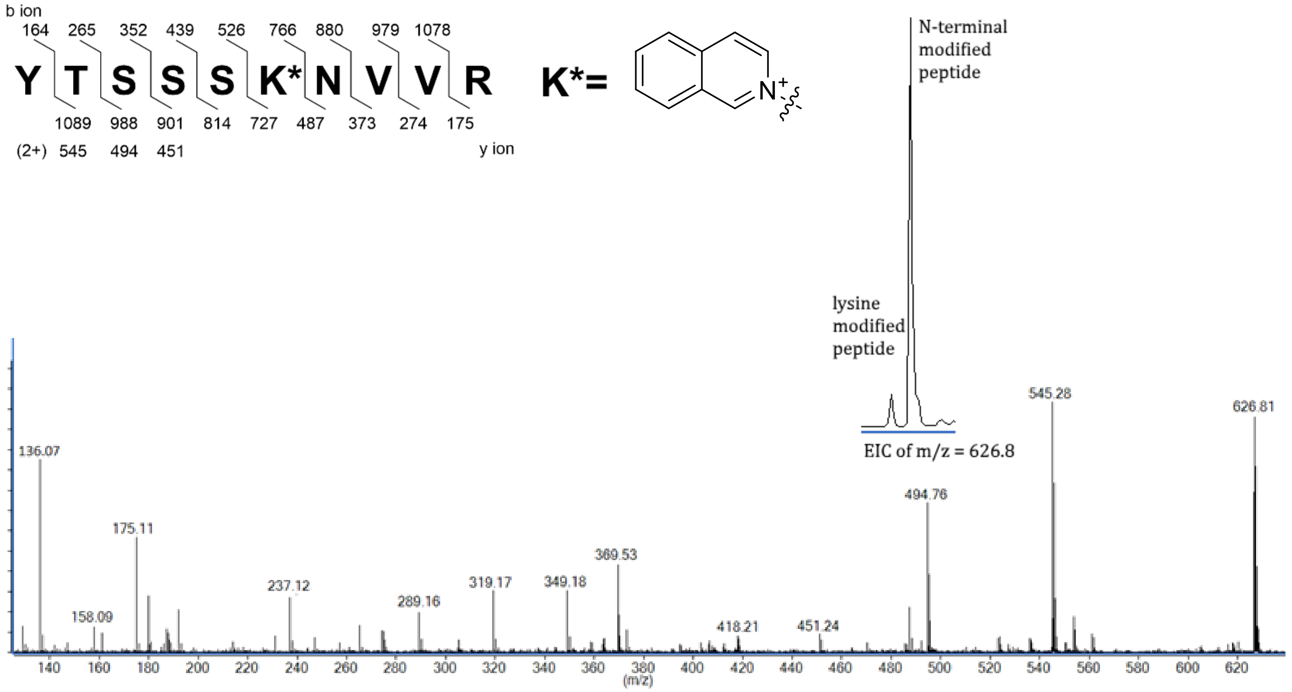

Supplementary Fig. 13 Q-TOF MS/MS spectrum of lysine **2a**-modified YTSSSKNVVR (ESI source, doubly charged ion of m/z = 626.8). Inset: the extracted ion chromatogram (EIC) of doubly charged ion of m/z = 626.8.

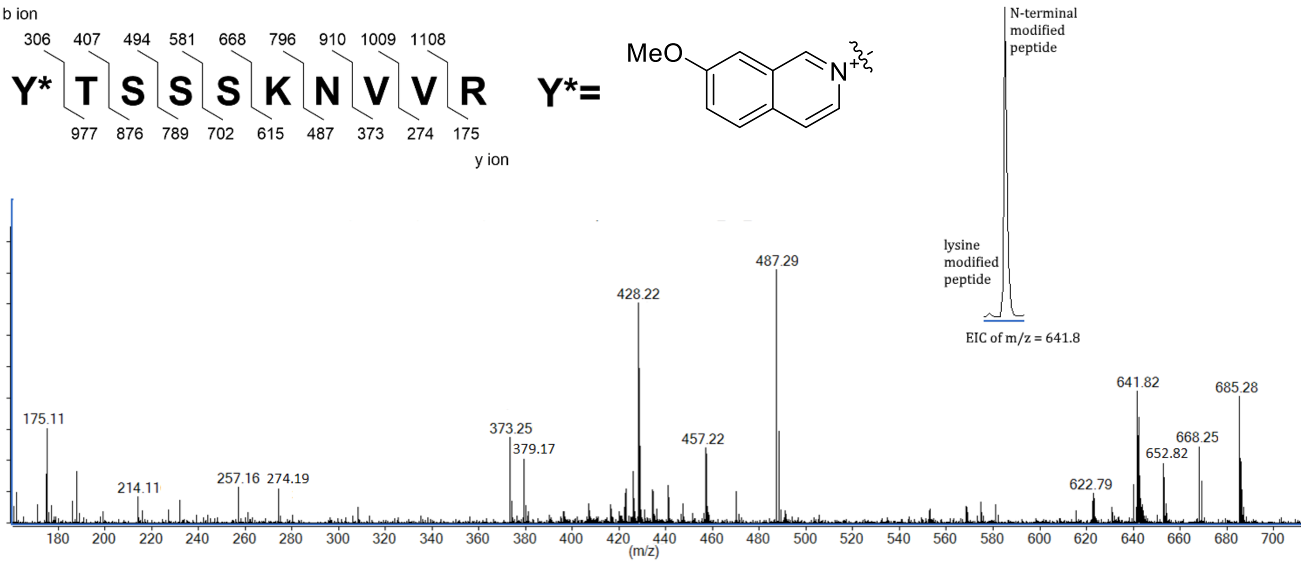

Supplementary Fig. 14 Q-TOF MS/MS spectrum of N-terminal **2b**-modified YTSSSKNVVR (ESI source, doubly charged ion of *m/z* = 641.8). Inset: the extracted ion chromatogram (EIC) of doubly charged ion of *m/z* = 641.8.

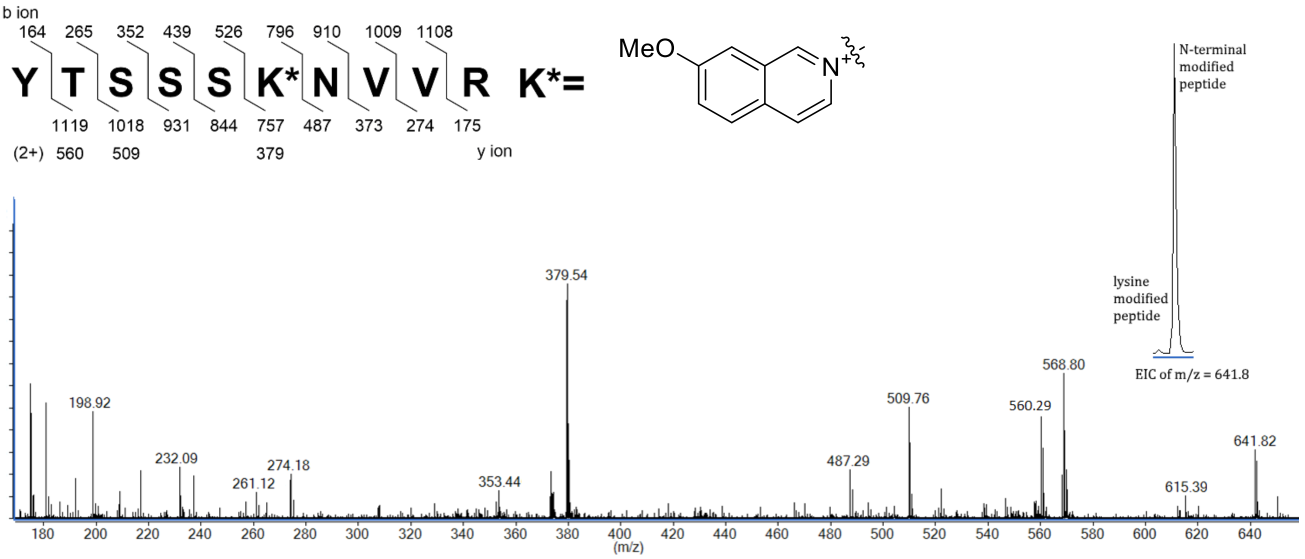

Supplementary Fig. 15 Q-TOF MS/MS spectrum of lysine **2b**-modified YTSSSKNVVR (ESI source, doubly charged ion of *m/z* = 641.8). Inset: the extracted ion chromatogram (EIC) of doubly charged ion of *m/z* = 641.8.

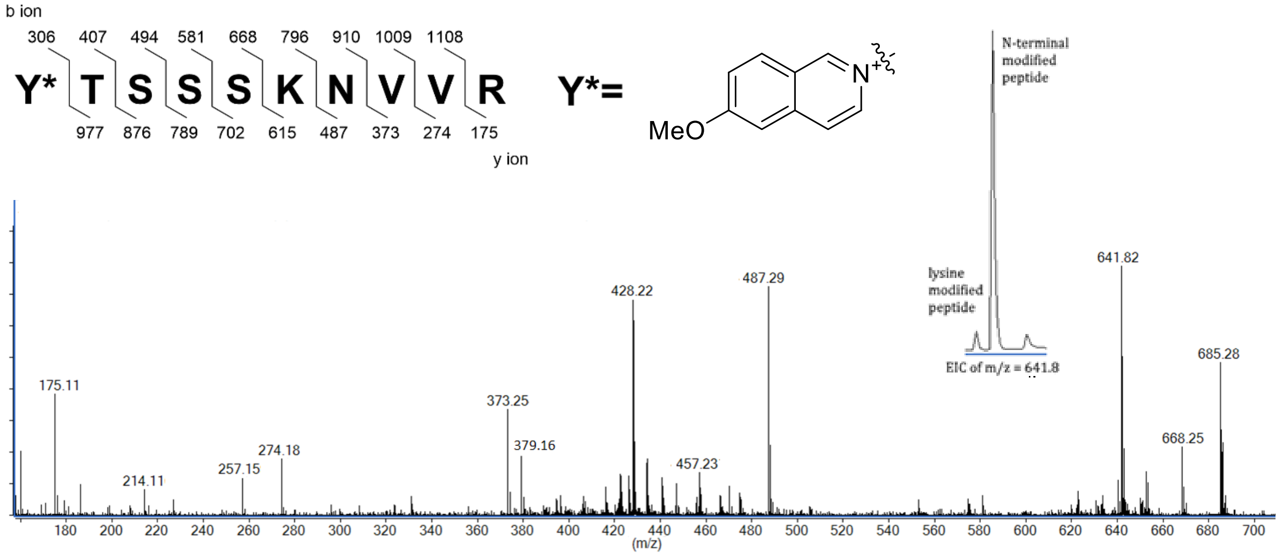

Supplementary Fig. 16 Q-TOF MS/MS spectrum of N-terminal **2c**-modified YTSSSKNVVR (ESI source, doubly charged ion of *m/z* = 641.8). Inset: the extracted ion chromatogram (EIC) of doubly charged ion of *m/z* = 641.8.

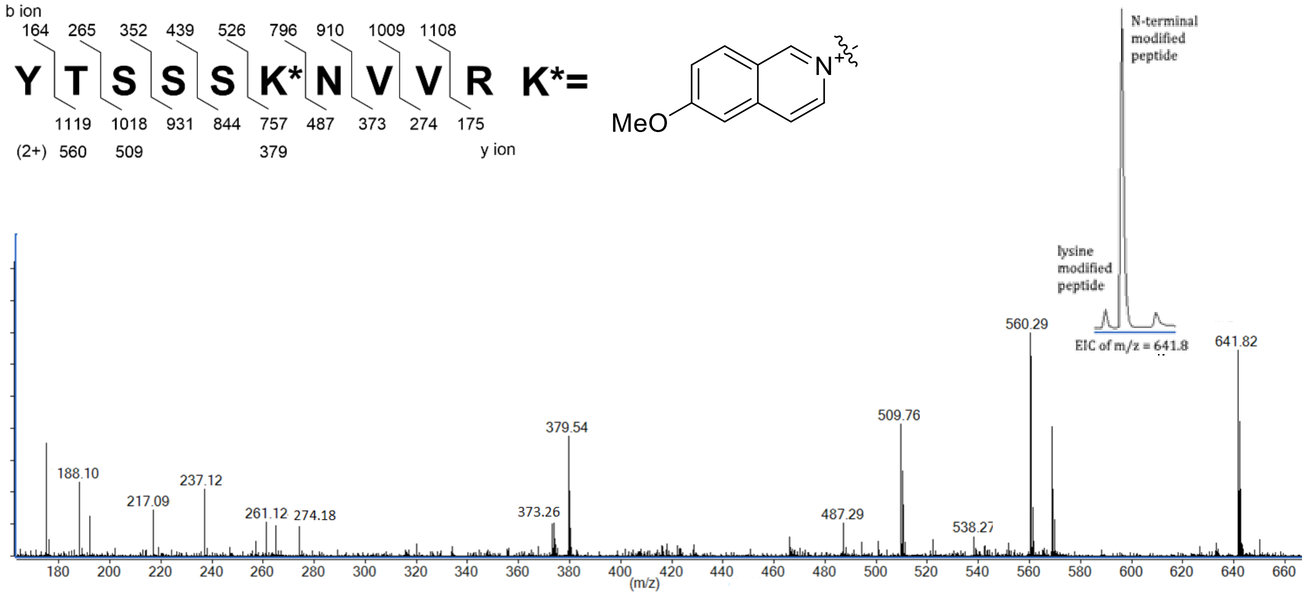

Supplementary Fig. 17 Q-TOF MS/MS spectrum of lysine **2c**-modified YTSSSKNVVR (ESI source, doubly charged ion of *m/z* = 641.8). Inset: the extracted ion chromatogram (EIC) of doubly charged ion of *m/z* = 641.8.

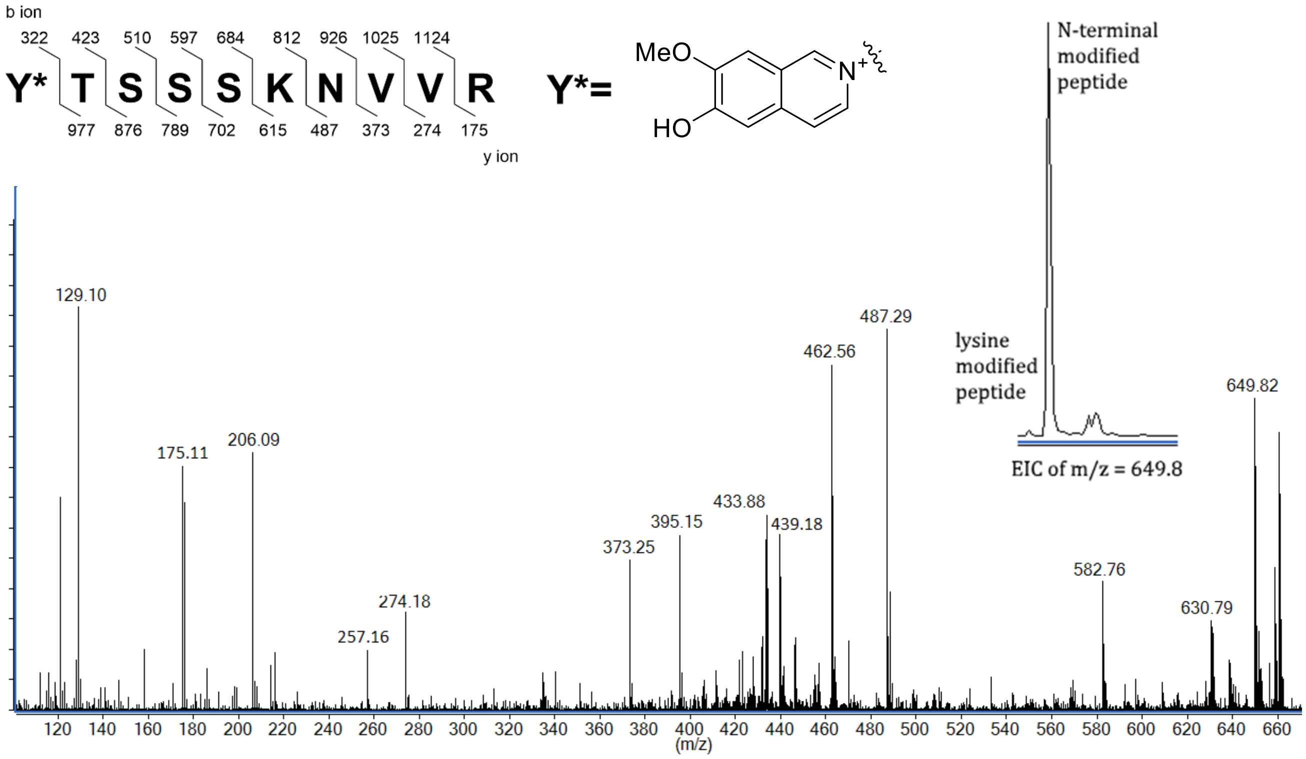

Supplementary Fig. 18 Q-TOF MS/MS spectrum of N-terminal **2d**-modified YTSSSKNVVR (ESI source, doubly charged ion of *m/z* = 649.8). Inset: the extracted ion chromatogram (EIC) of doubly charged ion of *m/z* = 649.8.

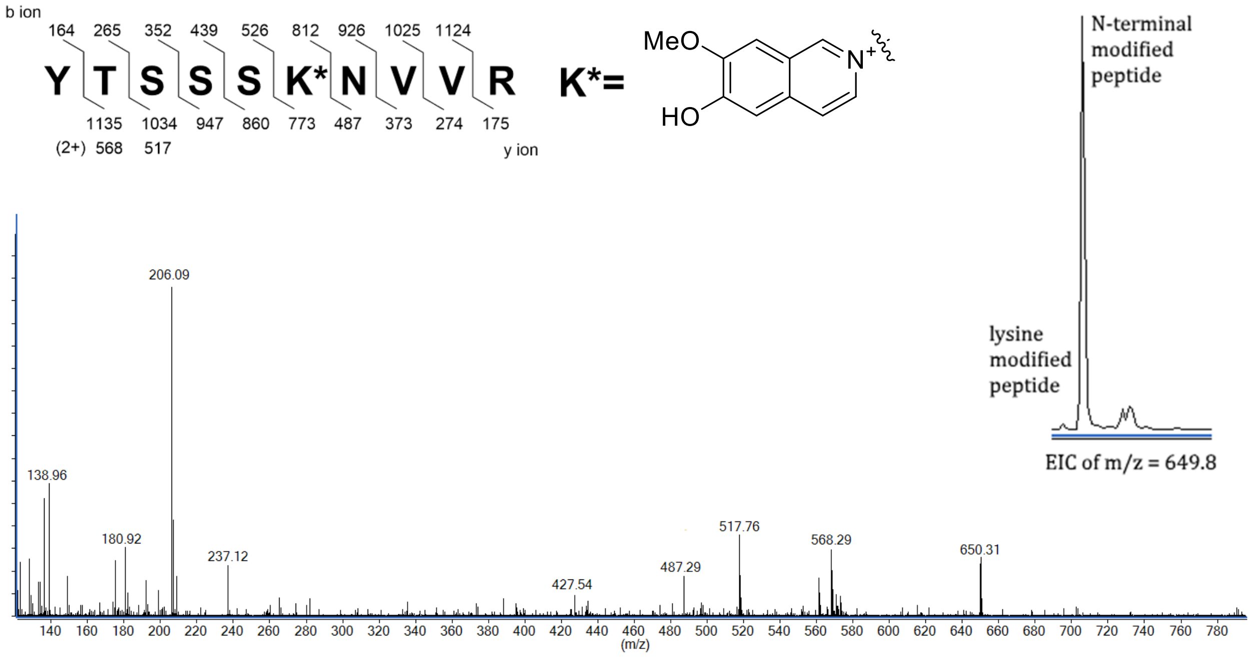

Supplementary Fig. 19 Q-TOF MS/MS spectrum of lysine **2d**-modified YTSSSKNVVR (ESI source, doubly charged ion of *m/z* = 649.8). Inset: the extracted ion chromatogram (EIC) of doubly charged ion of *m/z* = 649.8.

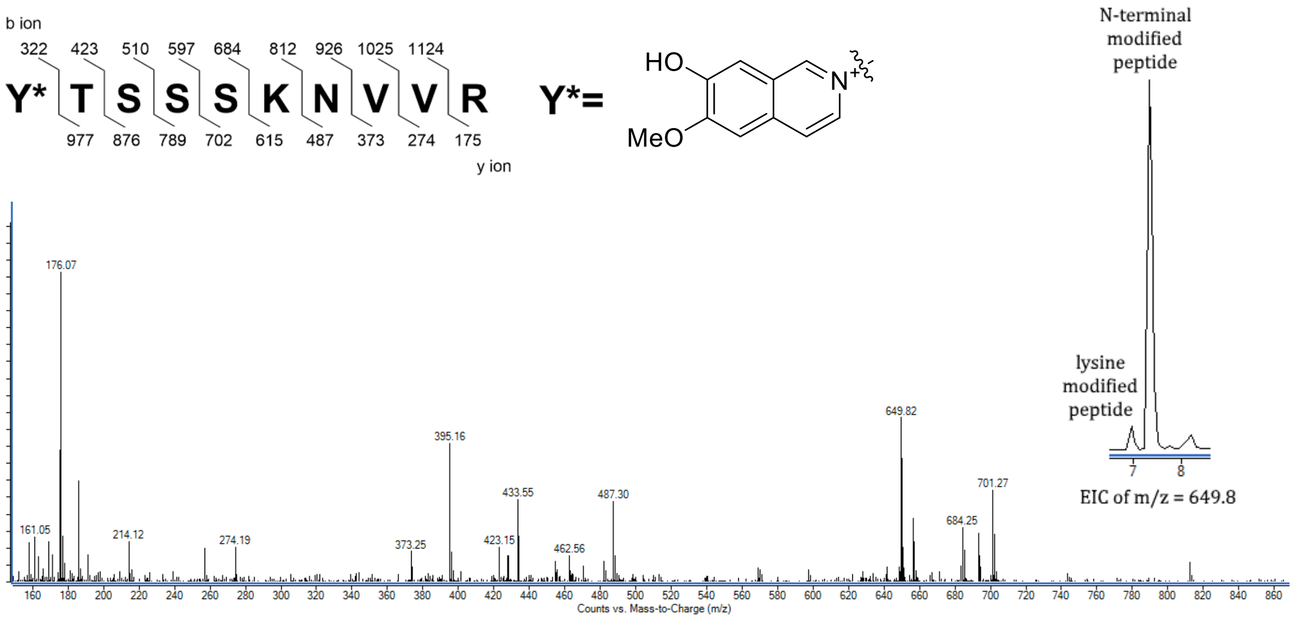

Supplementary Fig. 20 Q-TOF MS/MS spectrum of N-terminal **2e**-modified YTSSSKNVVR (ESI source, doubly charged ion of *m/z* = 649.8). Inset: the extracted ion chromatogram (EIC) of doubly charged ion of *m/z* = 649.8.

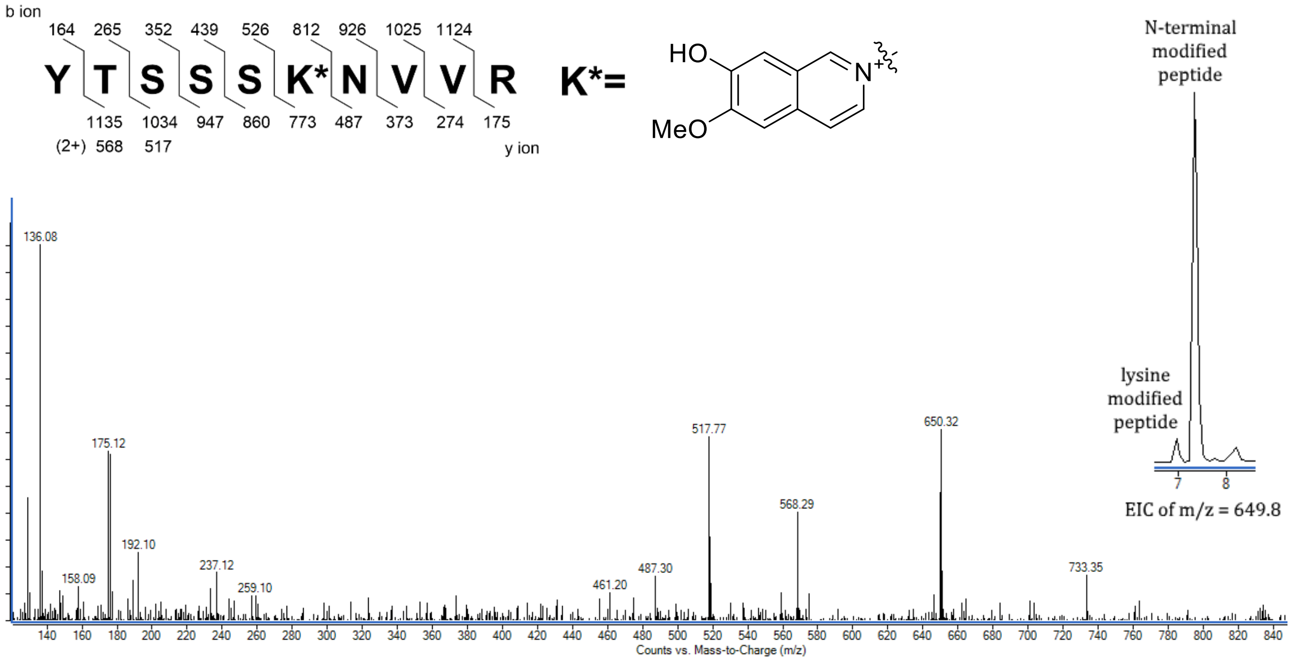

Supplementary Fig. 21 Q-TOF MS/MS spectrum of lysine **2e**-modified YTSSSKNVVR (ESI source, doubly charged ion of *m/z* = 649.8). Inset: the extracted ion chromatogram (EIC) of doubly charged ion of *m/z* = 649.8.

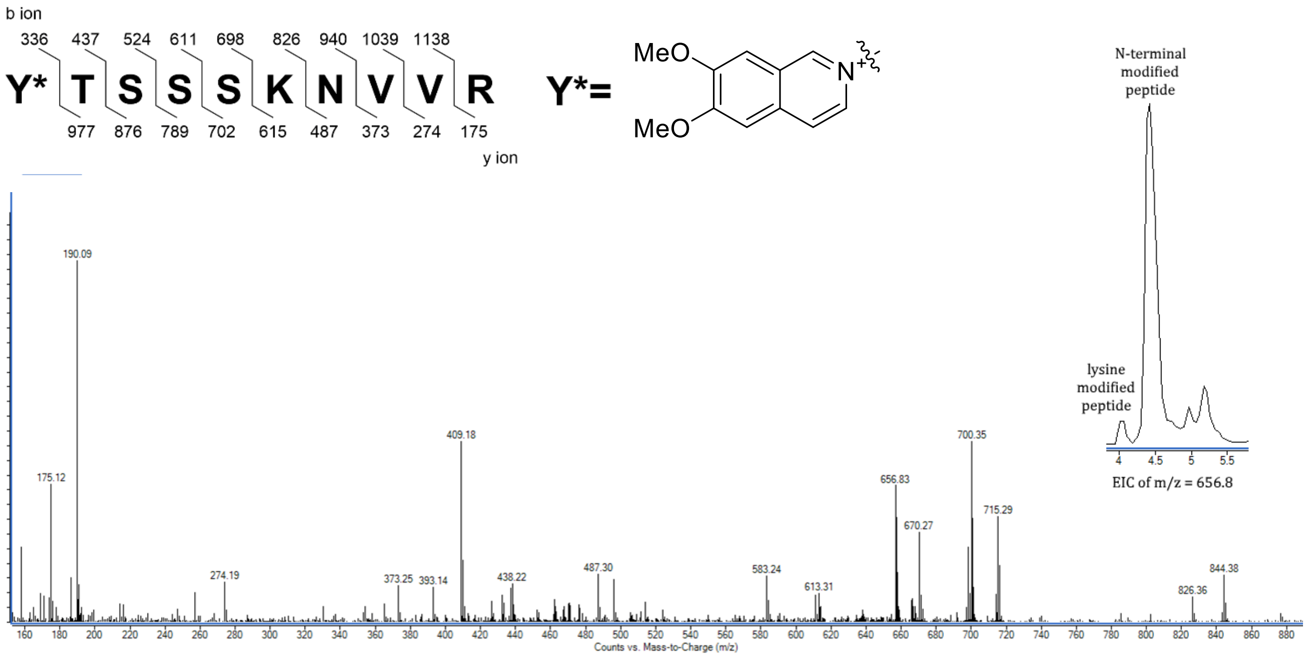

Supplementary Fig. 22 Q-TOF MS/MS spectrum of N-terminal **2f**-modified YTSSSKNVVR (ESI source, doubly charged ion of *m/z* = 656.8). Inset: the extracted ion chromatogram (EIC) of doubly charged ion of *m/z* = 656.8.

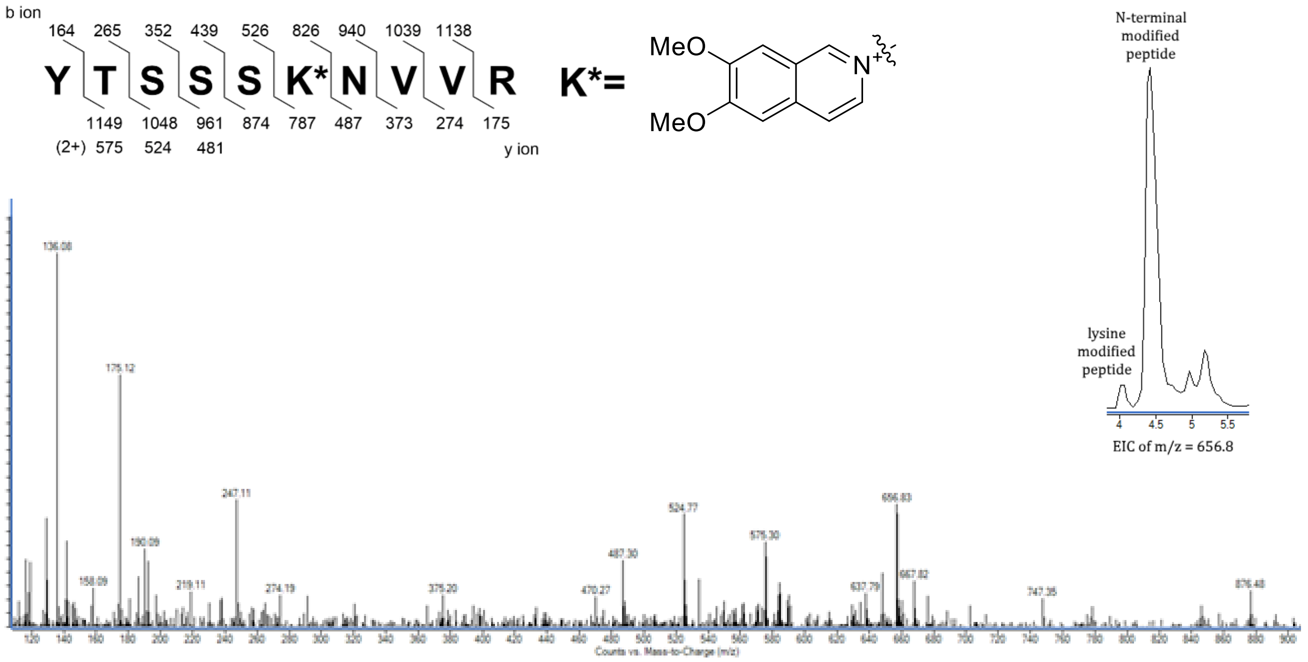

Supplementary Fig. 23 Q-TOF MS/MS spectrum of lysine **2f**-modified YTSSSKNVVR (ESI source, doubly charged ion of *m/z* = 656.8). Inset: the extracted ion chromatogram (EIC) of doubly charged ion of *m/z* = 656.8

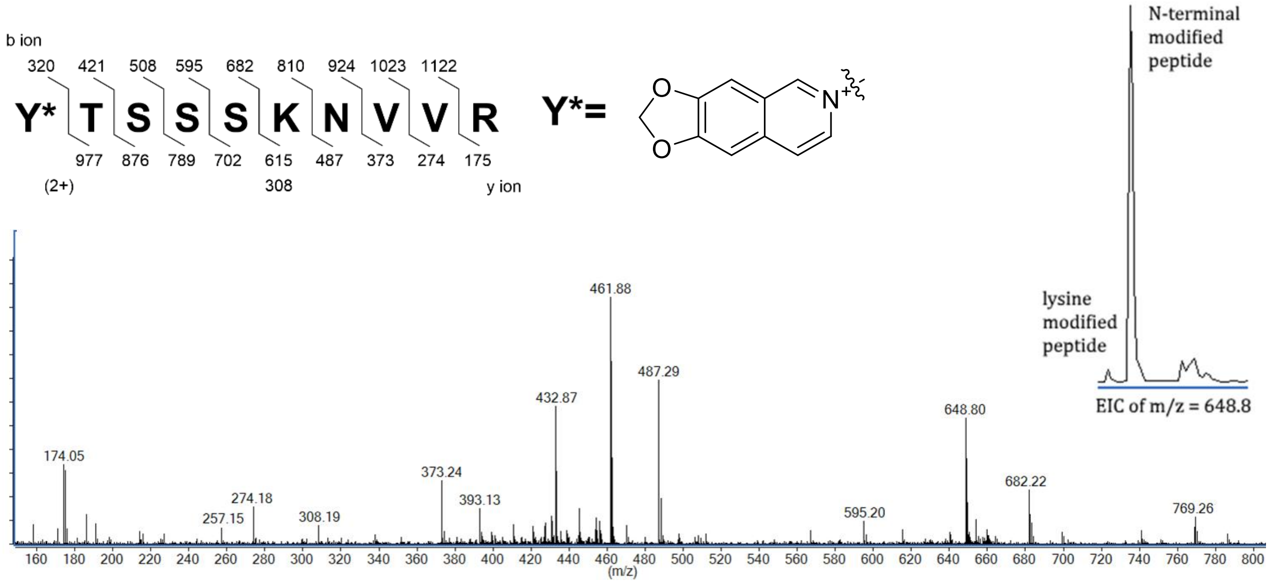

Supplementary Fig. 24 Q-TOF MS/MS spectrum of N-terminal **2g**-modified YTSSSKNVVR (ESI source, doubly charged ion of *m/z* = 648.8). Inset: the extracted ion chromatogram (EIC) of doubly charged ion of *m/z* = 648.8.

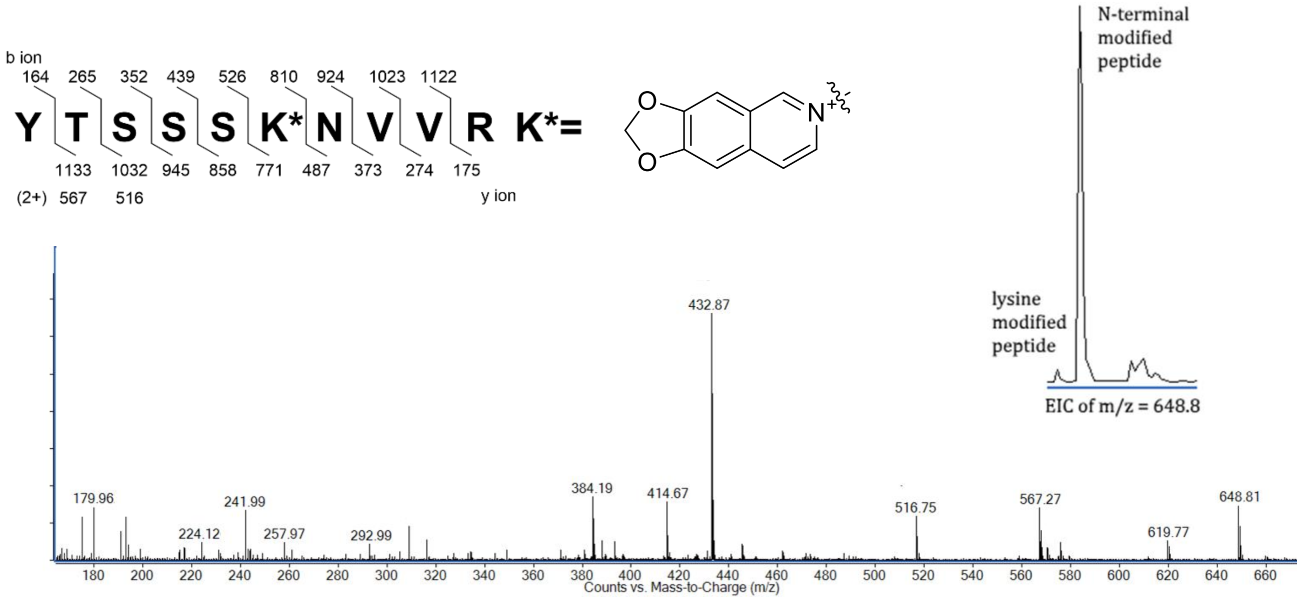

Supplementary Fig. 25 Q-TOF MS/MS spectrum of lysine **2g**-modified YTSSSKNVVR (ESI source, doubly charged ion of *m/z* = 648.8). Inset: the extracted ion chromatogram (EIC) of doubly charged ion of *m/z* = 648.8.

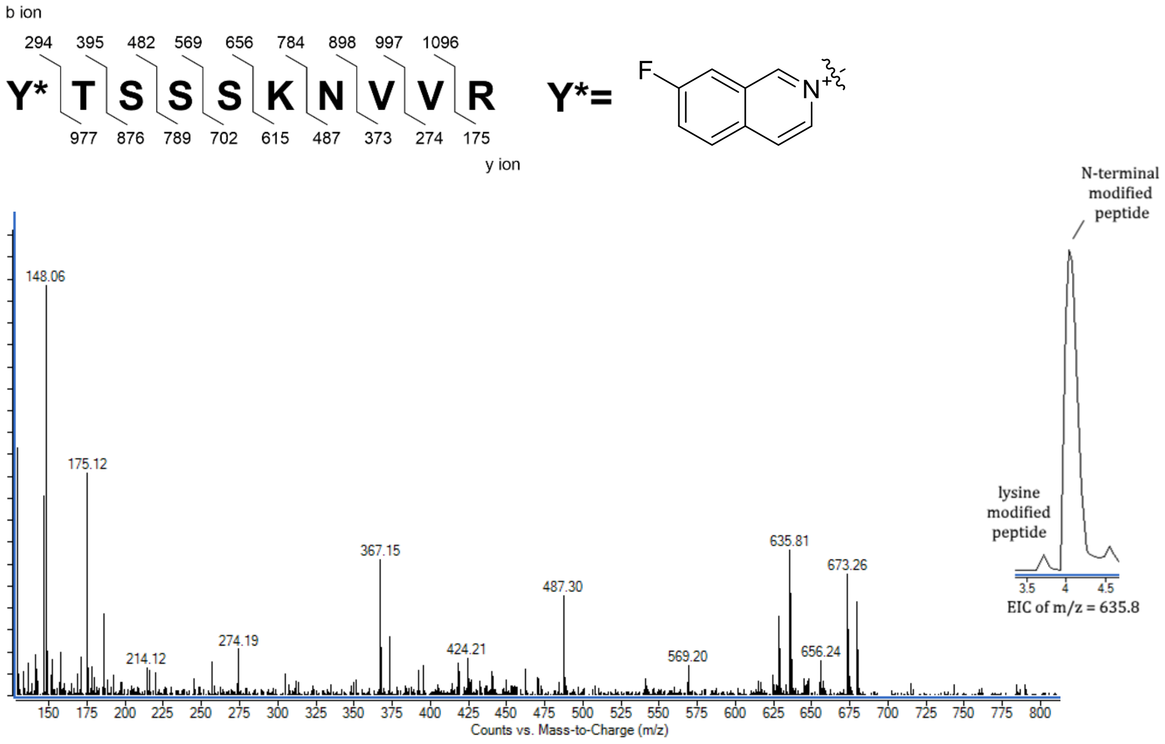

Supplementary Fig. 26 Q-TOF MS/MS spectrum of N-terminal **2h**-modified YTSSSKNVVR (ESI source, doubly charged ion of *m/z* = 635.8). Inset: the extracted ion chromatogram (EIC) of doubly charged ion of *m/z* = 635.8.

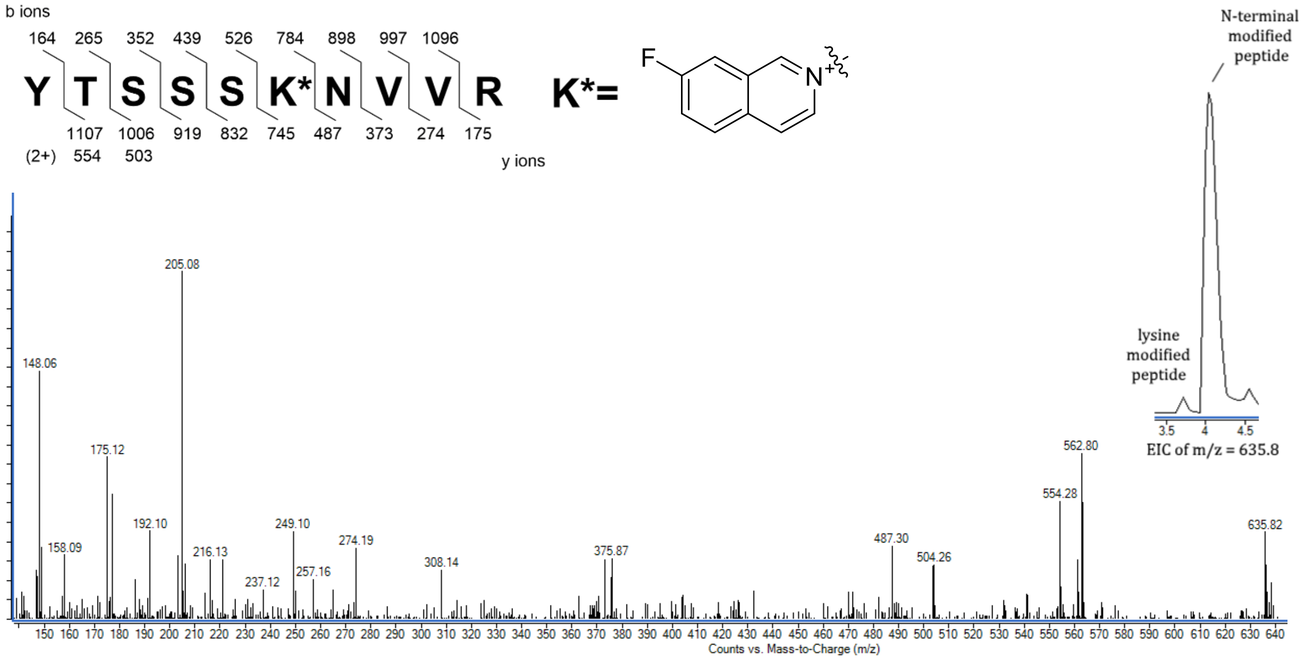

Supplementary Fig. 27 Q-TOF MS/MS spectrum of lysine **2h**-modified YTSSSKNVVR (ESI source, doubly charged ion of *m/z* = 635.8). Inset: the extracted ion chromatogram (EIC) of doubly charged ion of m/z = 635.8.

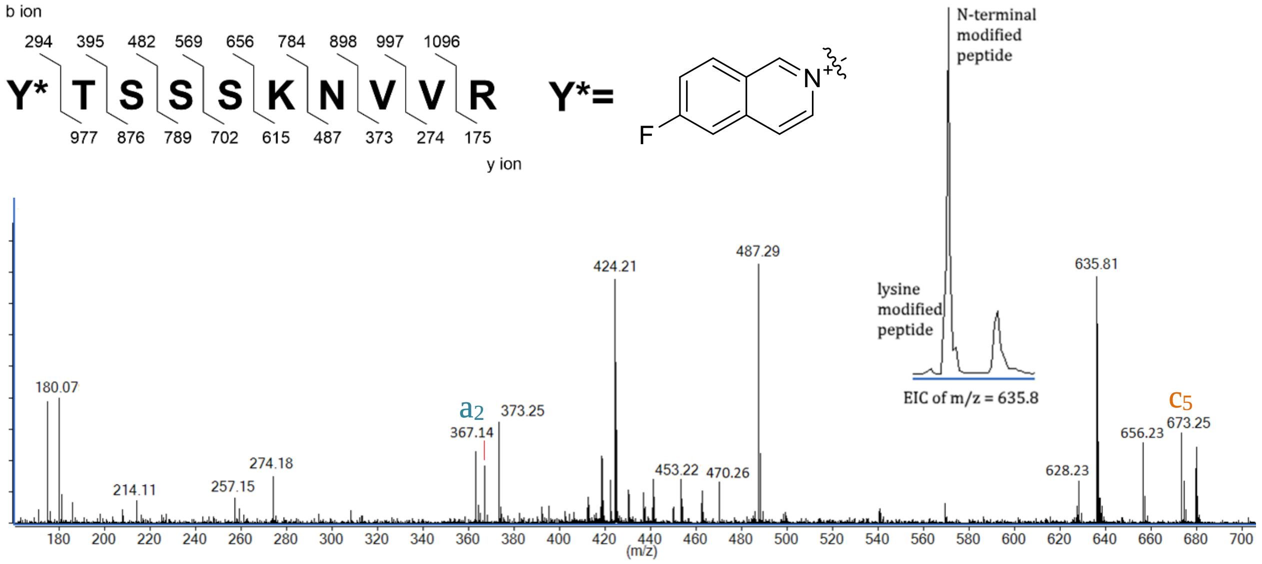

Supplementary Fig. 28 Q-TOF MS/MS spectrum of N-terminal **2i**-modified YTSSSKNVVR (ESI source, doubly charged ion of *m/z* = 635.8). Inset: the extracted ion chromatogram (EIC) of doubly charged ion of *m/z* = 635.8.

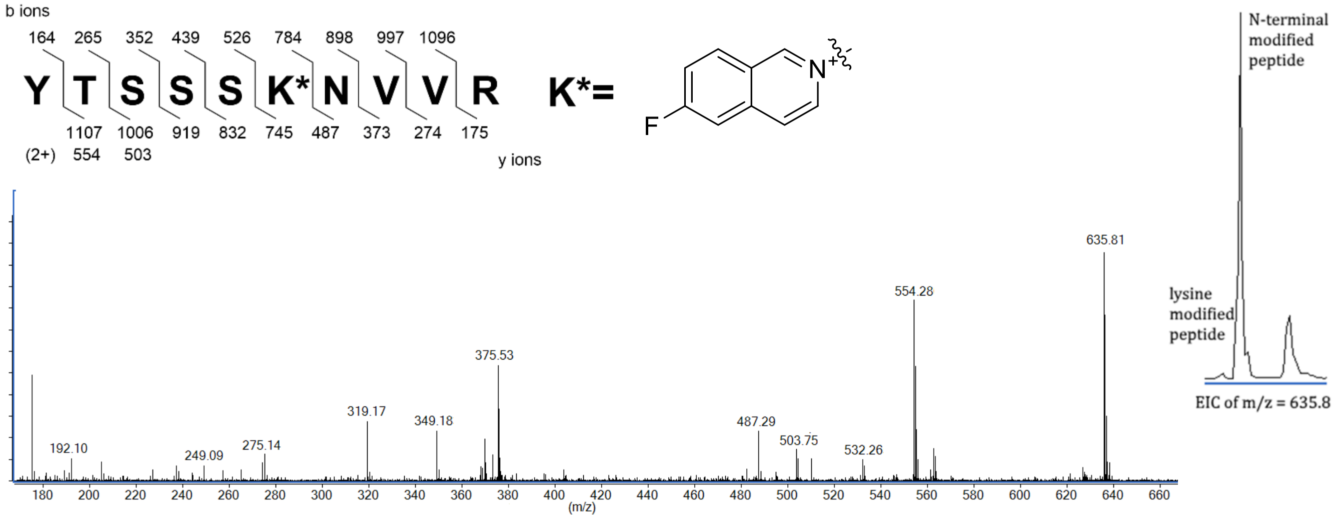

Supplementary Fig. 29 Q-TOF MS/MS spectrum of lysine **2i**-modified YTSSSKNVVR (ESI source, doubly charged ion of *m/z* = 635.8). Inset: the extracted ion chromatogram (EIC) of doubly charged ion of *m/z* = 635.8.

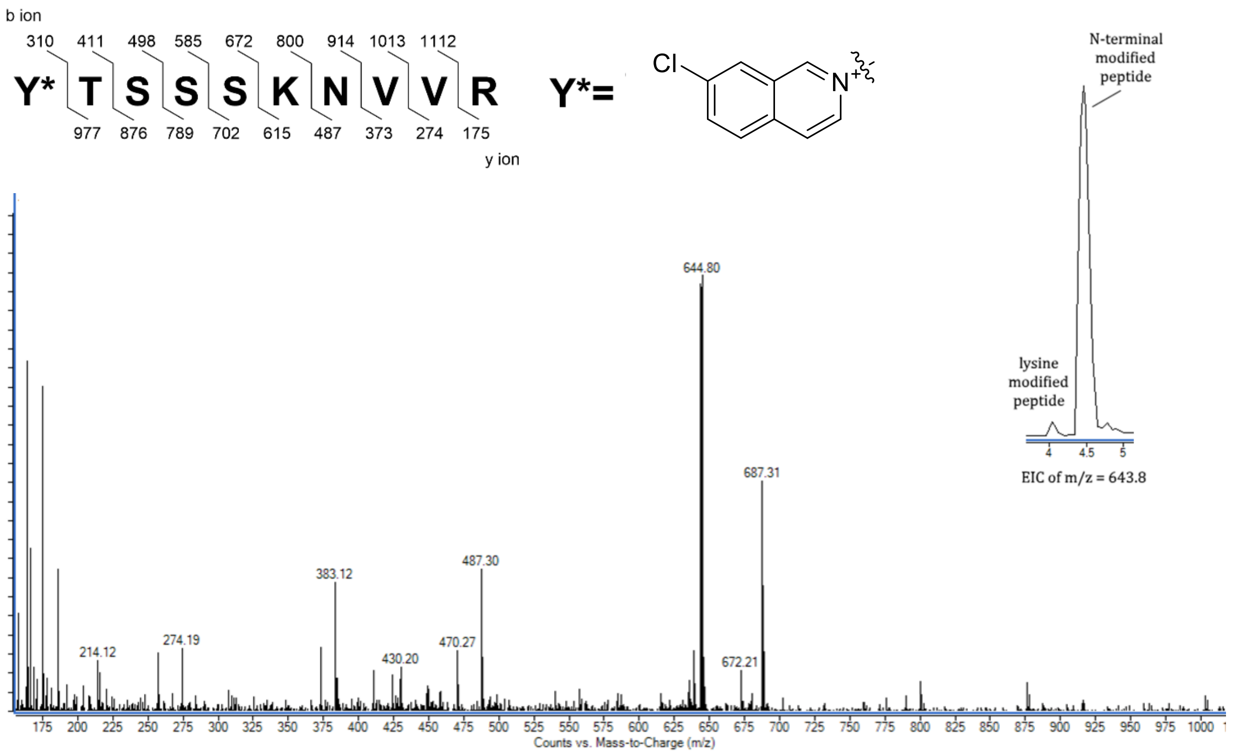

Supplementary Fig. 30 Q-TOF MS/MS spectrum of N-terminal **2j**-modified YTSSSKNVVR (ESI source, doubly charged ion of *m/z* = 643.8). Inset: the extracted ion chromatogram (EIC) of doubly charged ion of *m/z* = 643.8.

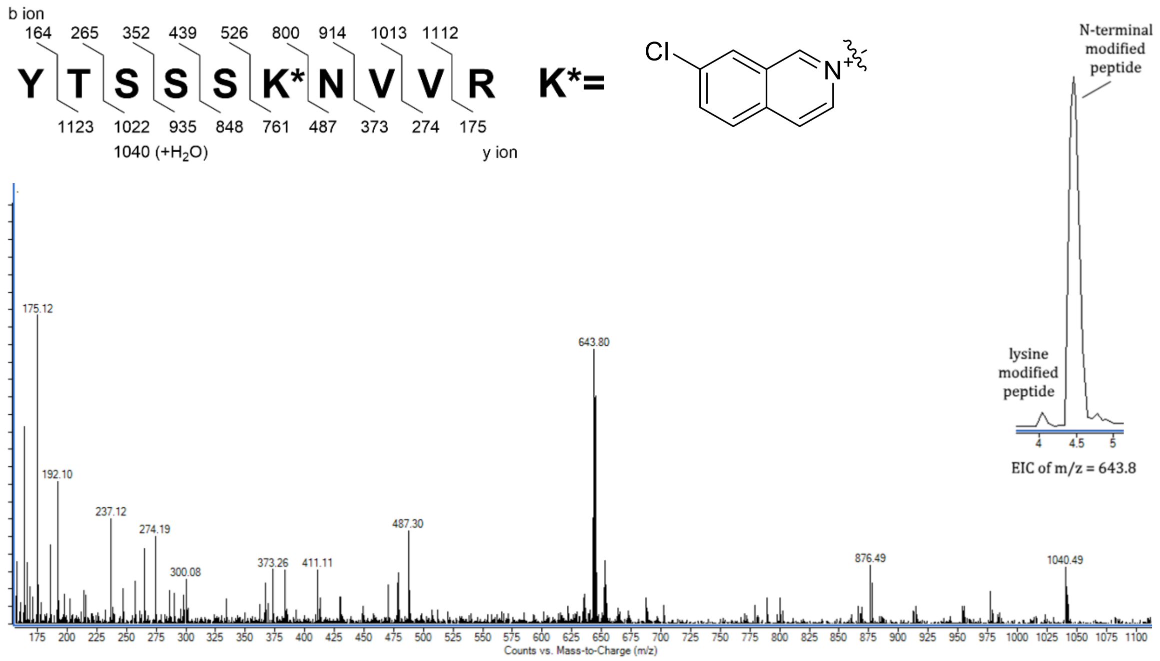

Supplementary Fig. 31 Q-TOF MS/MS spectrum of lysine **2j**-modified YTSSSKNVVR (ESI source, doubly charged ion of *m/z* = 643.8). Inset: the extracted ion chromatogram (EIC) of doubly charged ion of *m/z* = 643.8.

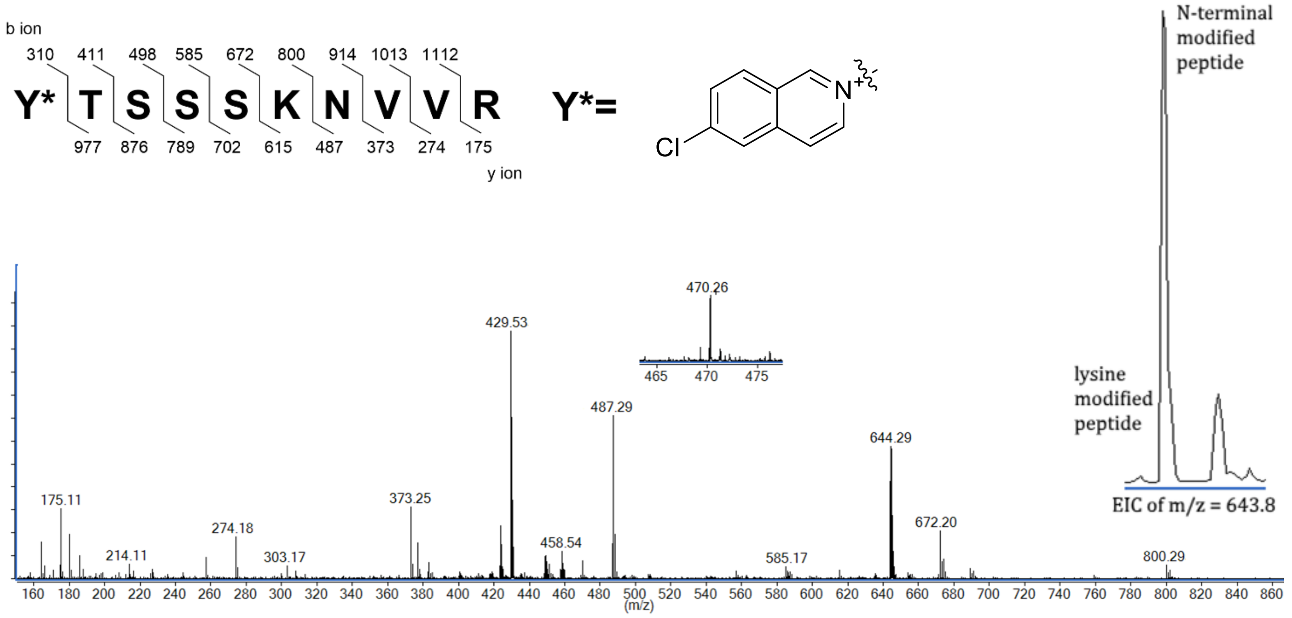

Supplementary Fig. 32 Q-TOF MS/MS spectrum of N-terminal **2k**-modified YTSSSKNVVR (ESI source, doubly charged ion of *m/z* = 643.8). Inset: the extracted ion chromatogram (EIC) of doubly charged ion of *m/z* = 643.8.

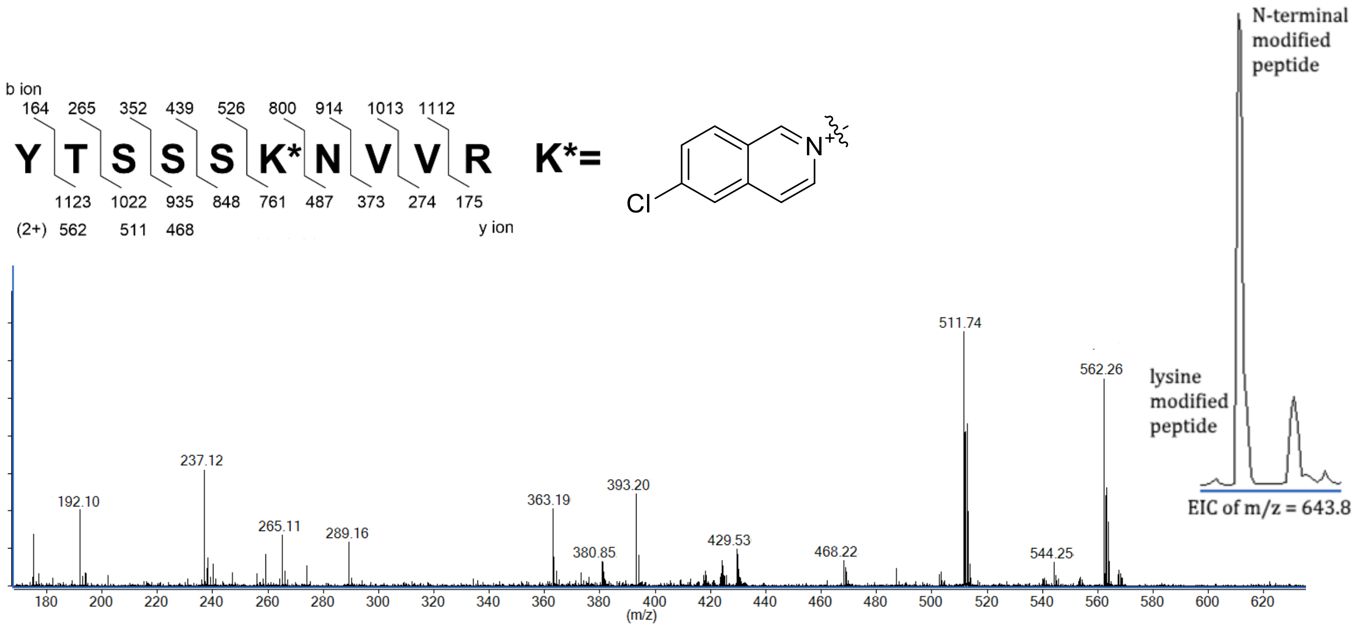

Supplementary Fig. 33 Q-TOF MS/MS spectrum of lysine **2k**-modified YTSSSKNVVR (ESI source, doubly charged ion of *m/z* = 643.8). Inset: the extracted ion chromatogram (EIC) of doubly charged ion of *m/z* = 643.8.

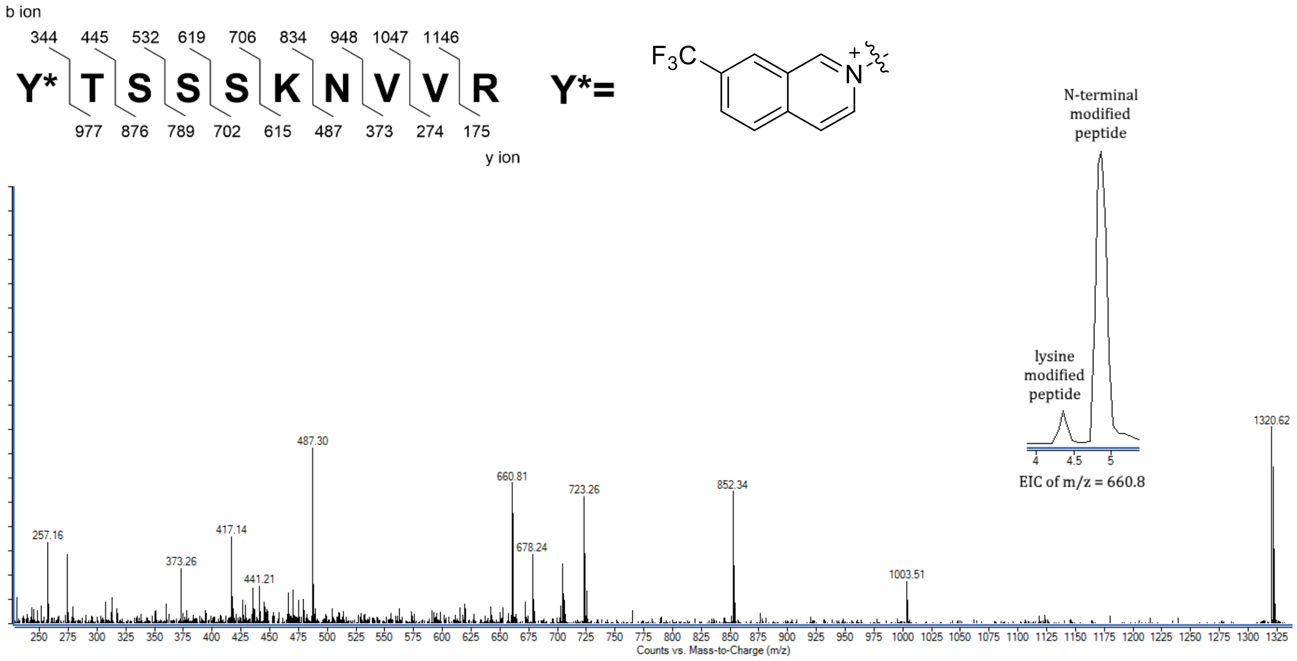

Supplementary Fig. 34 Q-TOF MS/MS spectrum of N-terminal **2l**-modified YTSSSKNVVR (ESI source, doubly charged ion of *m/z* = 660.8). Inset: the extracted ion chromatogram (EIC) of doubly charged ion of *m/z* = 660.8.

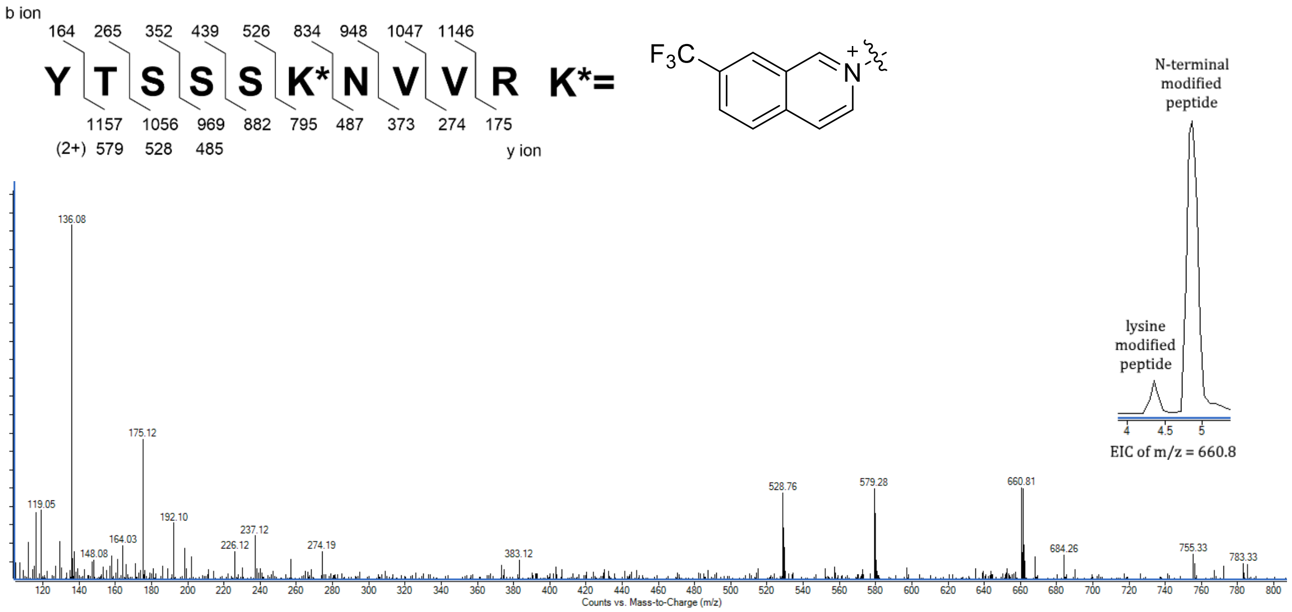

Supplementary Fig. 35 Q-TOF MS/MS spectrum of lysine **2l**-modified YTSSSKNVVR (ESI source, doubly charged ion of *m/z* = 660.8). Inset: the extracted ion chromatogram (EIC) of doubly charged ion of *m/z* = 660.8.

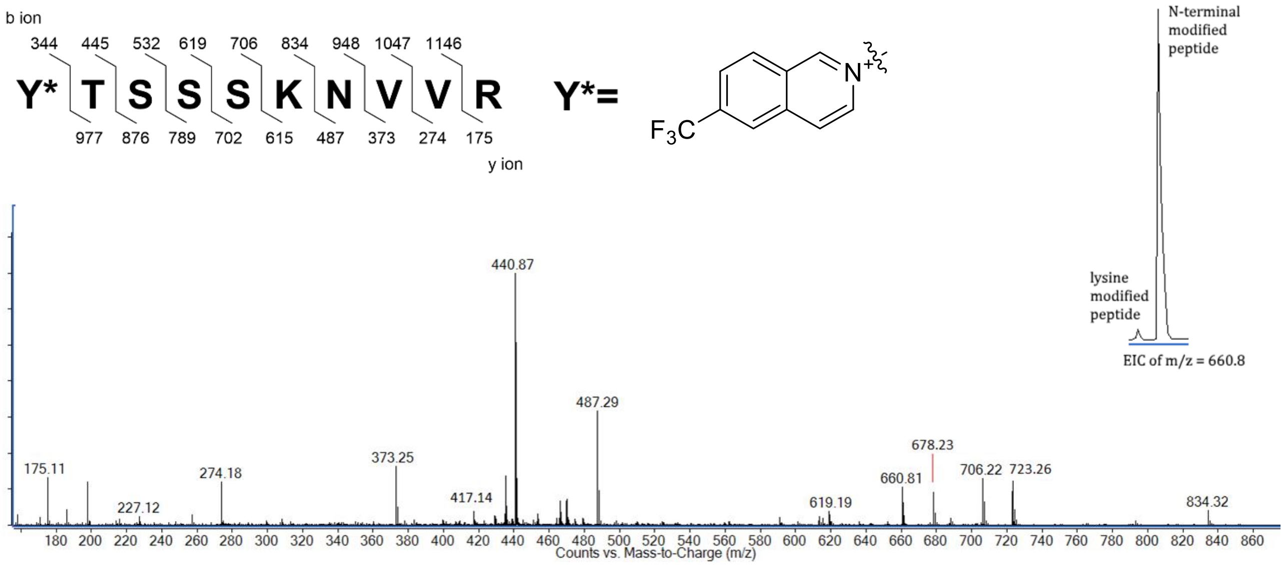

Supplementary Fig. 36 Q-TOF MS/MS spectrum of N-terminal **2m**-modified YTSSSKNVVR (ESI source, doubly charged ion of *m/z* = 660.8). Inset: the extracted ion chromatogram (EIC) of doubly charged ion of *m/z* = 660.8.

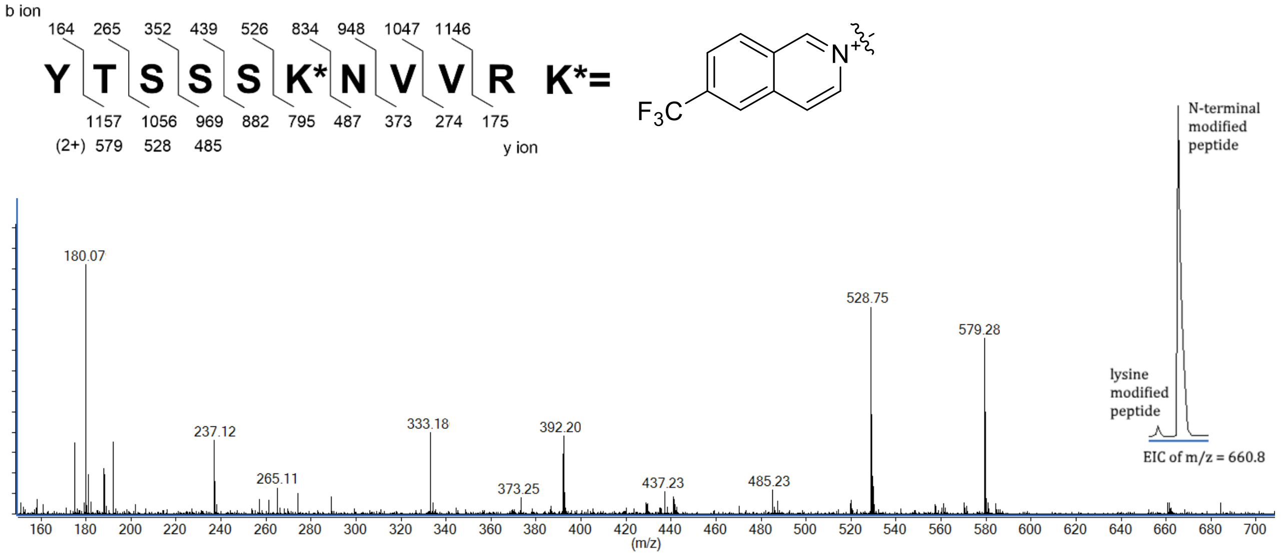

Supplementary Fig. 37 Q-TOF MS/MS spectrum of lysine **2m**-modified YTSSSKNVVR (ESI source, doubly charged ion of *m/z* = 660.8). Inset: the extracted ion chromatogram (EIC) of doubly charged ion of *m/z* = 660.8.

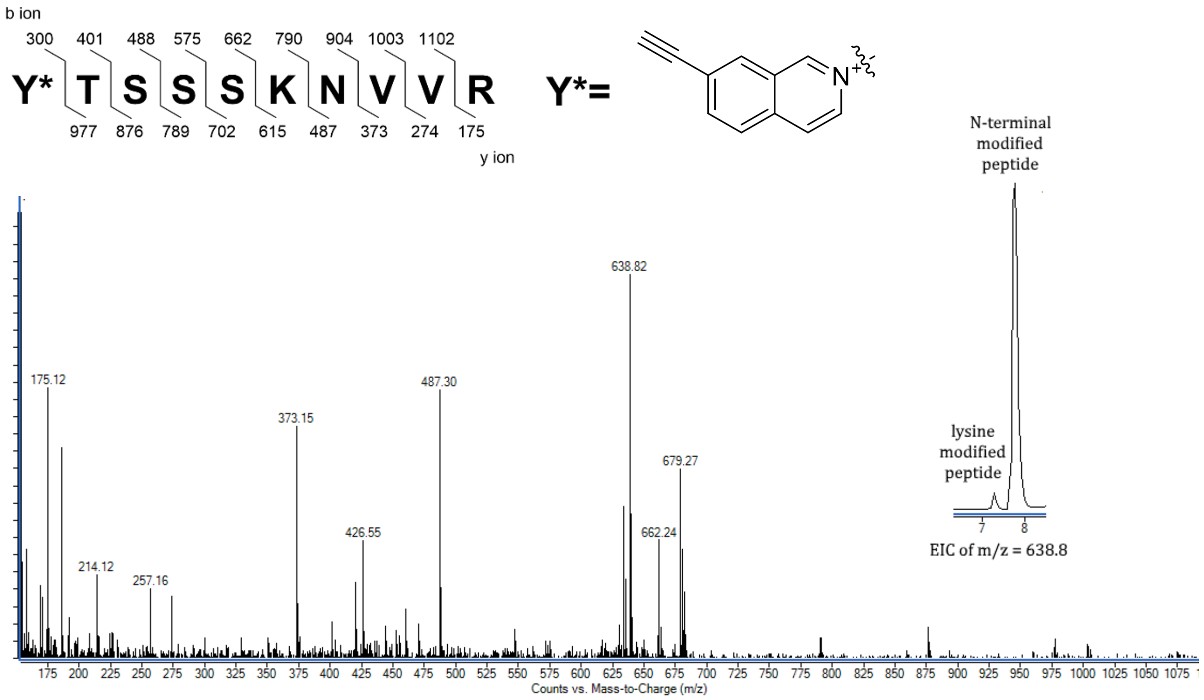

Supplementary Fig. 38 Q-TOF MS/MS spectrum of N-terminal **2n**-modified YTSSSKNVVR (ESI source, doubly charged ion of m/z = 638.8). Inset: the extracted ion chromatogram (EIC) of doubly charged ion of *m/z* = 638.8.

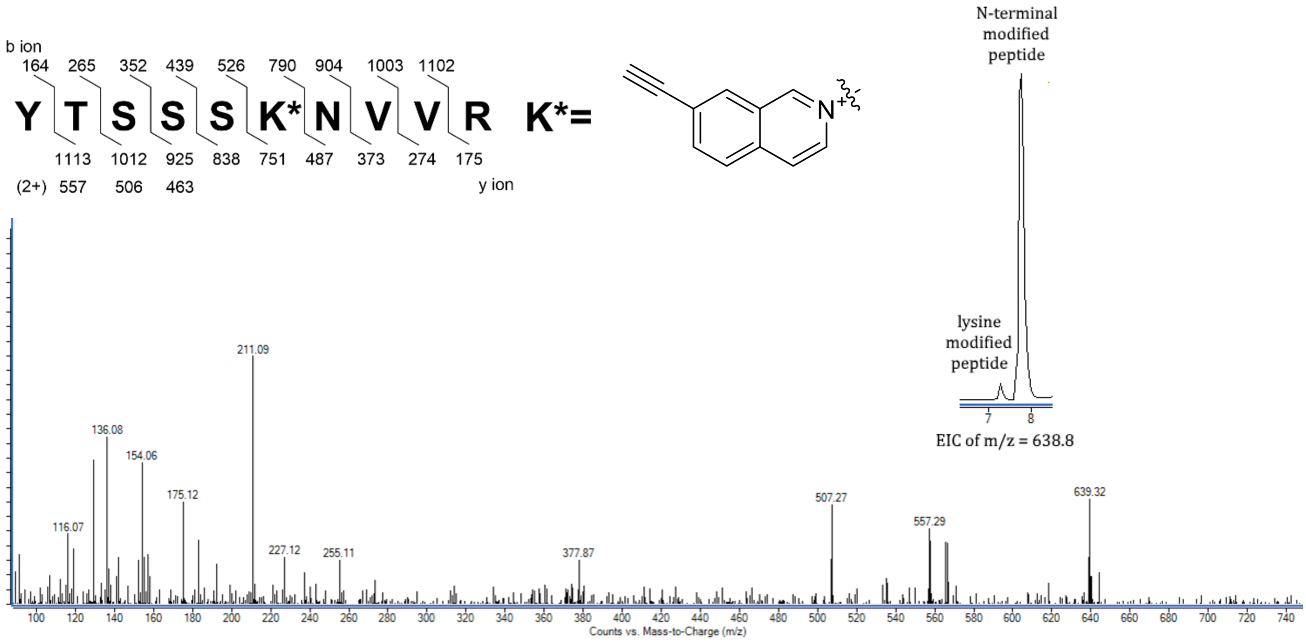

Supplementary Fig. 39 Q-TOF MS/MS spectrum of lysine **2n**-modified YTSSSKNVVR (ESI source, doubly charged ion of *m/z* = 638.8). Inset: the extracted ion chromatogram (EIC) of doubly charged ion of *m/z* = 638.8.

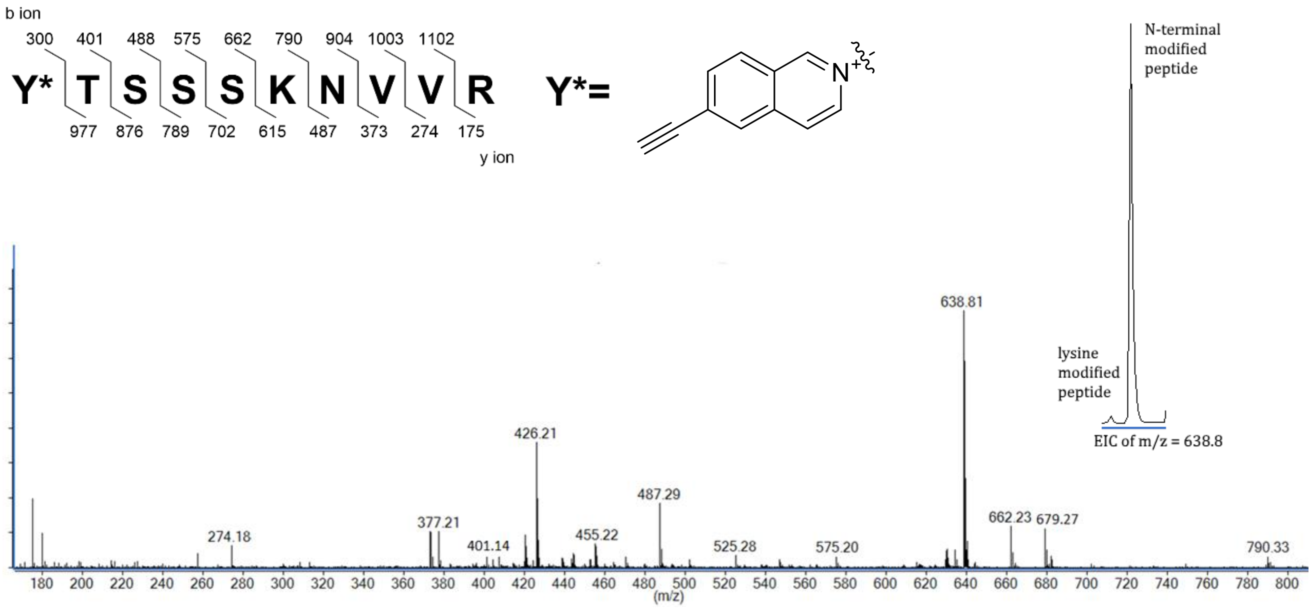

Supplementary Fig. 40 Q-TOF MS/MS spectrum of N-terminal **2o**-modified YTSSSKNVVR (ESI source, doubly charged ion of *m/z* = 638.8). Inset: the extracted ion chromatogram (EIC) of doubly charged ion of *m/z* = 638.8.

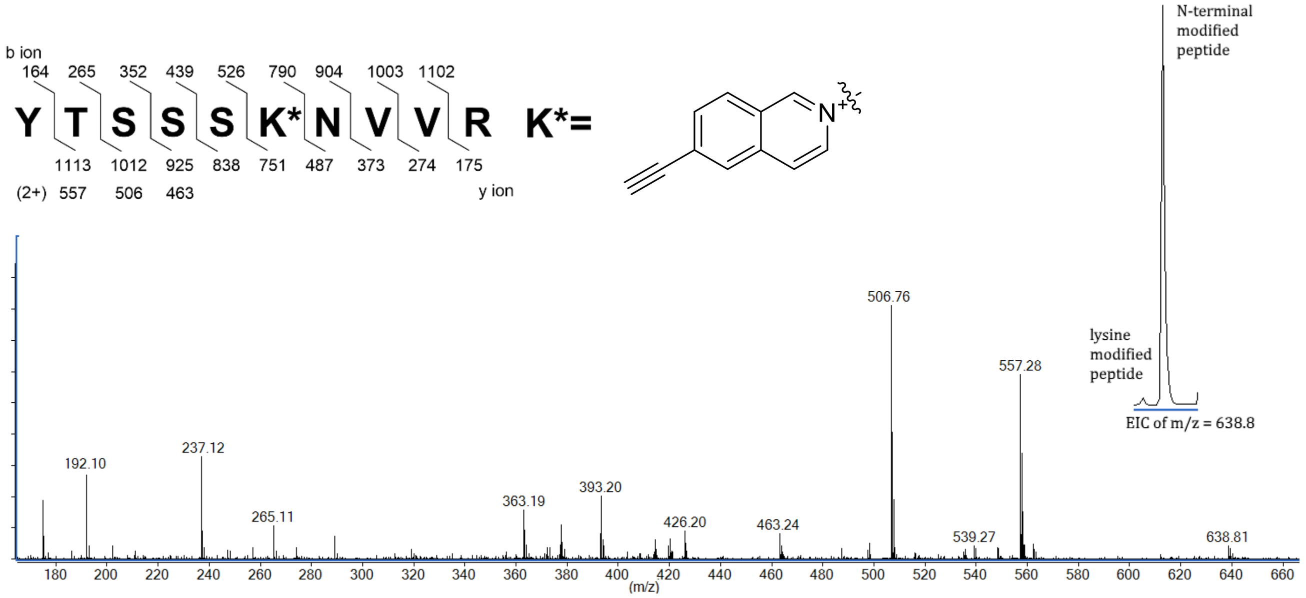

Supplementary Fig. 41 Q-TOF MS/MS spectrum of lysine **2o**-modified YTSSSKNVVR (ESI source, doubly charged ion of *m/z* = 638.8). Inset: the extracted ion chromatogram (EIC) of doubly charged ion of *m/z* = 638.8.

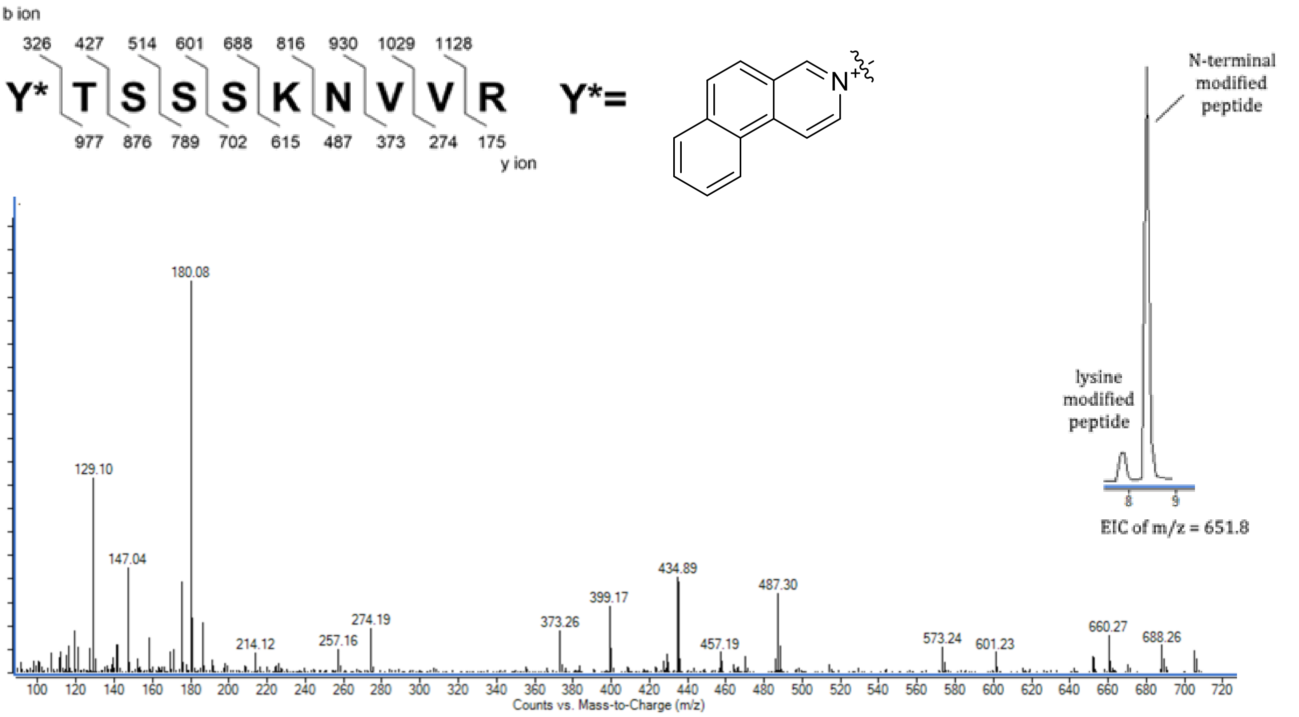

Supplementary Fig. 42 Q-TOF MS/MS spectrum of N-terminal **2p**-modified YTSSSKNVVR (ESI source, doubly charged ion of *m/z* = 651.8). Inset: the extracted ion chromatogram (EIC) of doubly charged ion of *m/z* = 651.8.

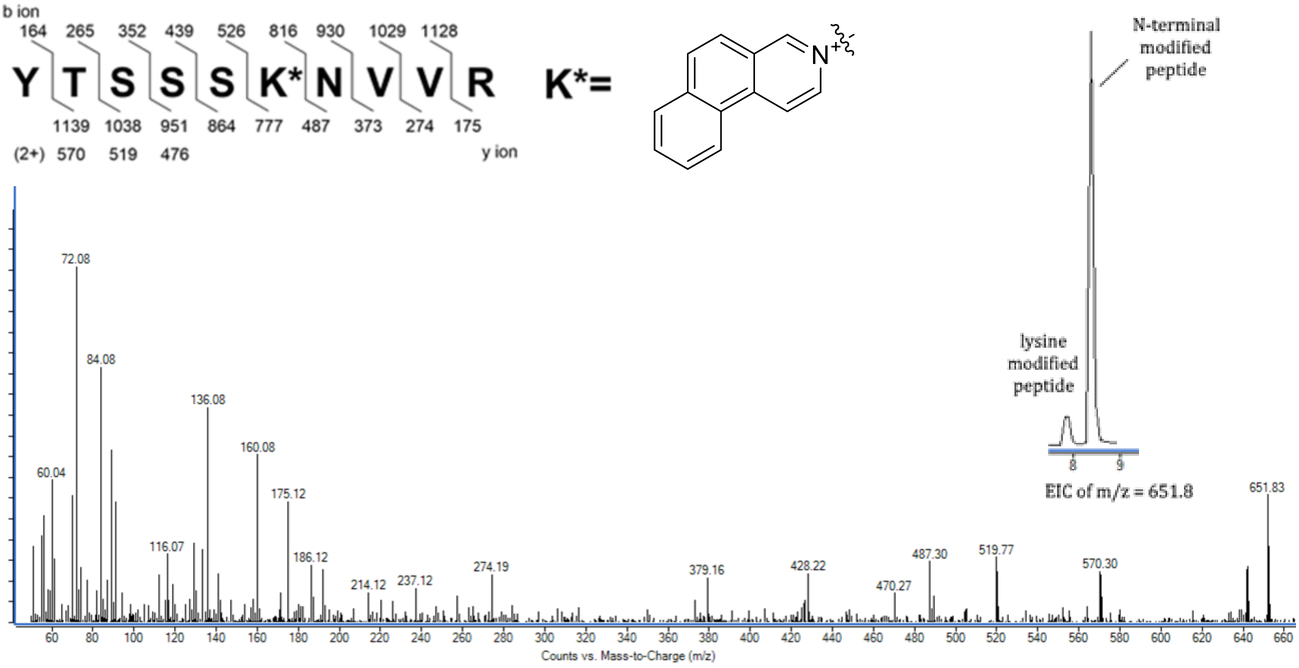

Supplementary Fig. 43 Q-TOF MS/MS spectrum of lysine **2p**-modified YTSSSKNVVR (ESI source, doubly charged ion of *m/z* = 651.8). Inset: the extracted ion chromatogram (EIC) of doubly charged ion of *m/z* = 651.8.

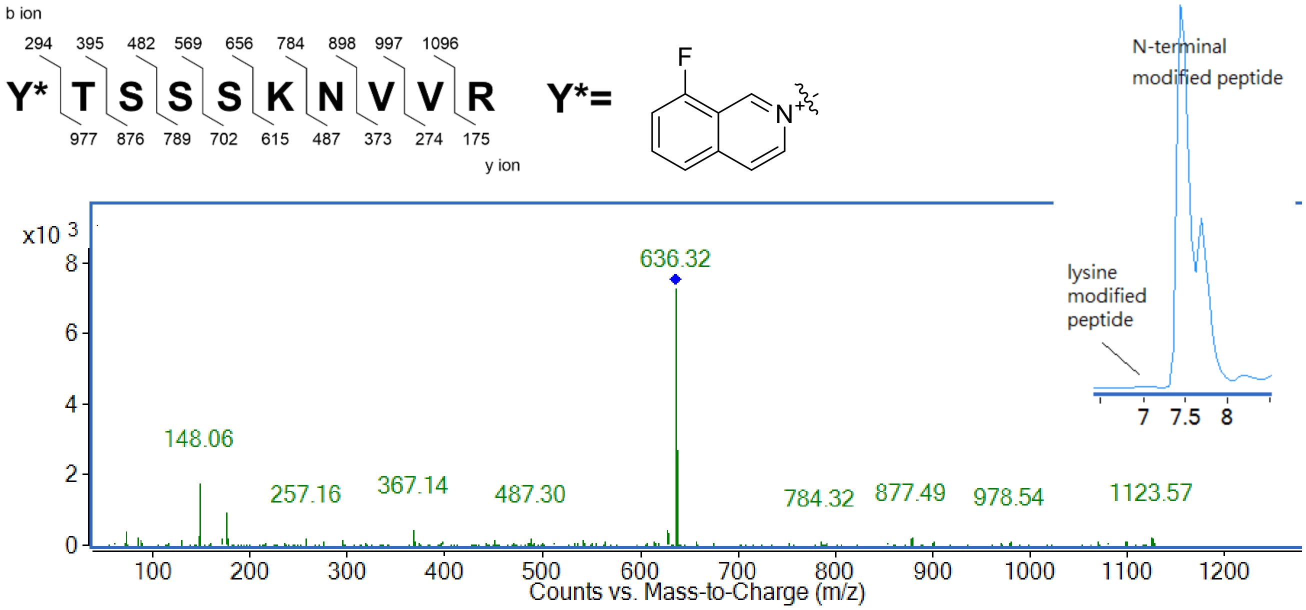

Supplementary Fig. 44 Q-TOF MS/MS spectrum of N-terminal **2q**-modified YTSSSKNVVR (ESI source, doubly charged ion of *m/z* = 635.8). Inset: the extracted ion chromatogram (EIC) of doubly charged ion of *m/z* = 635.8.

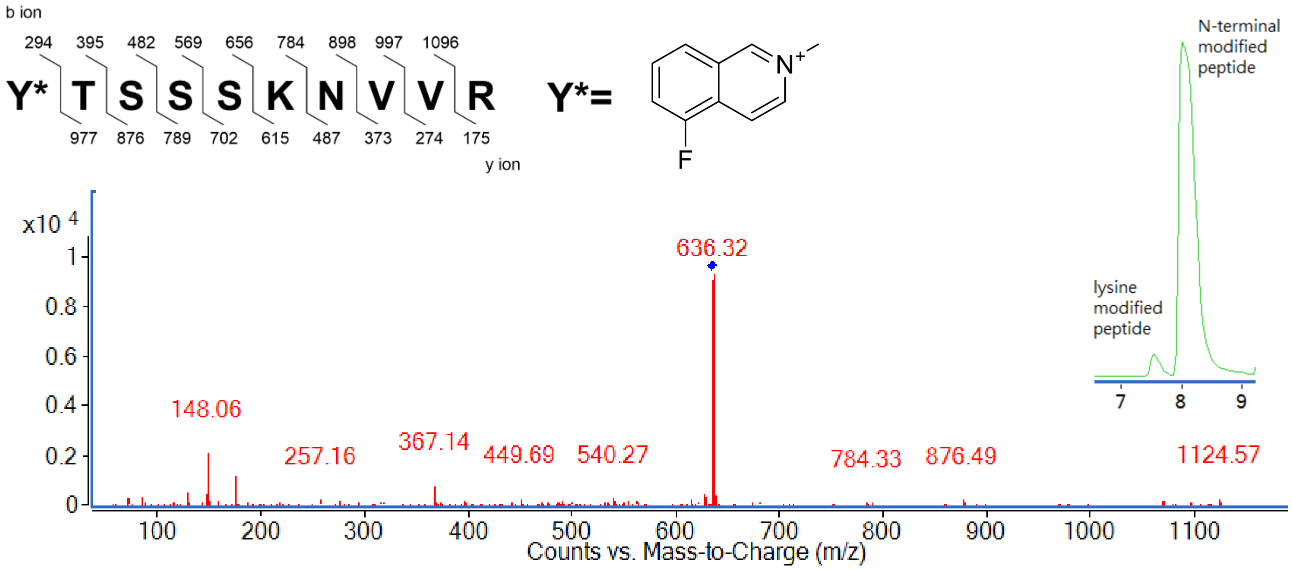

Supplementary Fig. 45 Q-TOF MS/MS spectrum of N-terminal **2r**-modified YTSSSKNVVR (ESI source, doubly charged ion of *m/z* = 635.8). Inset: the extracted ion chromatogram (EIC) of doubly charged ion of *m/z* = 635.8.

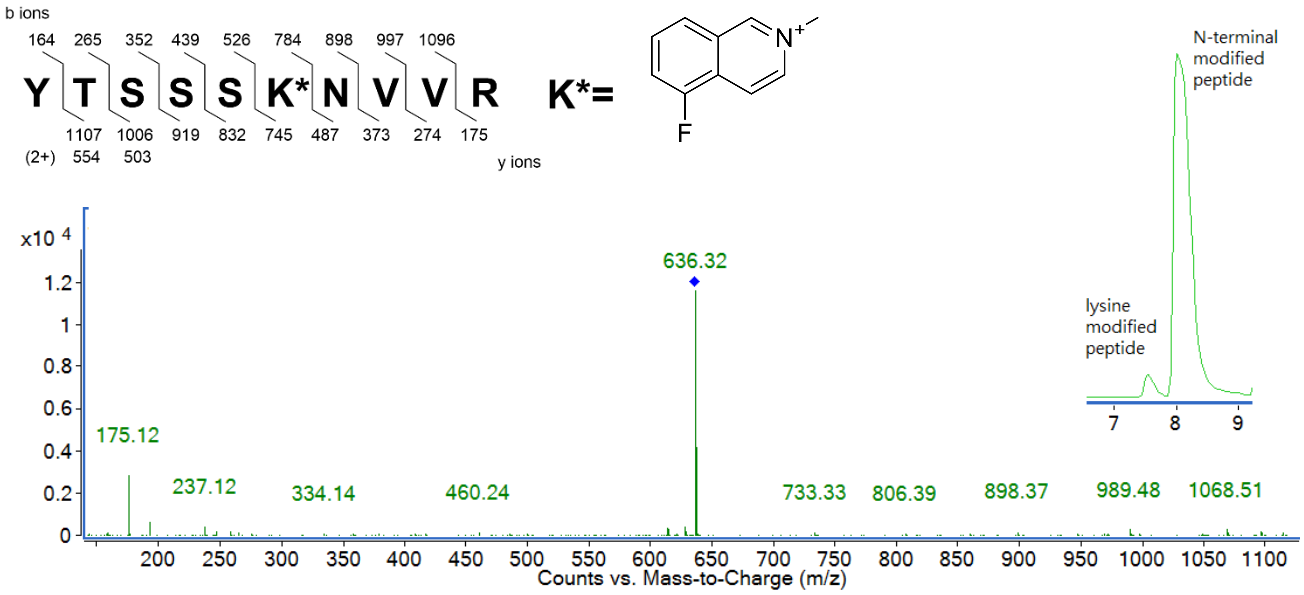

Supplementary Fig. 46 Q-TOF MS/MS spectrum of lysine **2r**-modified YTSSSKNVVR (ESI source, doubly charged ion of *m/z* = 635.8). Inset: the extracted ion chromatogram (EIC) of doubly charged ion of *m/z* = 635.8.

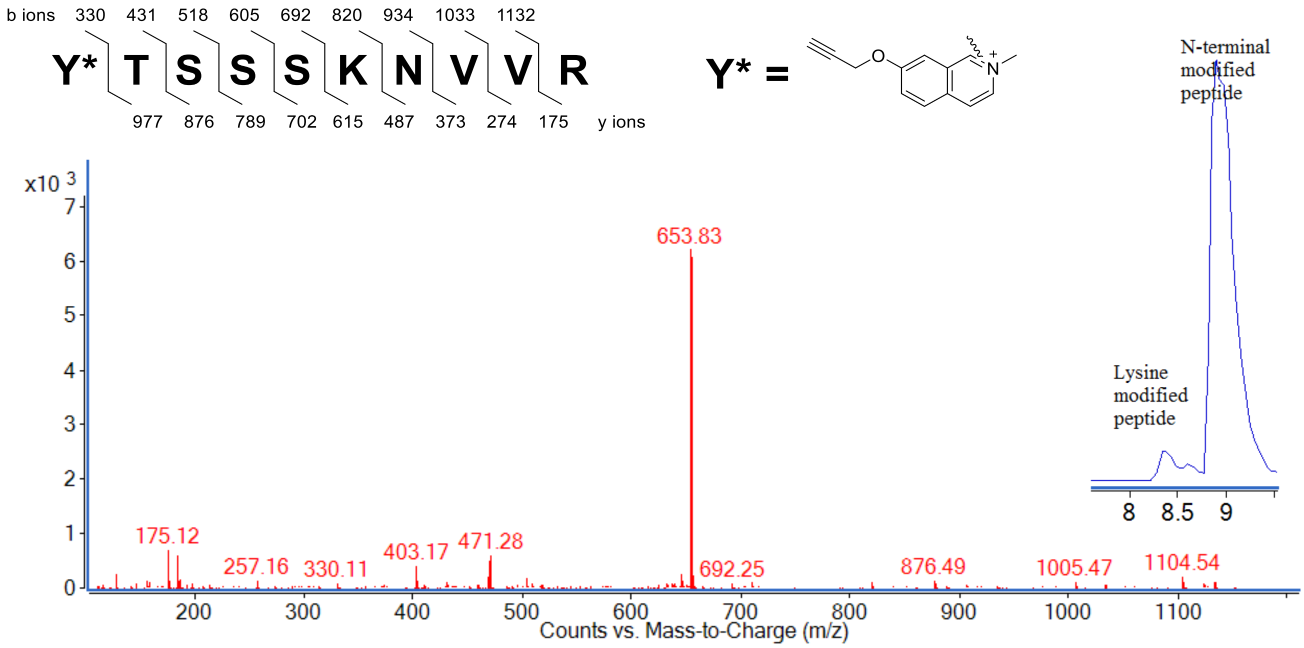

Supplementary Fig. 47 Q-TOF MS/MS spectrum of N-terminal **2t**-modified YTSSSKNVVR (ESI source, doubly charged ion of *m/z* = 653.8). Inset: the extracted ion chromatogram (EIC) of doubly charged ion of *m/z* = 653.8.

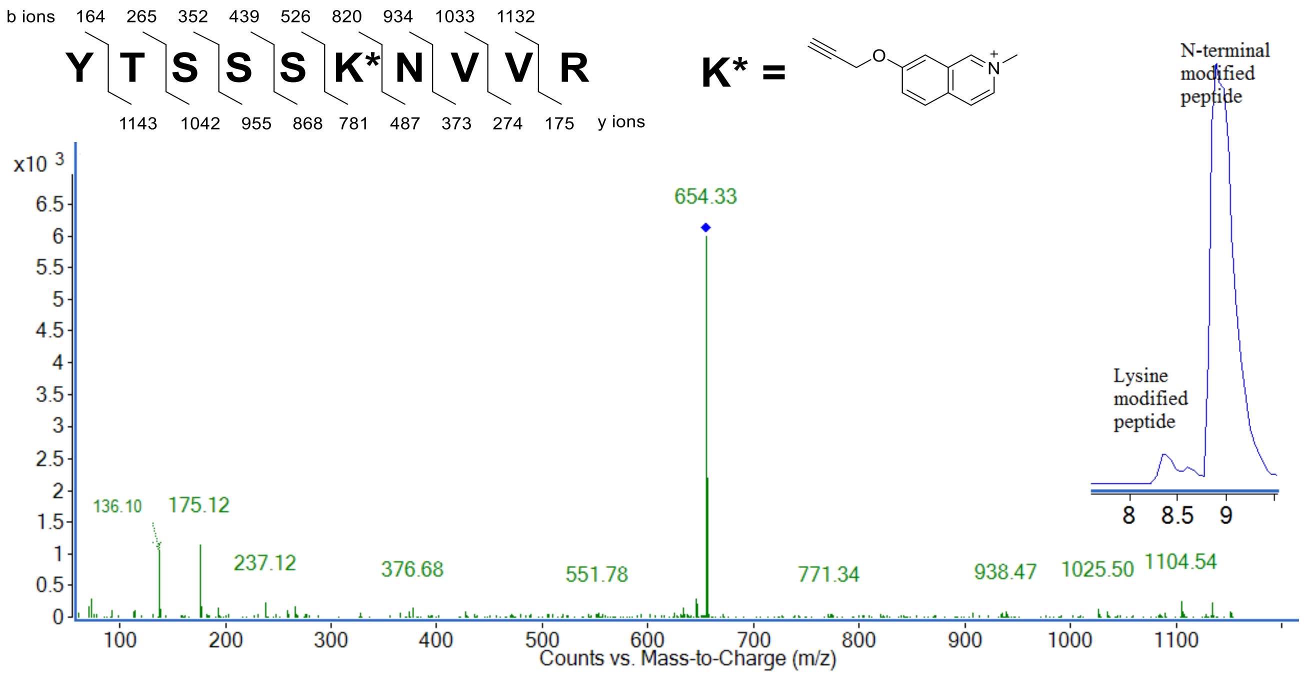

Supplementary Fig. 48 Q-TOF MS/MS spectrum of lysine **2t**-modified YTSSSKNVVR (ESI source, doubly charged ion of *m/z* = 653.8). Inset: the extracted ion chromatogram (EIC) of doubly charged ion of *m/z* = 653.8.

Supplementary Table 2. Condition optimization of N-terminal modification of peptide YTSSSKNVVR **1a** using **2d**.[a]

Entry	**2d** (equiv.)	Temperature (^{o}C)	Conversion (%)[b]				N-terminal selectivity of mono-modified peptide[c]	Efficiency of N-terminal modification[e]
			Mono-modified[d]		Di-modified	Total		
			N-terminus	Lysine				
1	5	37	21	-	2	23	>99:1	1.0
2	10	37	32	1	4	38	97:3	0.84
3	15	37	46	1	5	52	98:2	0.88
4	20	37	73	-	13	86	>99:1	1.0
5	20	25	16	-	0	16	>99:1	1.0
6	20	4	2.4	1.6	0	4	60:40	0.6

[a]Conditions: YTSSSKNVVR **1a** (0.1 mM) and **2d** in 50 mM PBS (pH 6.5)/DMSO (9:1) solution (100 μL), 37 ^{o}C, 16 h. [b]Determined by total ion count (TIC) of LC-MS analysis. [c]N-terminal selectivity is obtained by ratio of mono-modified peptide at N-terminal α-amino group to lysine ε-amino group as determined by extracted ion chromatogram (EIC) of LC-MS analysis. [d]Conversion of N-terminal modified peptide (or lysine-modified peptide) is determined by the conversion of mono-modified peptide and N-terminal selectivity of mono-modified peptide. [e]Modification was conducted at room temperature for 15 min. [e]Efficiency of N-terminal modification is equal to the conversion of N-terminal modified peptide over the total conversion.

Time Course Experiments on the Modification of YTSSSKNVVR (1a) with 2-Ethynyl-5-hydroxy-4-methoxy-benzaldehyde (2d) in Different pH values of 50 mM Phosphate-Buffered Saline and DMSO (9:1)

A mixture of 50 µL of YTSSSKNVVR **1a** (1 mM in Milli-Q® water), 50 µL of 2-EBA **2d** (20 mM in DMSO) and 400 µL of 50 mM PBS buffer with different pH values (6.5, 7.4, 8.0 and 9.0) was treated in a 1.5 mL Eppendorf tube at 37 ^{o}C for 0-24 h. At each time point, 20 µL of the resulting mixture was collected and diluted with 20 µL of Milli-Q water. The resulting mixture was characterized by LC-MS and LC-MS/MS analysis to determine the conversion.

Studies on the Stability of the N-terminally 2d-modified YTSSSKNVVR

An 50 µL aliquot of N-terminally **2d**-modified YTSSSKNVVR (0.1 mM in 50 mM pH 6.5 PBS buffer/CH_3CN (9:1)) and 50 µL of additive (glutathione (GSH), homocysteine, L-cysteine, DL-dithiothreitol (DTT), 2-mercaptoethanol, tris(2-carboxyethyl)phosphine (TCEP), ascorbic acid or hydrogen peroxide; 50 mM in H_2O) was treated in a 1.5 mL Eppendorf tube at 37 ^oC for 2 h. The resulting mixture was characterized by LC-MS and LC-MS/MS analysis to determine the percentage of N-terminally **2d**-modified YTSSSKNVVR.

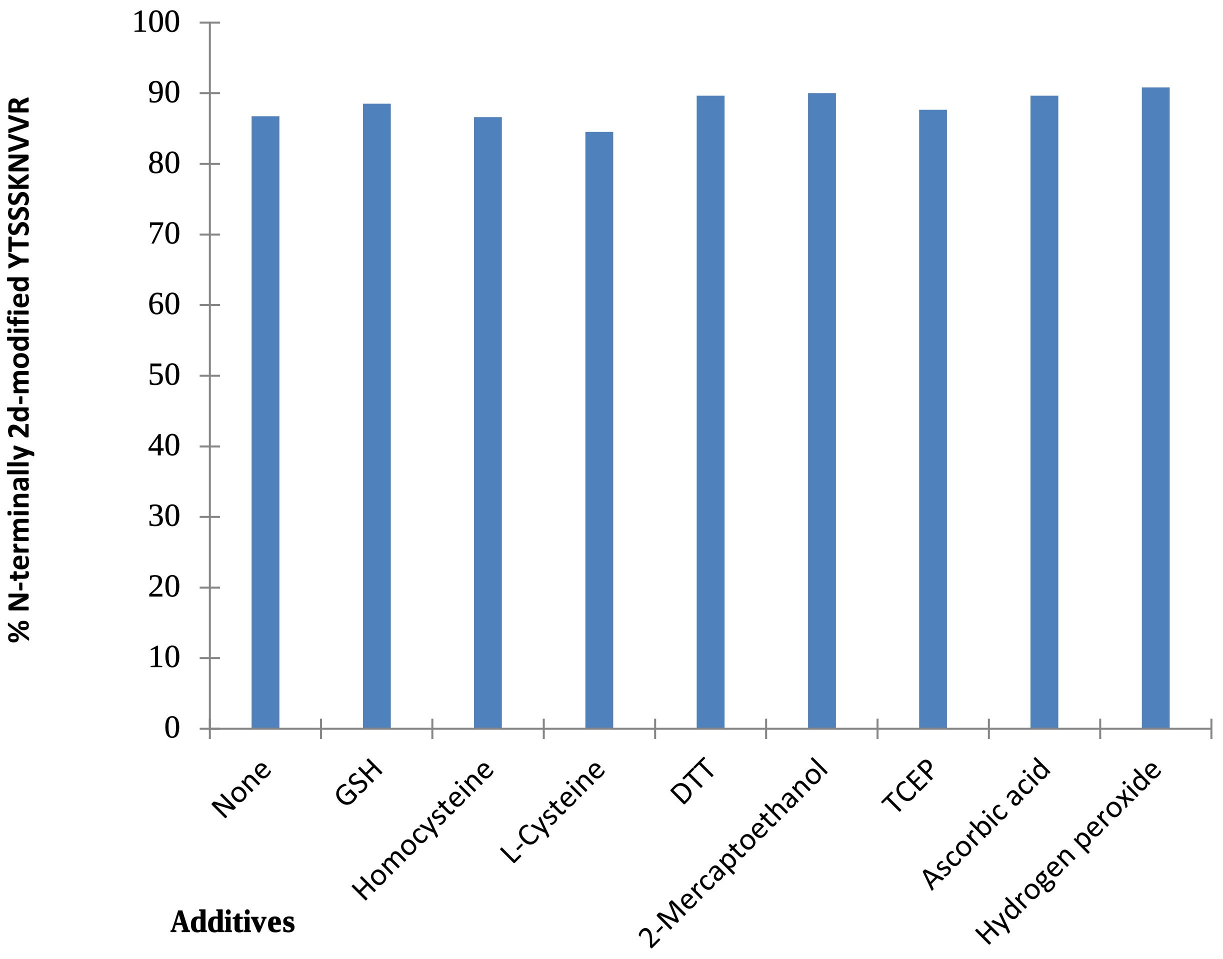

Supplementary Fig. 49 Stability studies of **2d**-modified YTSSSKNVVR.

General procedure for modification of XSKFR (X = either one of 20 natural amino acid) using 2-ethynyl-5-hydroxy-4-methoxybenzaldehyde (2d)

To a 1.5 mL eppendorf tube with 80 µL of 50 mM PBS buffer (pH 6.5), 10 µL of XSKFR (1 mM in Milli-Q® water) was added, followed by 10 µL of 2-EBA **2d** (20 mM in DMSO). The reactive mixture was allowed to react in a 37 ^{o}C water bath for 16 h. 20 µL of the mixture was drawn, diluted with 20 µL Milli-Q® and subjected to LC-MS and LC-MS/MS analysis.

Modification of Peptide XSKFR using 2-Ethynyl-5-hydroxy-4-methoxybenzaldehyde 2d

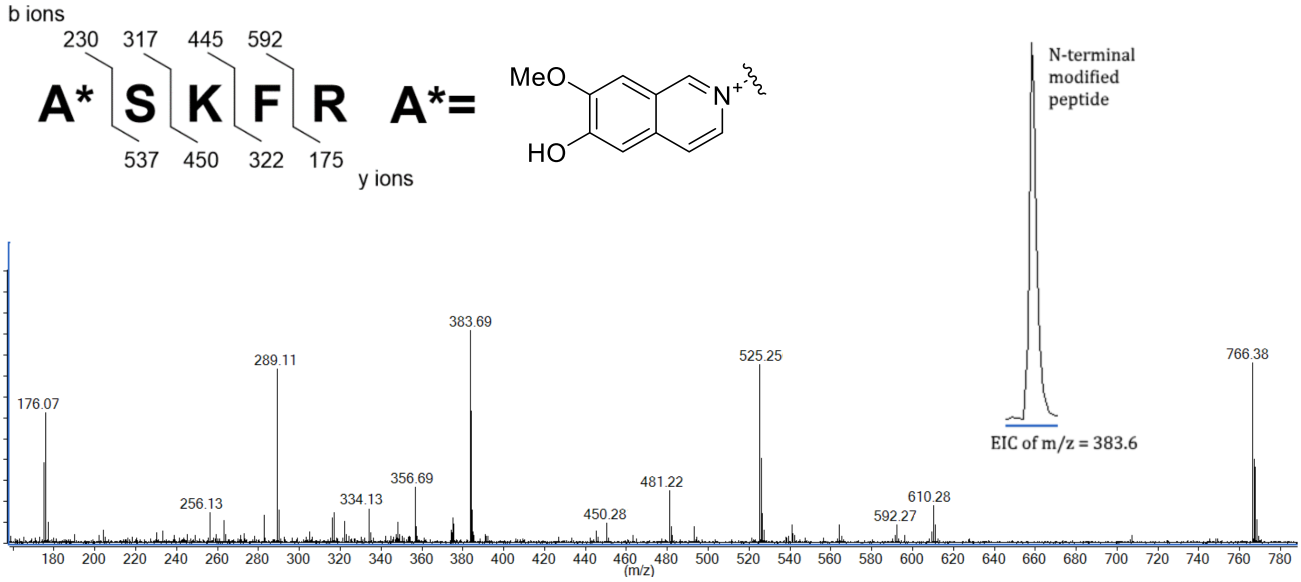

Supplementary Fig. 50 Q-TOF MS/MS spectrum of N-terminal **2d**-modified ASKFR (ESI source, doubly charged ion of m/z = 383.6). Inset: the extracted ion chromatogram (EIC) of doubly charged ion of *m/z* = 383.6.

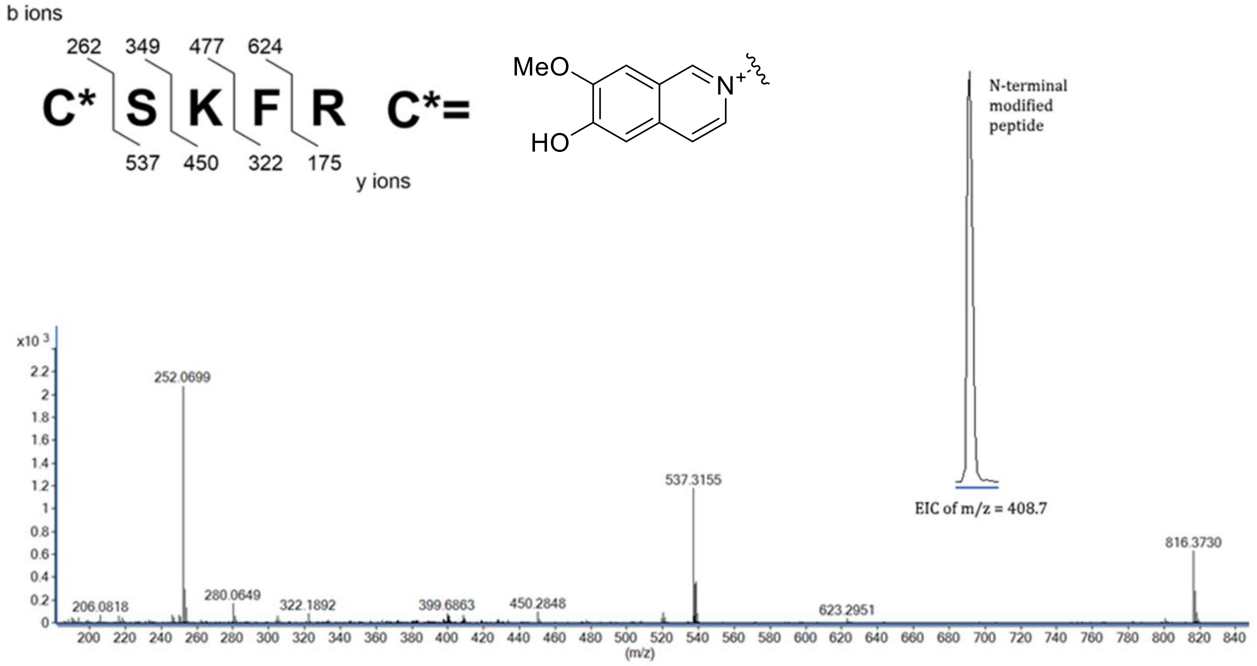

Supplementary Fig. 51 Q-TOF MS/MS spectrum of N-terminal **2d**-modified CSKFR hydrate (ESI source, doubly charged ion of *m/z* = 408.7). Inset: the extracted ion chromatogram (EIC) of doubly charged ion of *m/z* = 408.7.

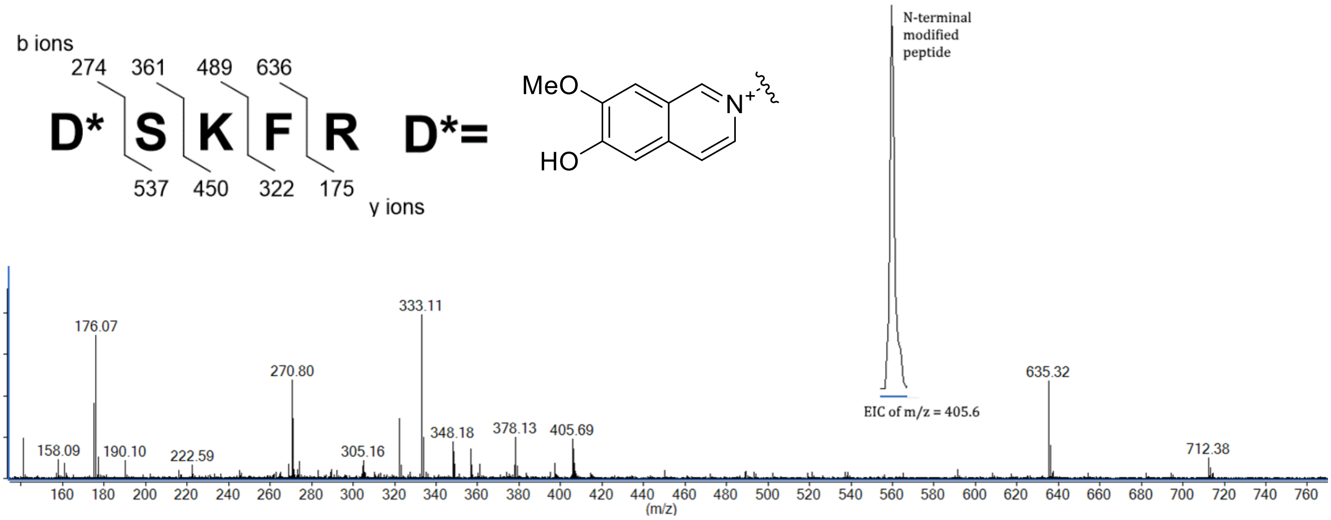

Supplementary Fig. 52 Q-TOF MS/MS spectrum of N-terminal **2d**-modified DSKFR (ESI source, doubly charged ion of *m/z* = 405.6). Inset: the extracted ion chromatogram (EIC) of doubly charged ion of *m/z* = 405.6.

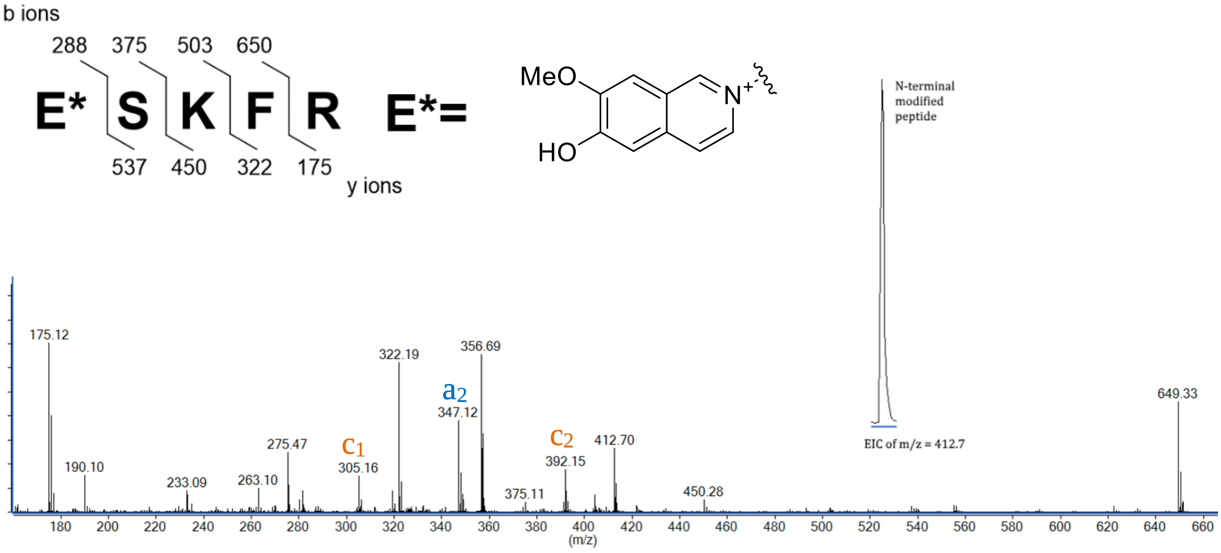

Supplementary Fig. 53 Q-TOF MS/MS spectrum of N-terminal **2d**-modified ESKFR (ESI source, doubly charged ion of *m/z* = 412.7). Inset: the extracted ion chromatogram (EIC) of doubly charged ion of *m/z* = 412.7.

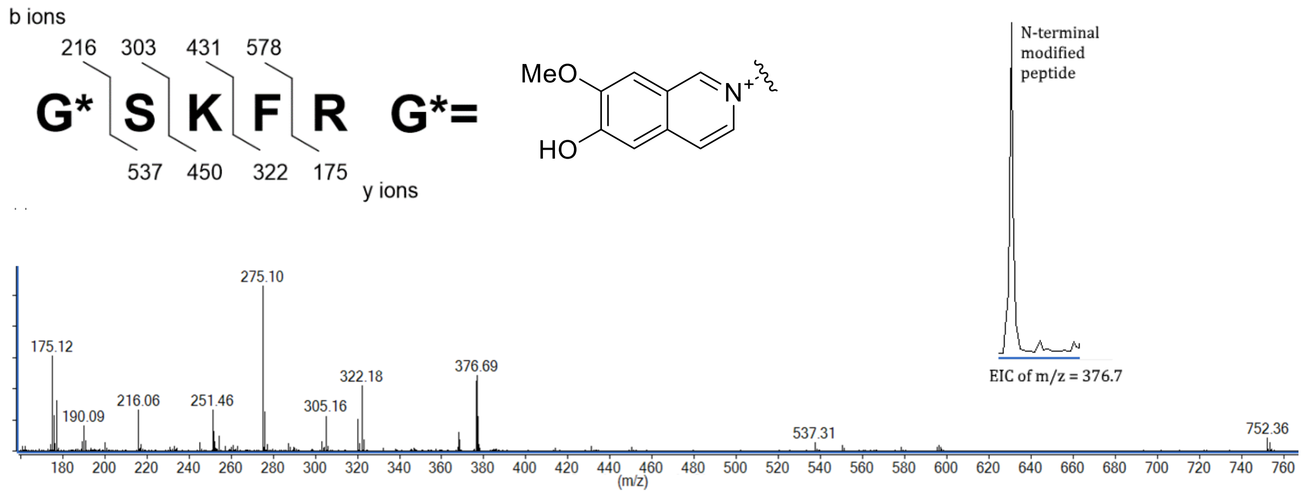

Supplementary Fig. 54 Q-TOF MS/MS spectrum of N-terminal **2d**-modified GSKFR (ESI source, doubly charged ion of *m/z* = 376.7). Inset: the extracted ion chromatogram (EIC) of doubly charged ion of *m/z* = 376.7.

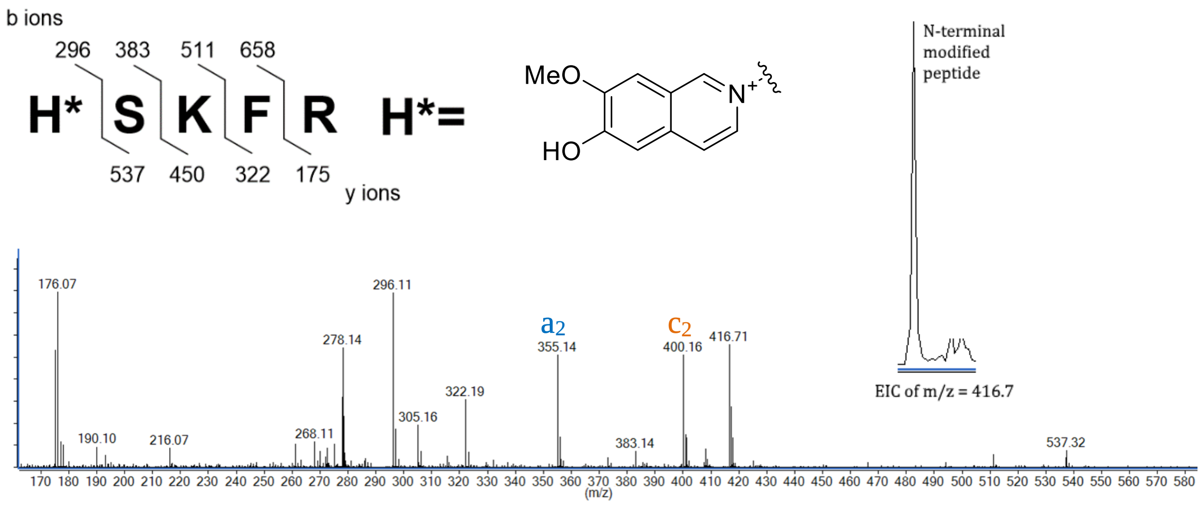

Supplementary Fig. 55 Q-TOF MS/MS spectrum of N-terminal **2d**-modified HSKFR (ESI source, doubly charged ion of *m/z* = 416.7). Inset: the extracted ion chromatogram (EIC) of doubly charged ion of *m/z* = 416.7.

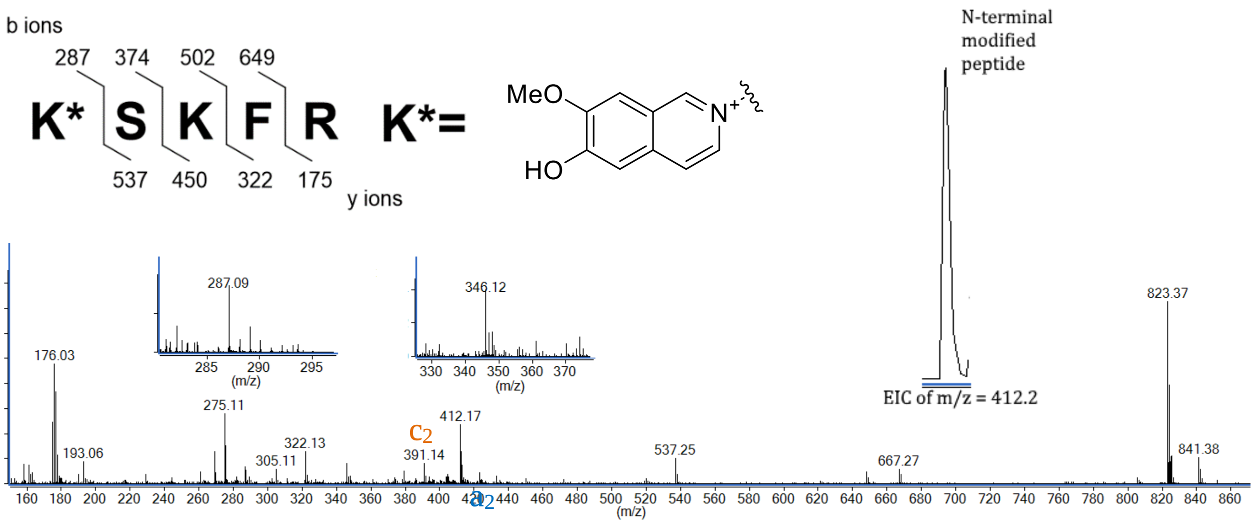

Supplementary Fig. 56 Q-TOF MS/MS spectrum of N-terminal **2d**-modified KSKFR (ESI source, doubly charged ion of *m/z* = 412.2). Inset: the extracted ion chromatogram (EIC) of doubly charged ion of *m/z* = 412.2.

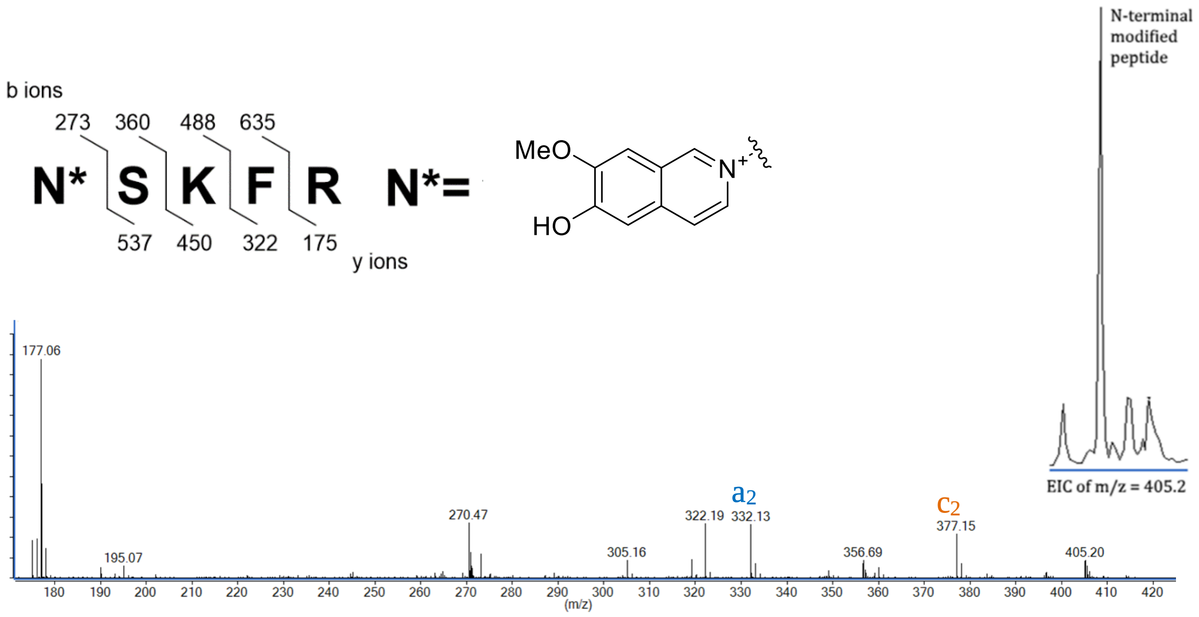

Supplementary Fig. 57 Q-TOF MS/MS spectrum of N-terminal **2d**-modified NSKFR (ESI source, doubly charged ion of *m/z* = 405.2). Inset: the extracted ion chromatogram (EIC) of doubly charged ion of *m/z* = 405.2.

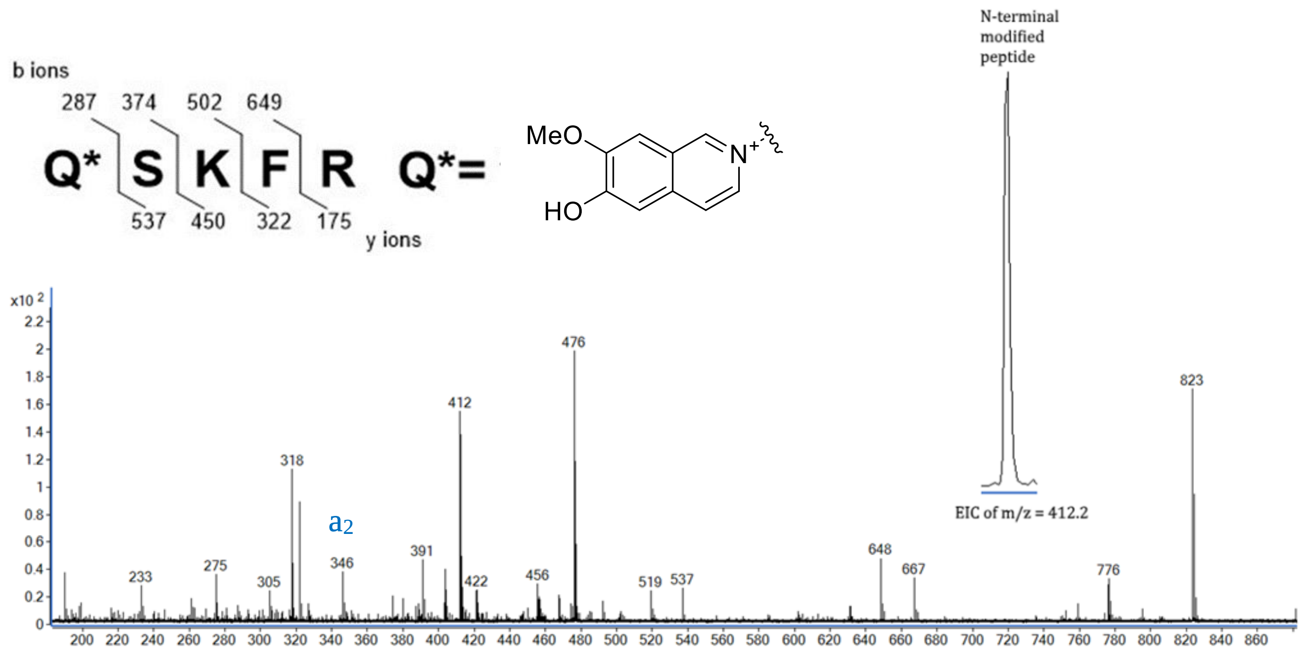

Supplementary Fig. 58 Q-TOF MS/MS spectrum of N-terminal **2d**-modified QSK**FR** (ESI source, doubly charged ion of *m/z* = 412.2). Inset: the extracted ion chromatogram (EIC) of doubly charged ion of *m/z* = 412.2.

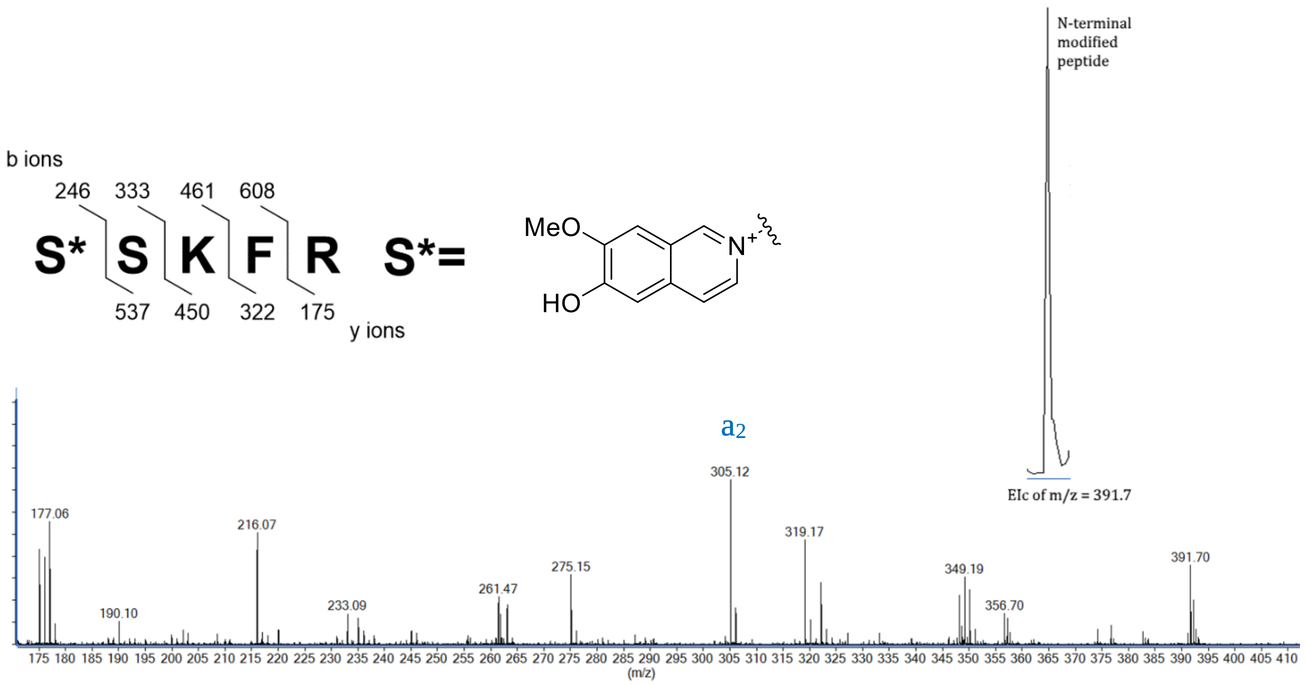

Supplementary Fig. 59 Q-TOF MS/MS spectrum of N-terminal **2d**-modified SSKFR (ESI source, doubly charged ion of *m/z* = 391.7). Inset: the extracted ion chromatogram (EIC) of doubly charged ion of *m/z* = 391.7.

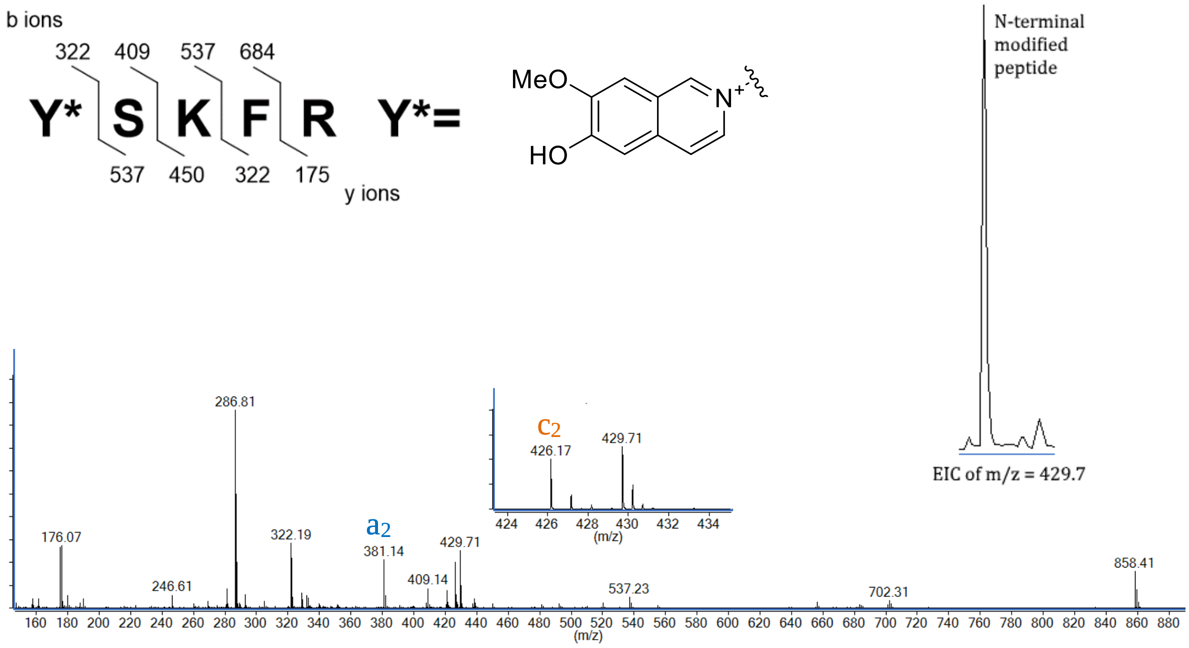

Supplementary Fig. 60 Q-TOF MS/MS spectrum of N-terminal **2d**-modified YSKFR (ESI source, doubly charged ion of *m/z* = 429.7). Inset: the extracted ion chromatogram (EIC) of doubly charged ion of *m/z* = 429.7.

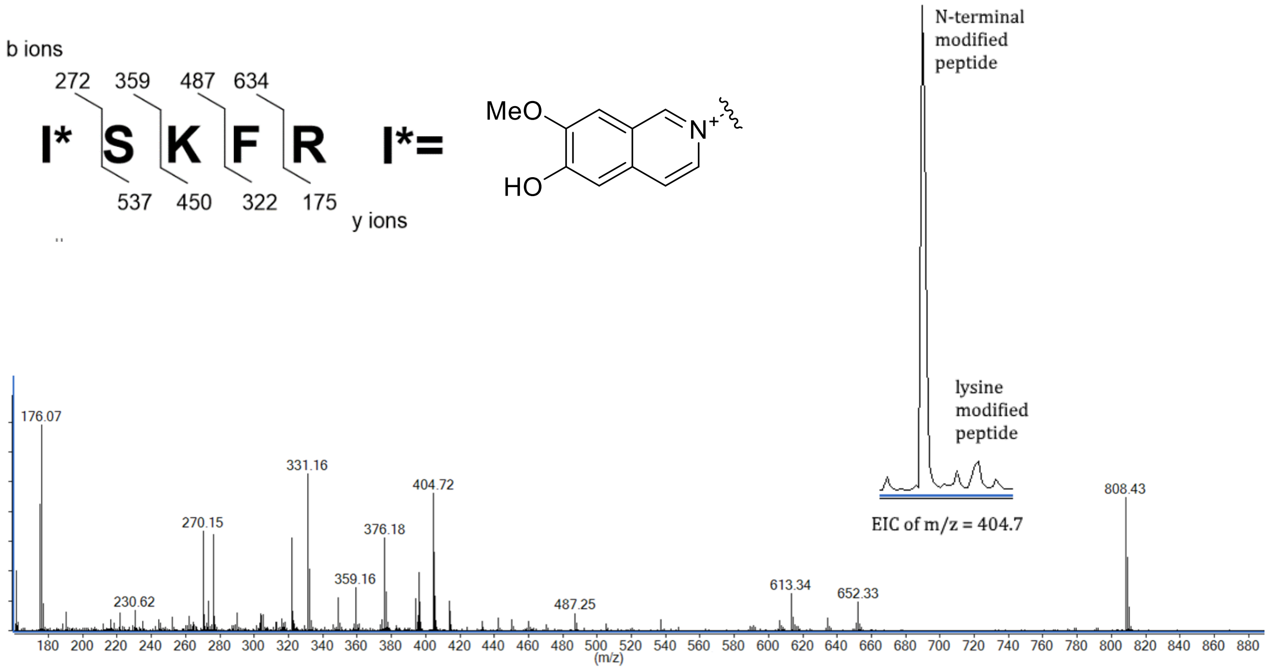

Supplementary Fig. 61 Q-TOF MS/MS spectrum of N-terminal **2d**-modified (ESI source, doubly charged ion of *m/z* = 404.7). Inset: the extracted ion chromatogram (EIC) of doubly charged ion of *m/z* = 404.7.

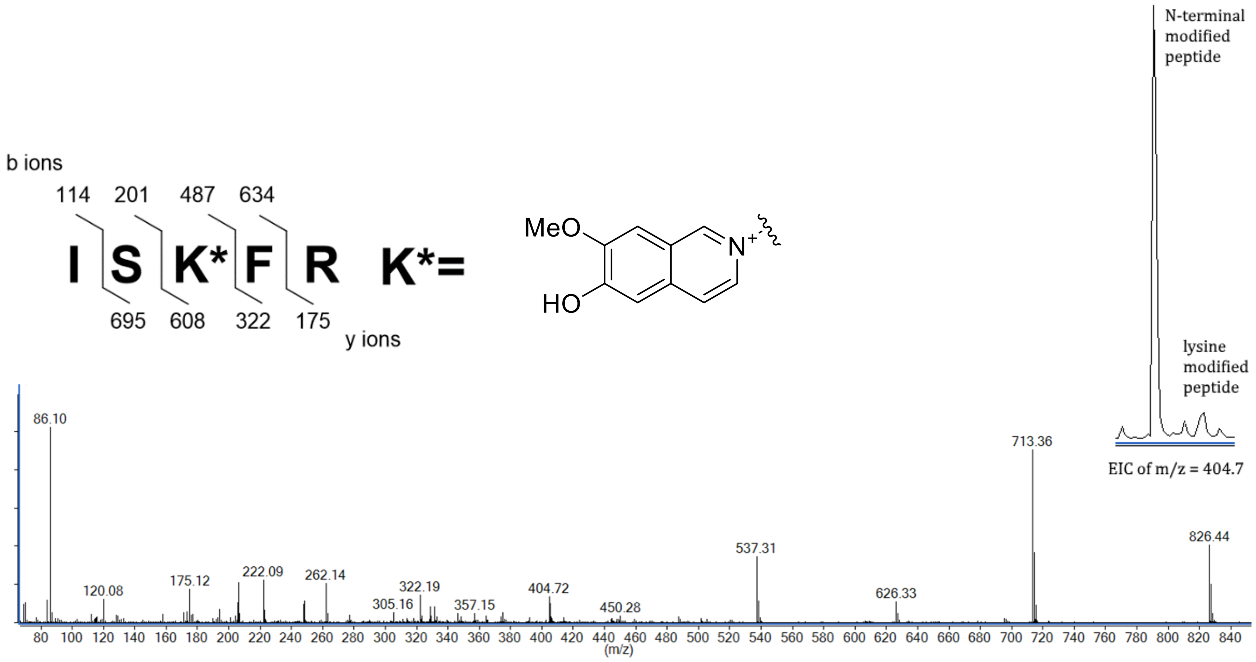

Supplementary Fig. 62 Q-TOF MS/MS spectrum of lysine **2d**-modified ISKFR (ESI source, doubly charged ion of *m/z* = 404.7). Inset: the extracted ion chromatogram (EIC) of doubly charged ion of *m/z* = 404.7.

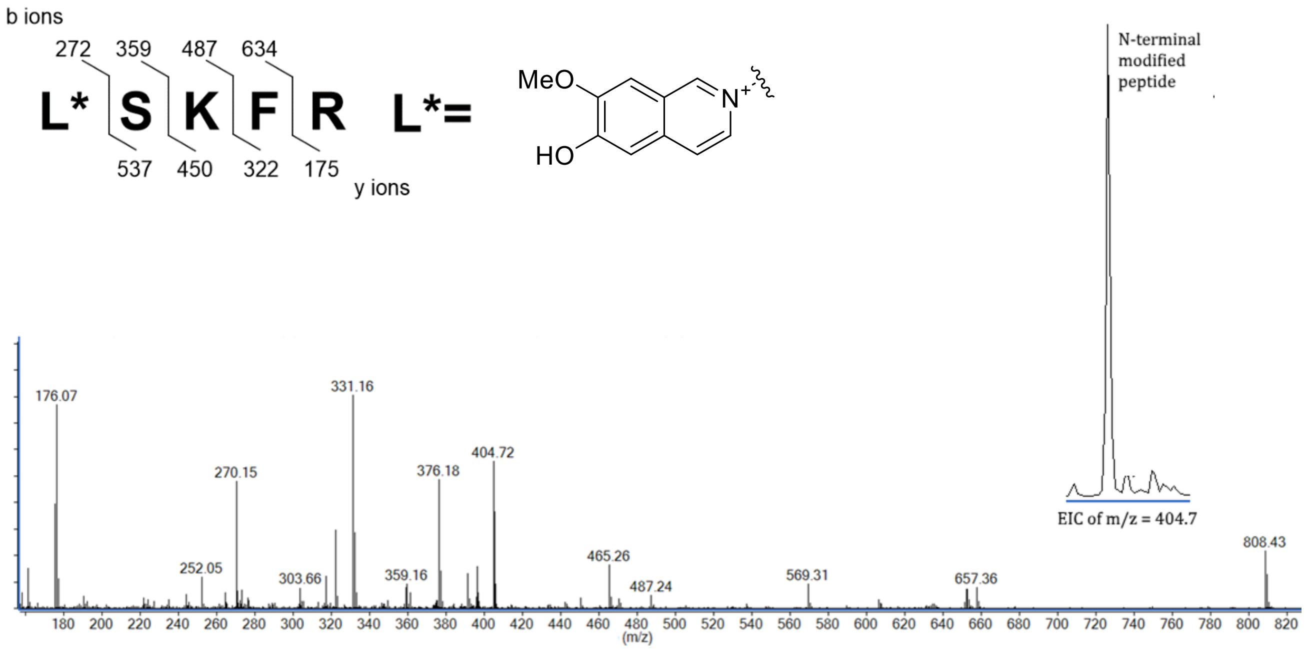

Supplementary Fig. 63 Q-TOF MS/MS spectrum of N-terminal **2d**-modified LSKFR (ESI source, doubly charged ion of *m/z* = 404.7). Inset: the extracted ion chromatogram (EIC) of doubly charged ion of *m/z* = 404.7.

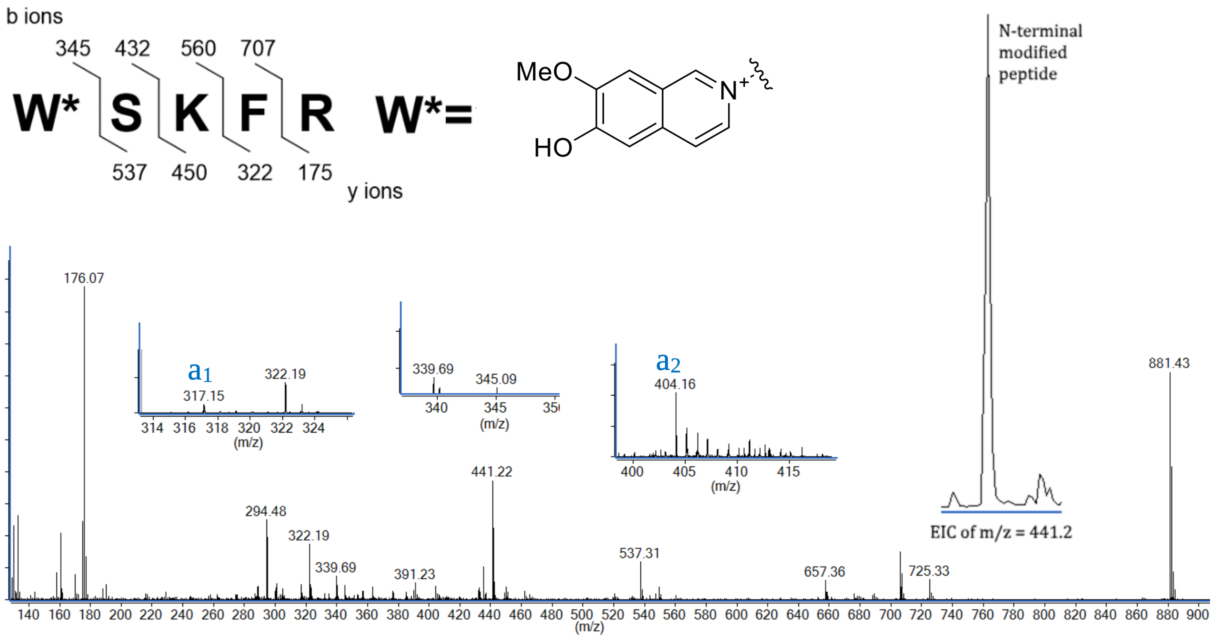

Supplementary Fig. 64 Q-TOF MS/MS spectrum of N-terminal **2d**-modified WSKFR (ESI source, doubly charged ion of *m/z* = 441.2). Inset: the extracted ion chromatogram (EIC) of doubly charged ion of *m/z* = 441.2.

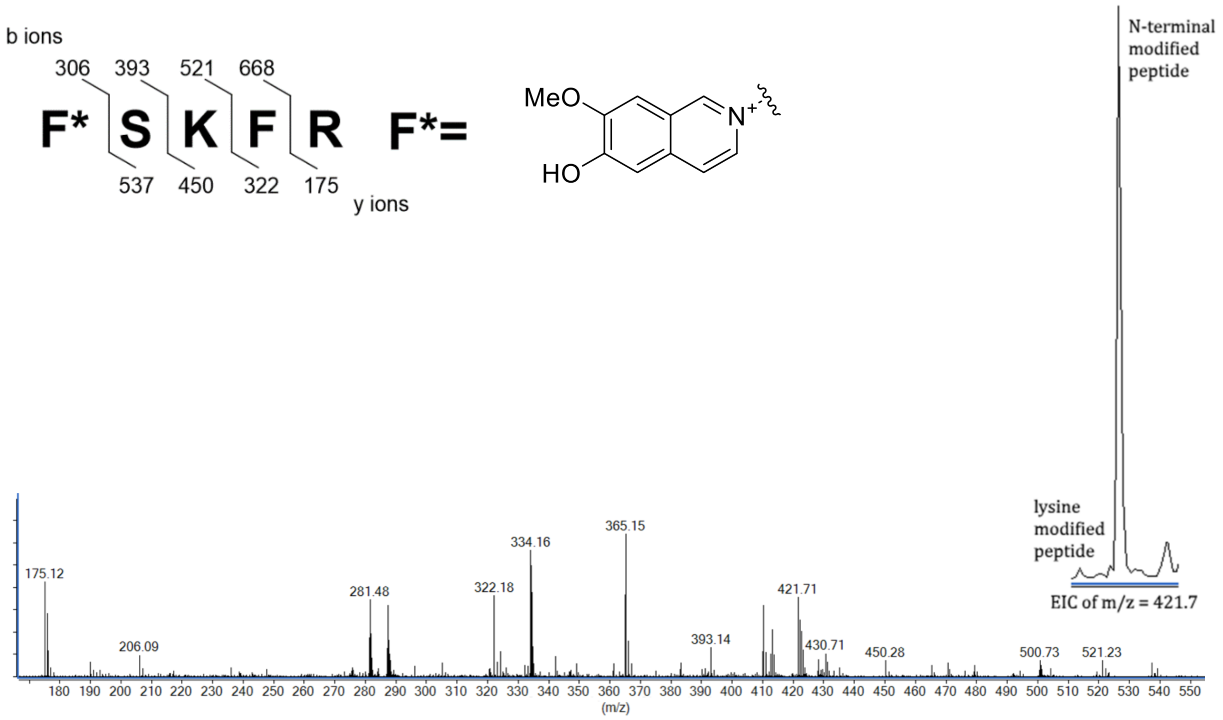

Supplementary Fig. 65 Q-TOF MS/MS spectrum of N-terminal **2d**-modified FSKFR (ESI source, doubly charged ion of *m/z* = 421.7). Inset: the extracted ion chromatogram (EIC) of doubly charged ion of *m/z* = 421.7.

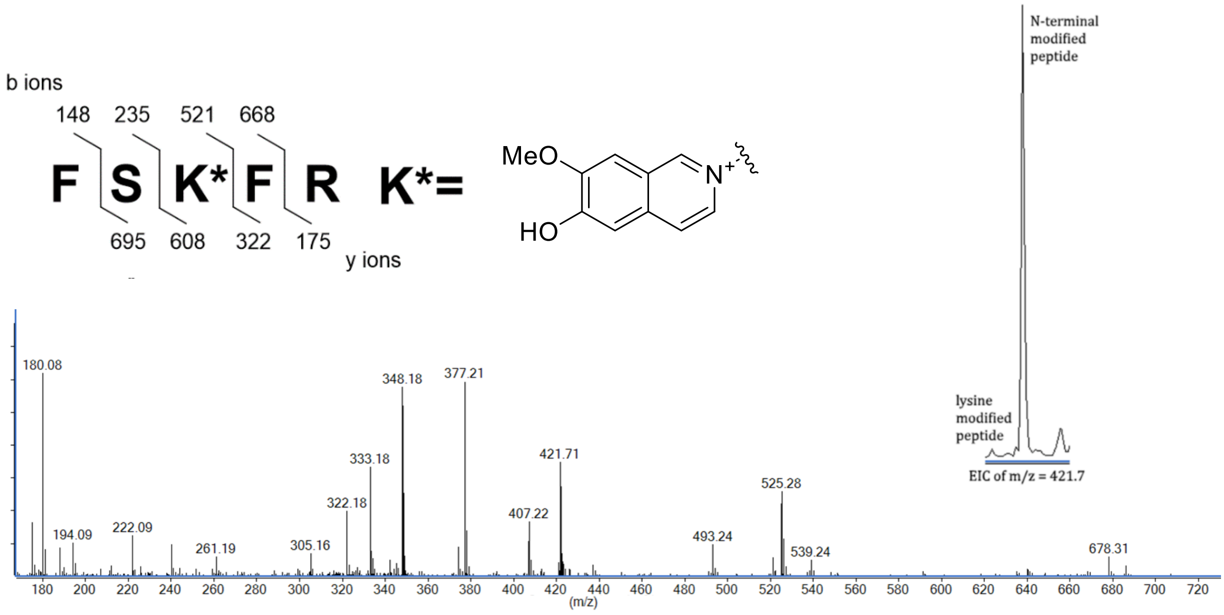

Supplementary Fig. 66 Q-TOF MS/MS spectrum of lysine **2d**-modified FSKFR (ESI source, doubly charged ion of *m/z* = 421.7). Inset: the extracted ion chromatogram (EIC) of doubly charged ion of *m/z* = 421.7.

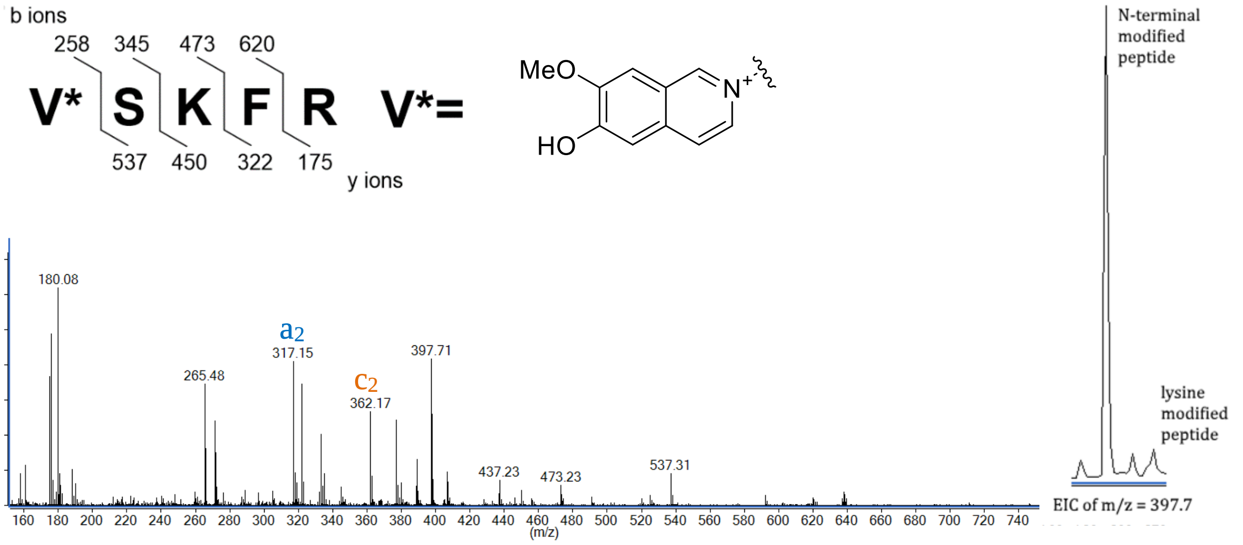

Supplementary Fig. 67 Q-TOF MS/MS spectrum of N-terminal **2d**-modified VSKFR (ESI source, doubly charged ion of *m/z* = 397.7). Inset: the extracted ion chromatogram (EIC) of doubly charged ion of *m/z* = 397.7.

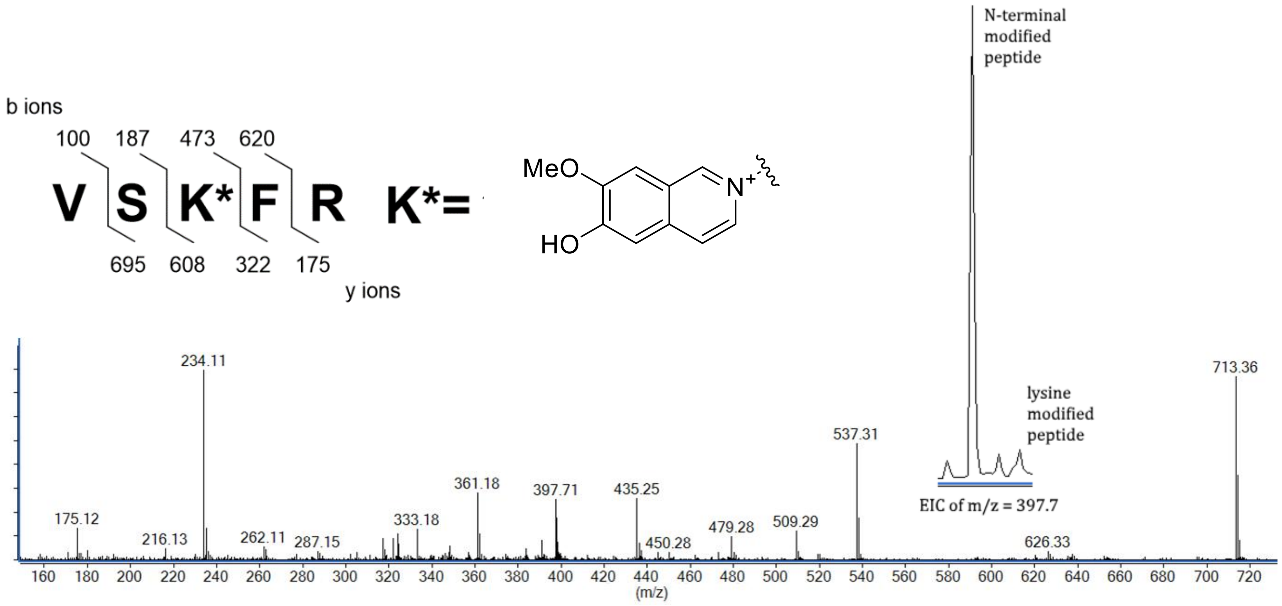

Supplementary Fig. 68 Q-TOF MS/MS spectrum of lysine **2d**-modified VSKFR (ESI source, doubly charged ion of *m/z* = 397.7). Inset: the extracted ion chromatogram (EIC) of doubly charged ion of *m/z* = 397.7.

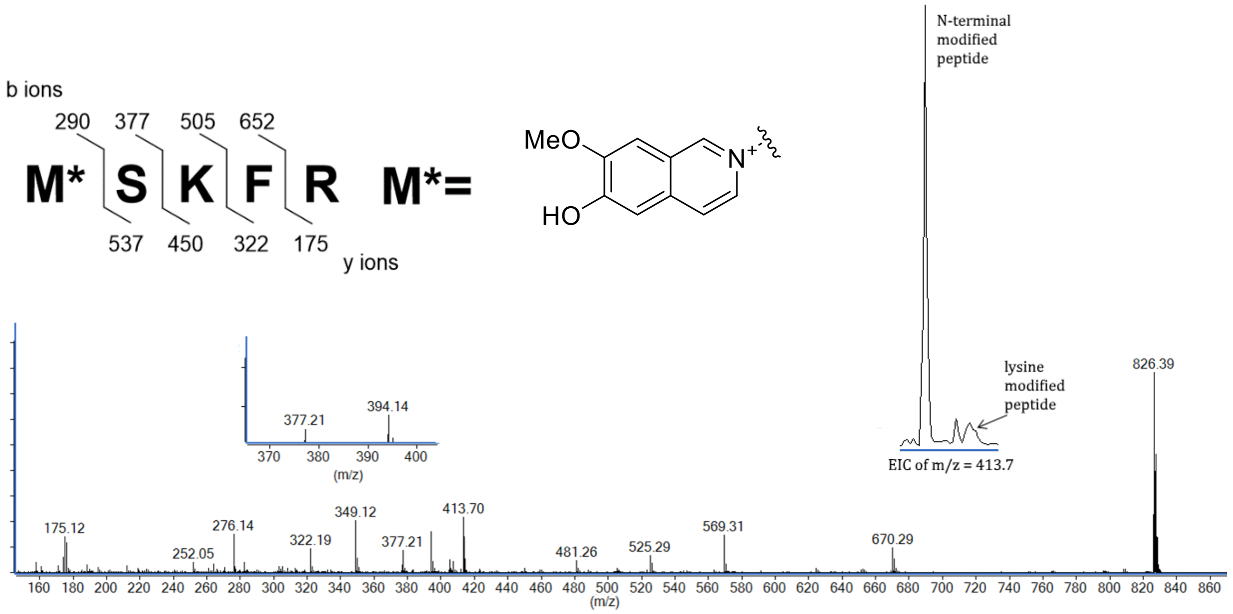

Supplementary Fig. 69 Q-TOF MS/MS spectrum of N-terminal **2d**-modified MSKFR (ESI source, doubly charged ion of *m/z* = 413.7). Inset: the extracted ion chromatogram (EIC) of doubly charged ion of *m/z* = 413.7.

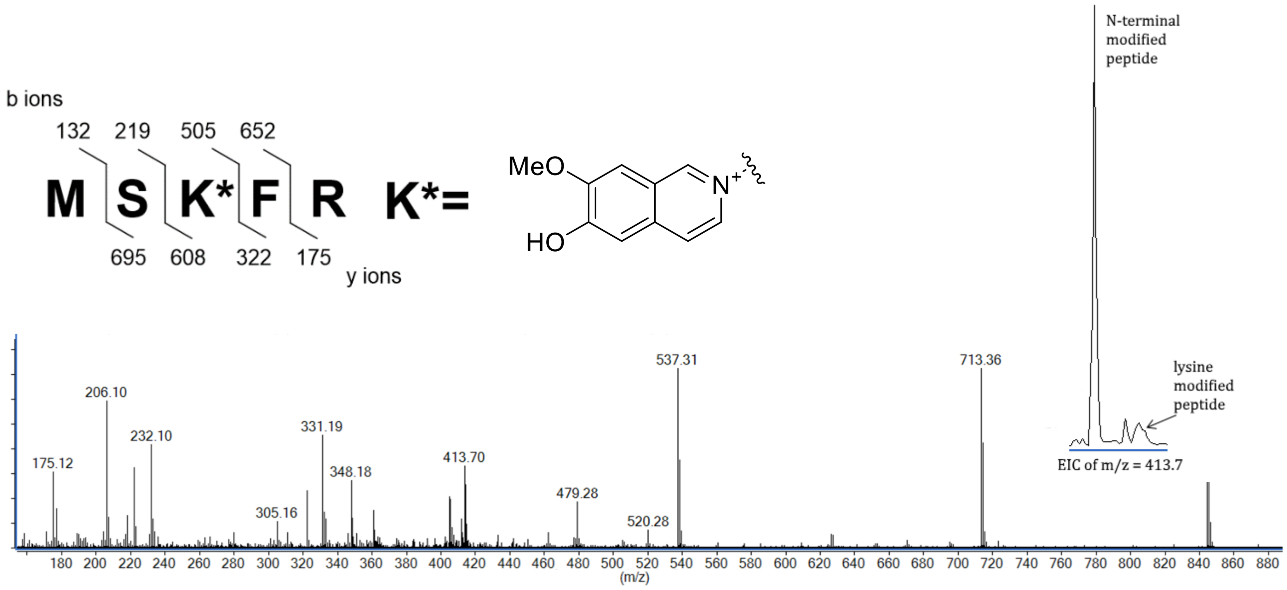

Supplementary Fig. 70 Q-TOF MS/MS spectrum of lysine **2d**-modified MSKFR (ESI source, doubly charged ion of *m/z* = 413.7). Inset: the extracted ion chromatogram (EIC) of doubly charged ion of m/z = 413.7.

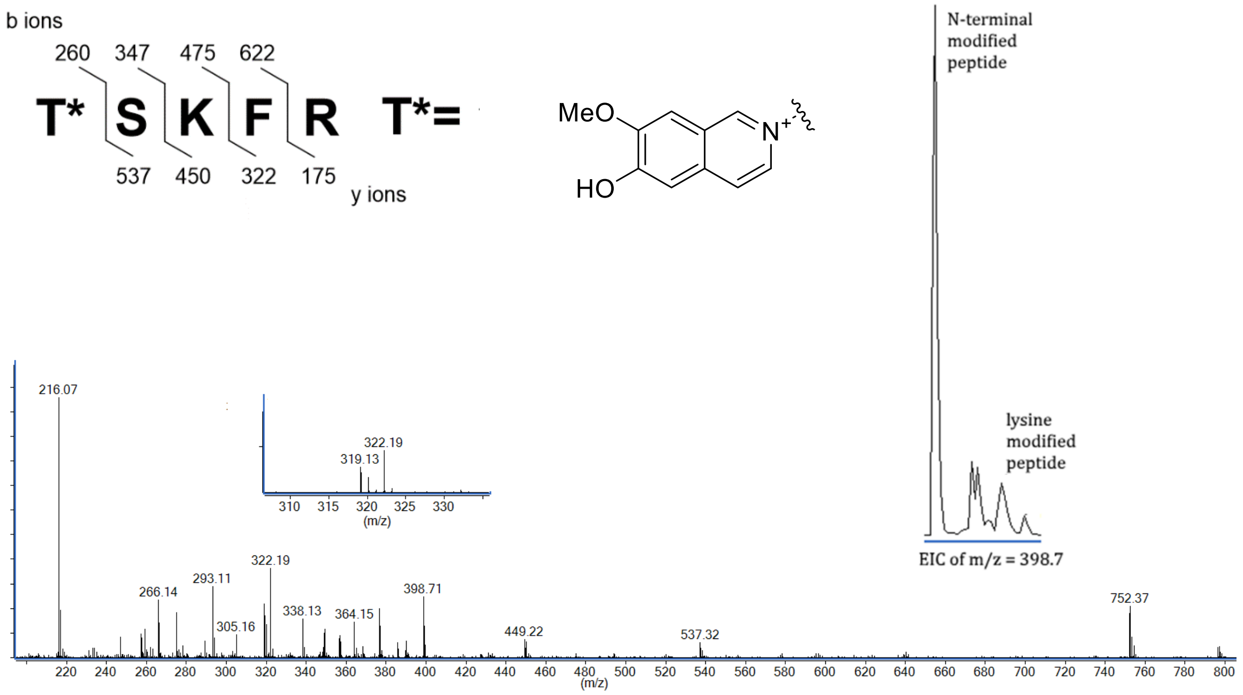

Supplementary Fig. 71 Q-TOF MS/MS spectrum of N-terminal **2d-**modified TSKFR (ESI source, doubly charged ion of *m/z* = 398.7). Inset: the extracted ion chromatogram (EIC) of doubly charged ion of *m/z* = 398.7.

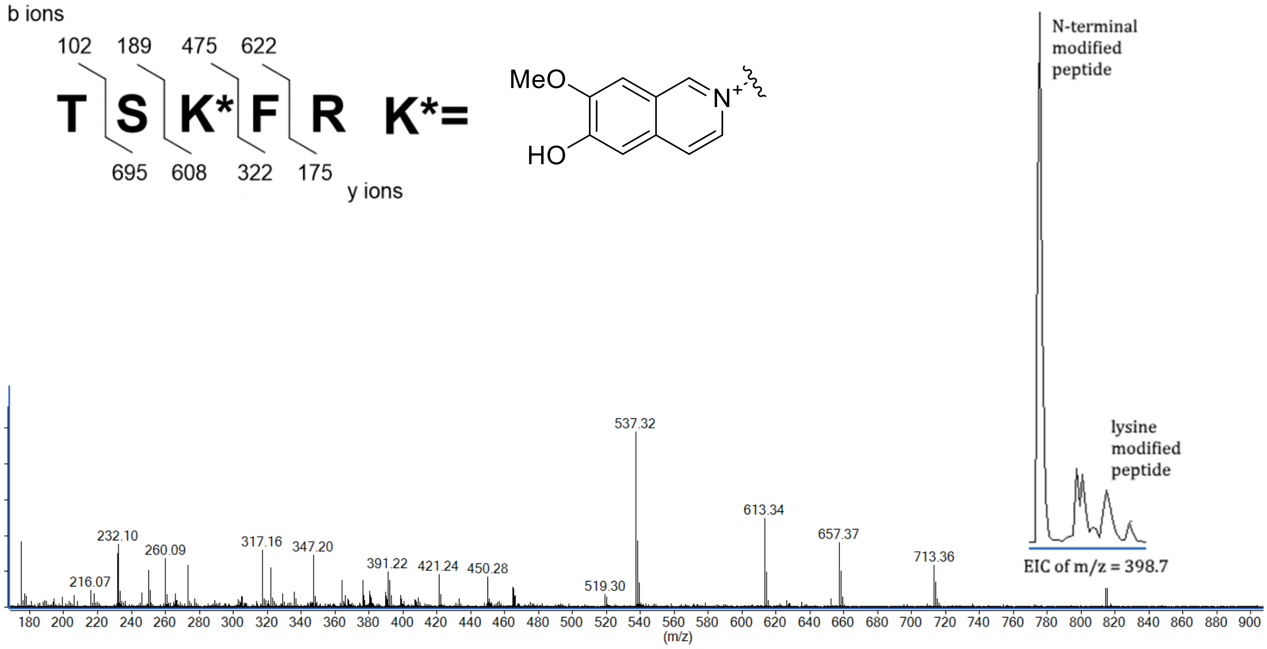

Supplementary Fig. 72 Q-TOF MS/MS spectrum of lysine **2d**-modified TSKFR (ESI source, doubly charged ion of *m/z* = 398.7). Inset: the extracted ion chromatogram (EIC) of doubly charged ion of *m/z* = 398.7.

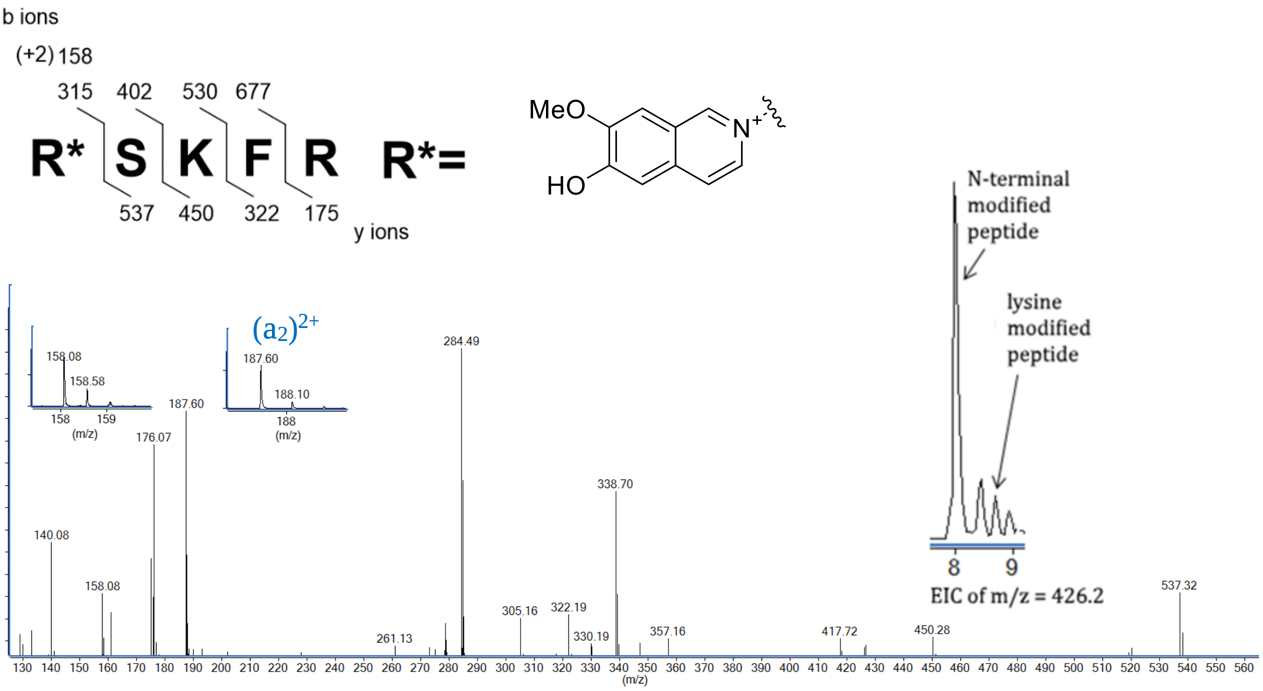

Supplementary Fig. 73 Q-TOF MS/MS spectrum of N-terminal **2d**-modified RSKFR (ESI source, doubly charged ion of *m/z* = 426.2). Inset: the extracted ion chromatogram (EIC) of doubly charged ion of *m/z* = 426.2.

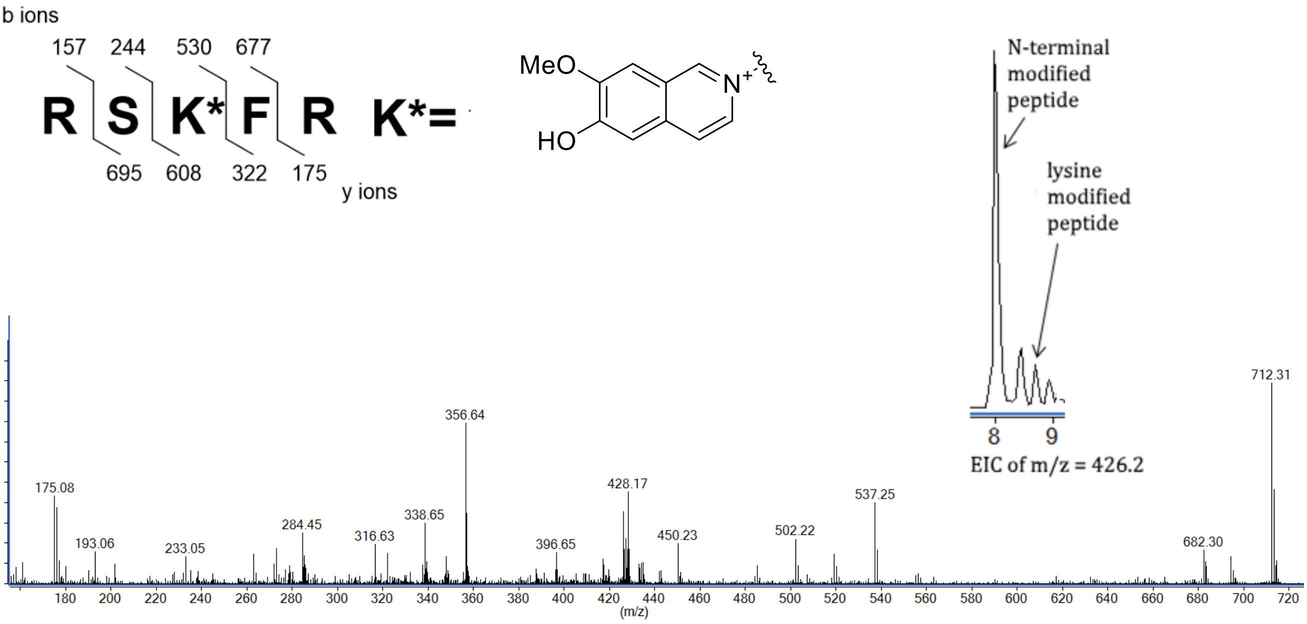

Supplementary Fig. 74 Q-TOF MS/MS spectrum of lysine **2d**-modified RSKFR (ESI source, doubly charged ion of *m/z* = 426.2). Inset: the extracted ion chromatogram (EIC) of doubly charged ion of *m/z* = 426.2.

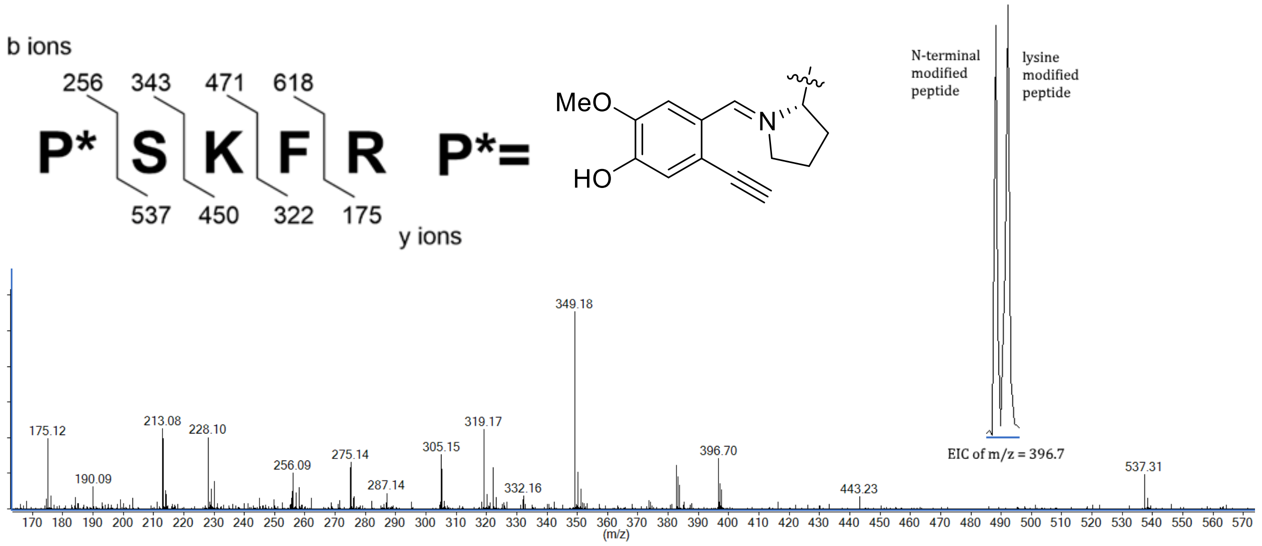

Supplementary Fig. 75 Q-TOF MS/MS spectrum of N-terminal **2d**-modified PSKFR (ESI source, doubly charged ion of *m/z* = 396.7). Inset: the extracted ion chromatogram (EIC) of doubly charged ion of *m/z* = 396.7.

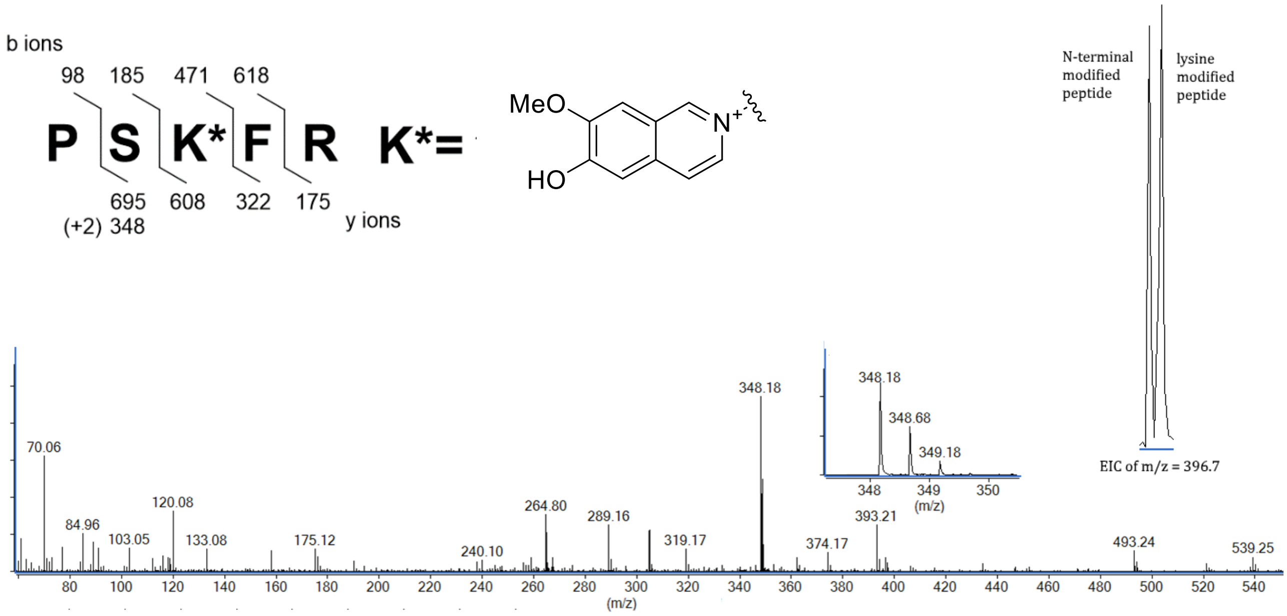

Supplementary Fig. 76 Q-TOF MS/MS spectrum of lysine **2d**-modified PSKFR (ESI source, doubly charged ion of *m/z* = 396.7). Inset: the extracted ion chromatogram (EIC) of doubly charged ion of *m/z* = 396.7.

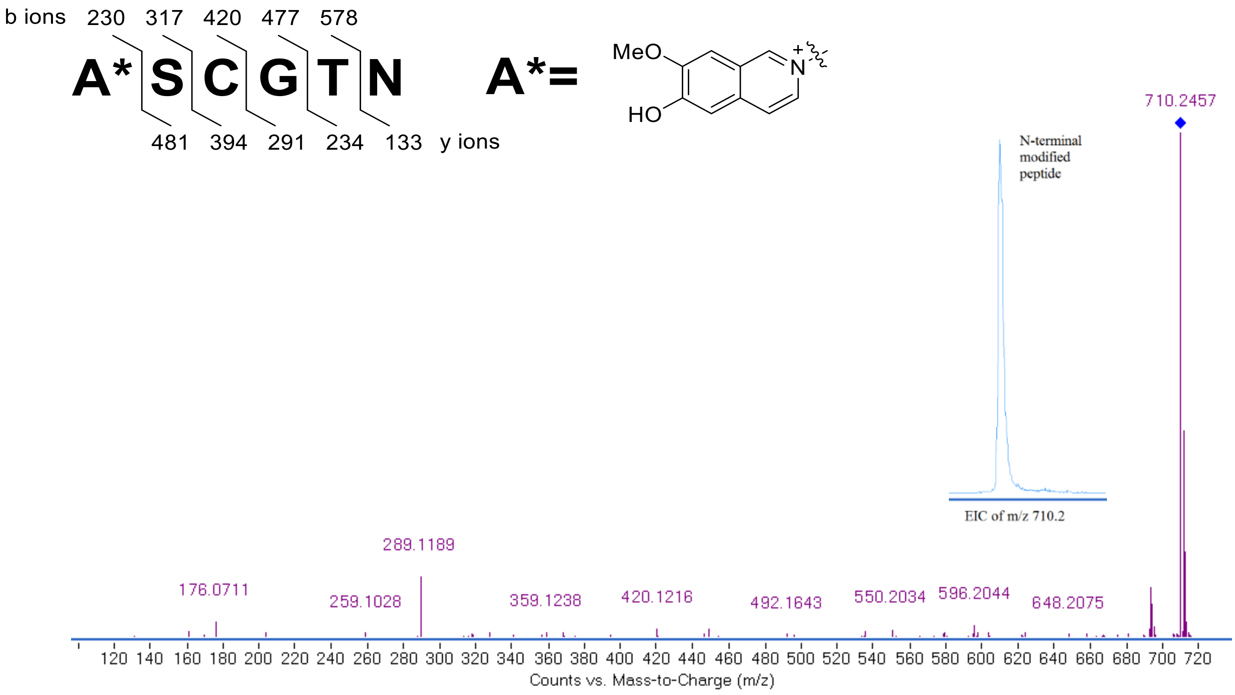

Supplementary Fig. 77 Q-TOF MS/MS spectrum of N-terminal **2d**-modified ASCGTN (ESI source, singly charged ion of *m/z* = 710.2). Inset: the extracted ion chromatogram (EIC) of singly charged ion of *m/z* = 710.2.

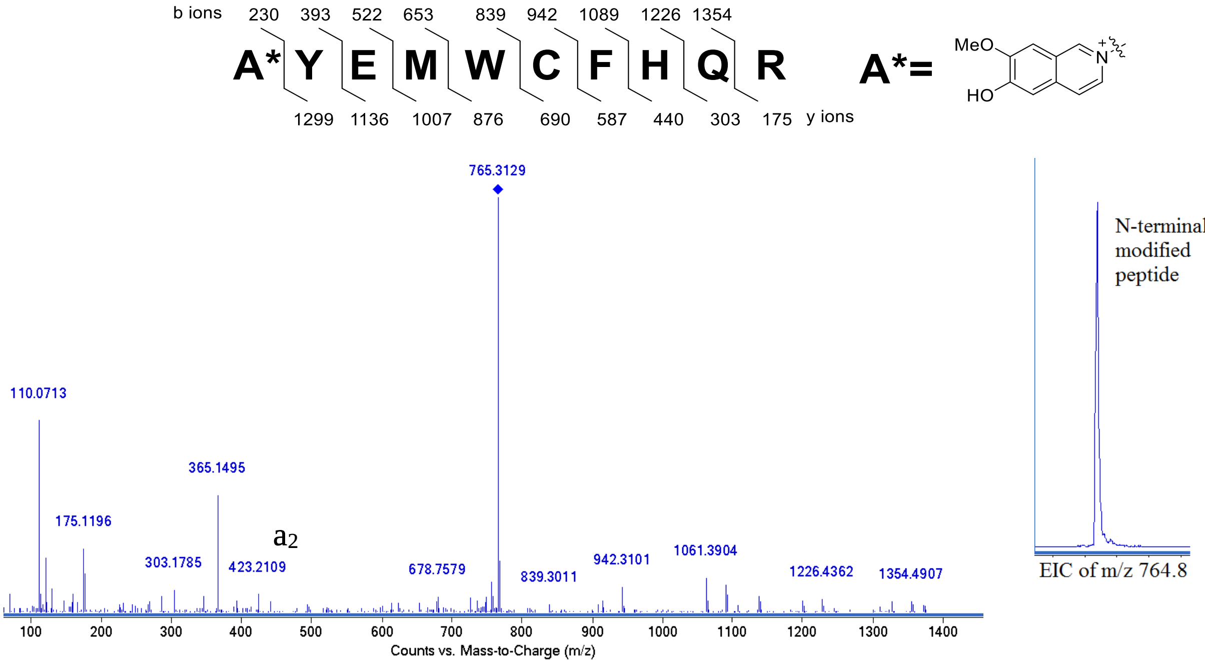

Supplementary Fig. 78 Q-TOF MS/MS spectrum of N-terminal **2d**-modified AYEMWCFHQK (ESI source, doubly charged ion of m/z = 750.7). Inset: the extracted ion chromatogram (EIC) of doubly charged ion of *m/z* = 750.7.

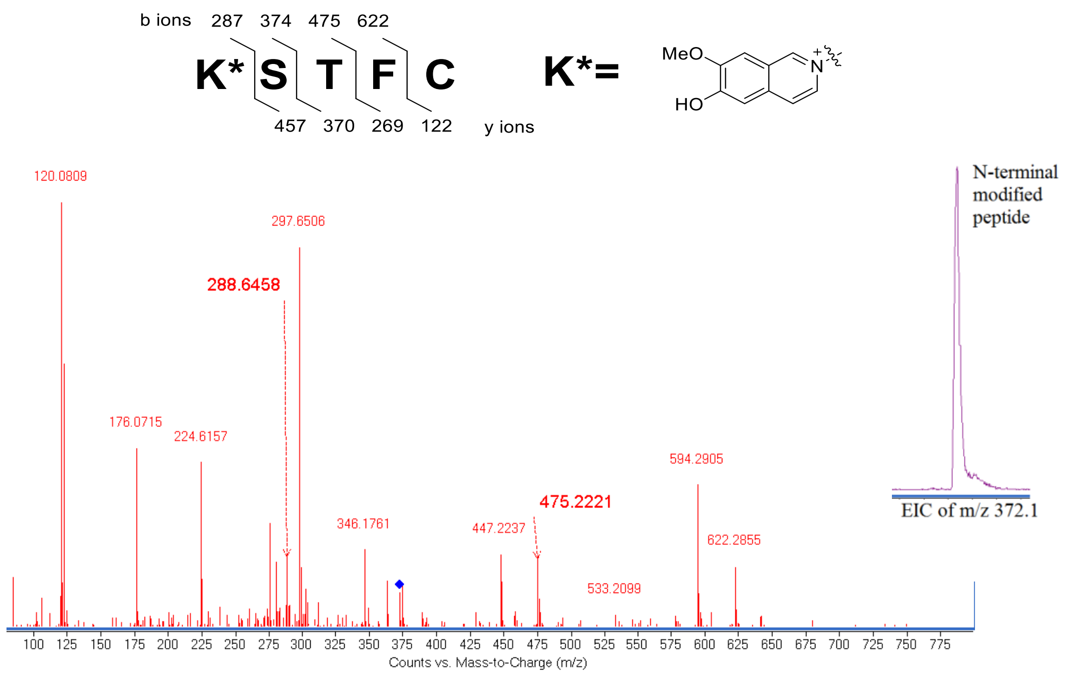

Supplementary Fig. 79 Q-TOF MS/MS spectrum of N-terminal **2d**-modified KSTFC (ESI source, doubly charged ion of *m/z* = 372.1). Inset: the extracted ion chromatogram (EIC) of doubly charged ion of *m/z* = 372.1.

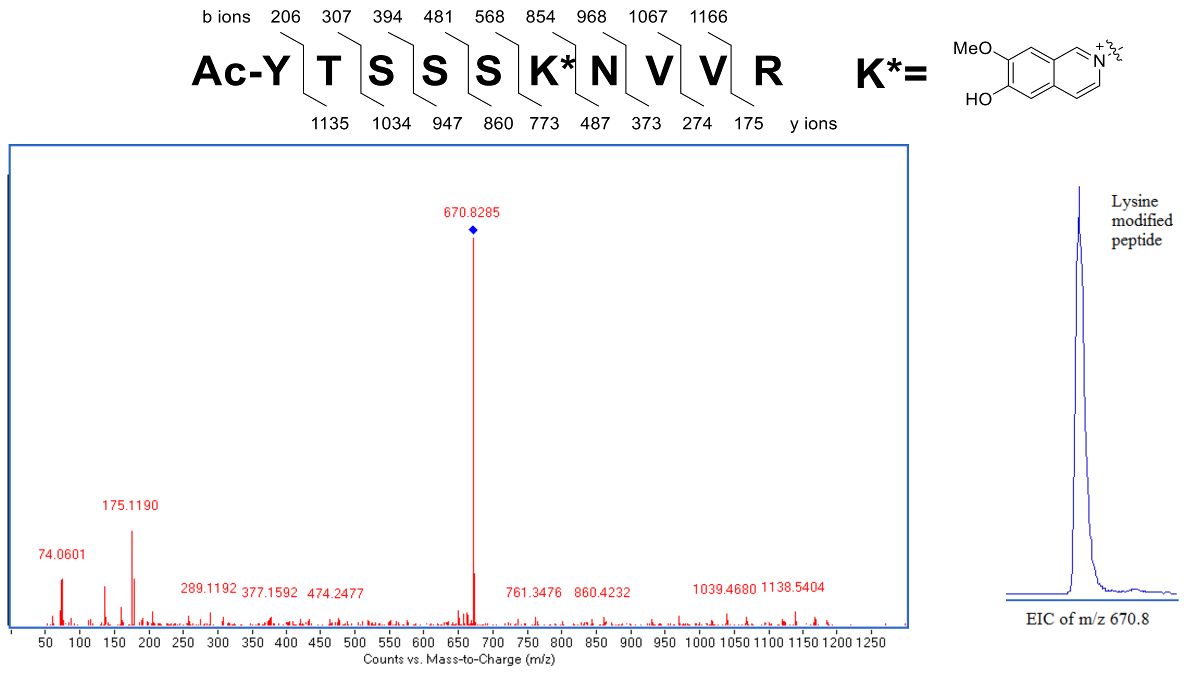

Supplementary Fig. 80 Q-TOF MS/MS spectrum of lysine **2d**-modified Ac-YTSSSKNVVR (ESI source, doubly charged ion of *m/z* = 670.8). Inset: the extracted ion chromatogram (EIC) of doubly charged ion of *m/z* = 670.8.

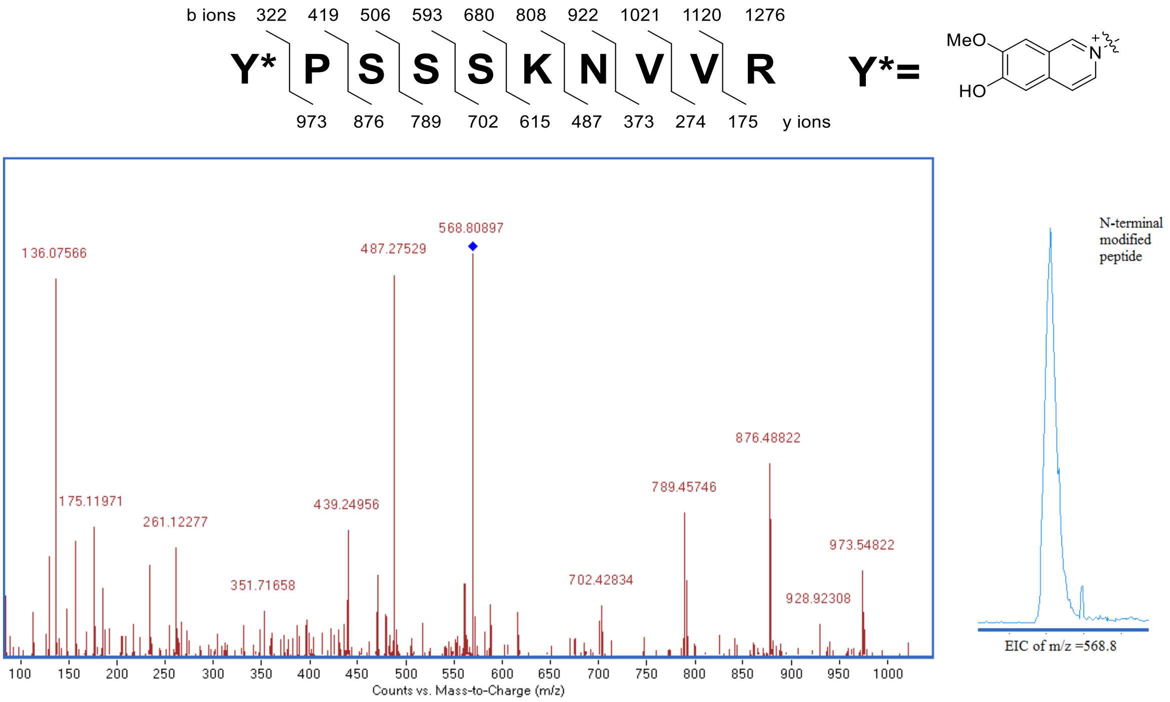

Supplementary Fig. 81 Q-TOF MS/MS spectrum of N-terminal **2d**-modified YPSSSKNVVR (ESI source, doubly charged ion of m/z = 568.8). Inset: the extracted ion chromatogram (EIC) of doubly charged ion of m/z = 568.8.

Modification of Proteins Using Functionalized 2-Ethynylbenzaldehydes (2t and 2u)

A mixture of 10 µL of proteins (lysozyme, RNaseA or insulin) (1 mM in 50 mM pH 7.4 PBS buffer), 5 µL of functionalized 2-ethynylbenzaldehyde (**2t** or **2u**, 10 mM in DMSO), 5 µL of DMSO and 80 µL of 50 mM pH 7.4 phosphate-buffered saline was treated in a 1.5 mL Eppendorf tube at 37 ^{o}C for 16 h. The modified proteins were purified by Bio-Rad Bio-Spin® 6 column prior to LC-MS analysis and trypsin digestion.

For modification of the therapeutic protein BCArg mutant, a mixture of 2 mL of BCArg mutant (0.15 mM in 50 mM pH 7.4 phosphate-buffered saline), 300 µL of functionalized 2-ethynylbenzaldehyde (**2t** or **2u**, 10 mM in DMSO) and 1.7 mL of 50 mM pH 7.4 phosphate-buffered saline was treated in a 15 mL centrifuge tube at 37 ^{o}C for 16 h. The modified product was characterized by LC-MS analysis.

Before sequential modification, and analysis of biological properties, the modified proteins in solution were added into the filter of a Millipore Amicon® Ultra-4 or -15 10K centrifugal device. After that, the filtrate was filled with 50 mM pH 7.4 phosphate-buffered saline (or 20 mM pH 7.4 Tris-HCl buffer in consistent to the buffer used in the modification). The Amicon® Ultra device was centrifuged under 4,000 RCF for 20 min by a BOECO CENTRIFUGE C-28A bench-top centrifuge. The purification process was repeated for three times. Modified proteins (0.1 mM) in 50 mM pH 7.4 phosphate-buffered saline (or 20 mM pH 7.4 Tris-HCl buffer in consistent to the buffer used in the modification) were collected.

○ unmodified ● mono-modified ◌di-modified

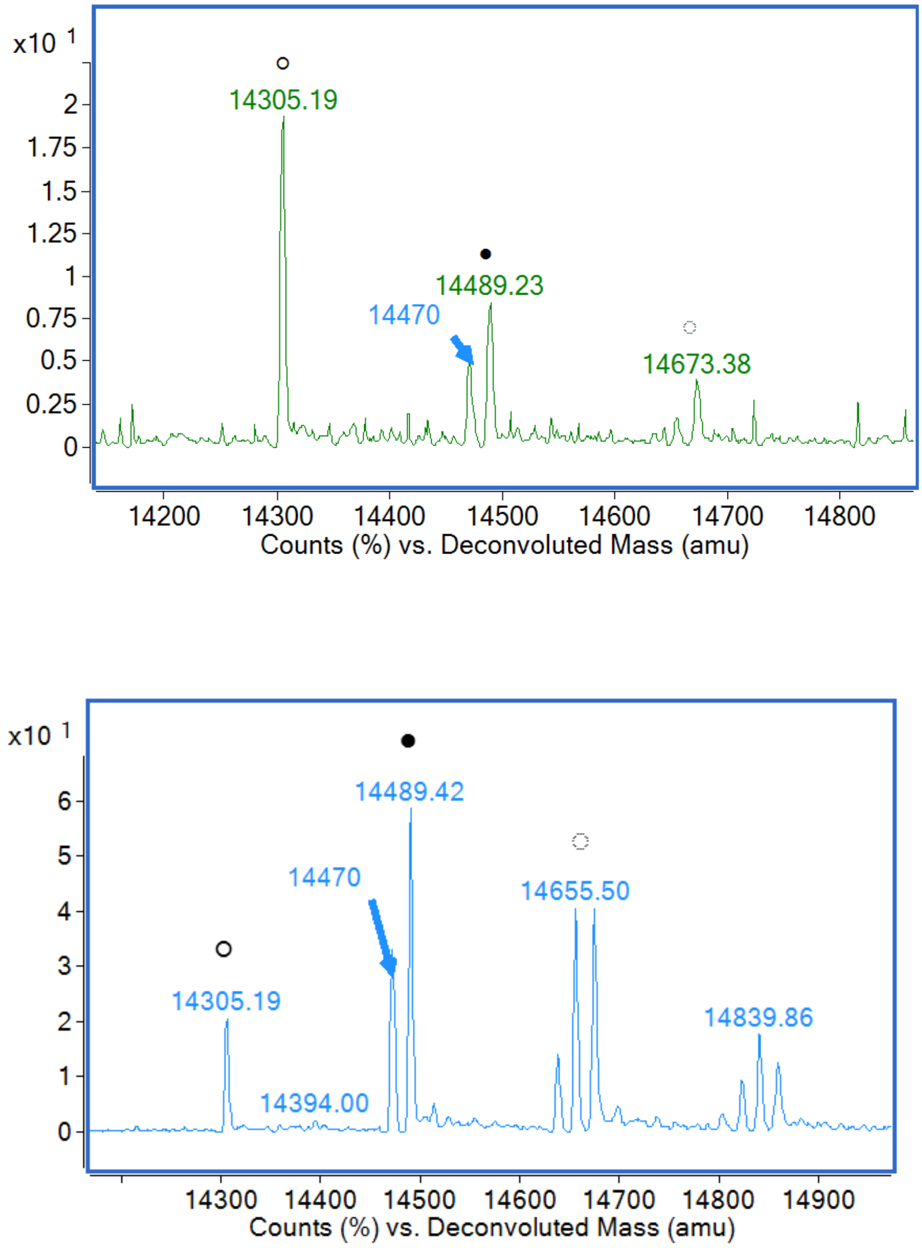

Supplementary Fig. 82 Deconvoluted mass spectrum in LC−MS analysis of the reaction mixtures in the modification of lysozyme using alkyne-linked 2-ethynylbenzaldehyde (**2t**) in 50 mM PBS at (a) pH 6.5 and (b) pH 7.4.

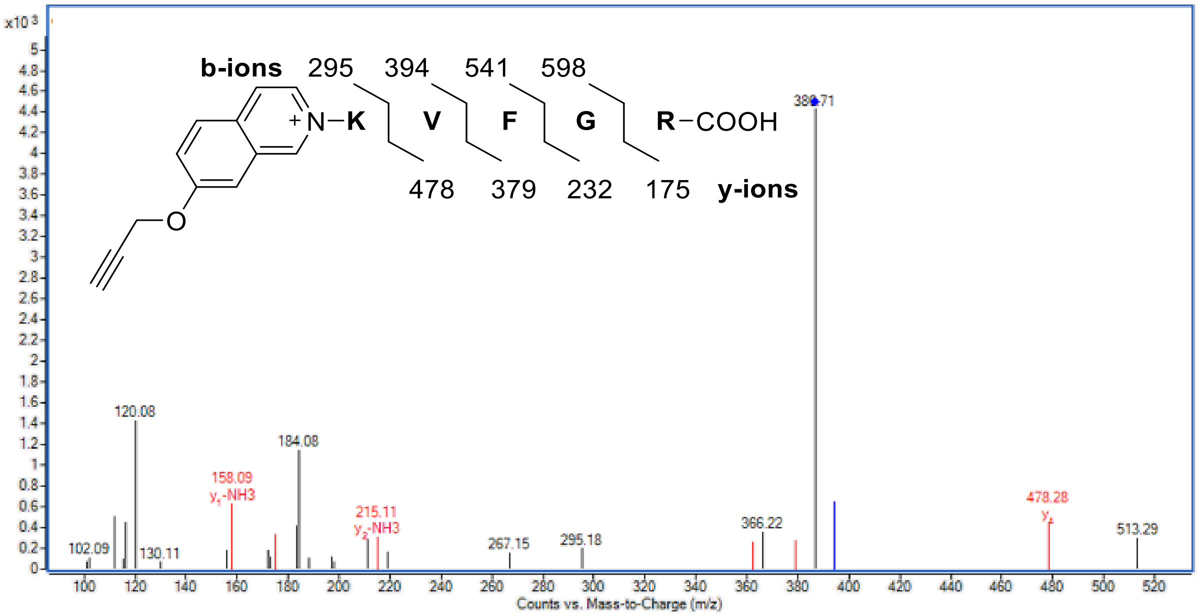

Supplementary Fig. 83 The MS/MS spectra of the N-terminal **2t**-linked fragment of lysozyme (the doubly charged ion of *m/z* = 386.71) in 50 mM PBS at pH 6.5.

○ unmodified ● mono-modified ◌di-modified

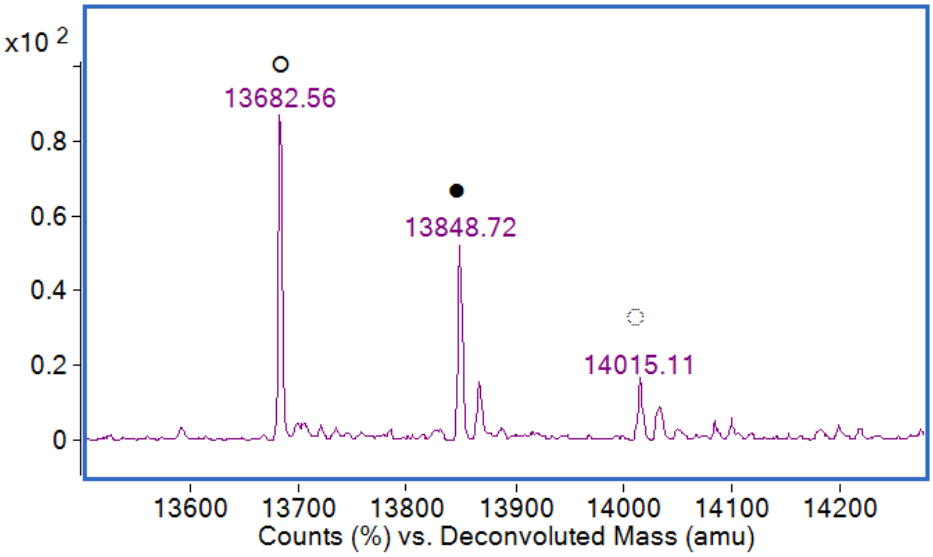

Supplementary Fig. 84 Deconvoluted mass spectrum in LC–MS analysis of the reaction mixtures in the modification of RNaseA using alkyne-linked 2-ethynylbenzaldehyde (**2t**) in 50 mM PBS at pH 7.4.

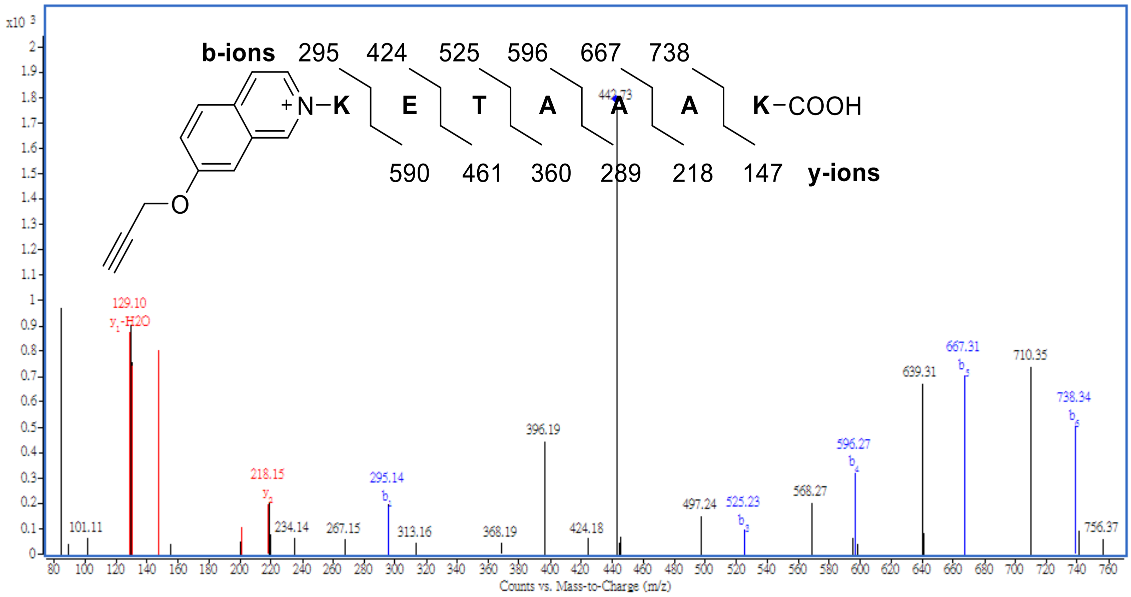

Supplementary Fig. 85 The MS/MS spectra of the N-terminal **2t**-linked fragment of RNase A (the doubly charged ion of m/z = 442.73) in 50 mM PBS at pH 7.4.

(a) ○ unmodified ● mono-modified ◌ di-modified

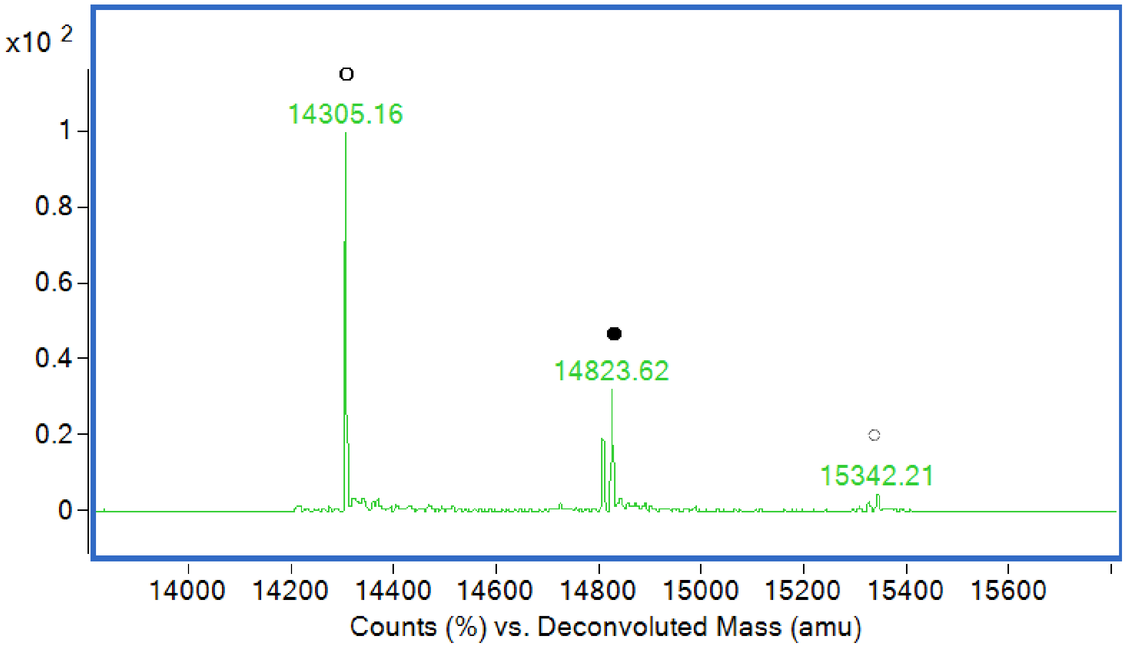

(b)

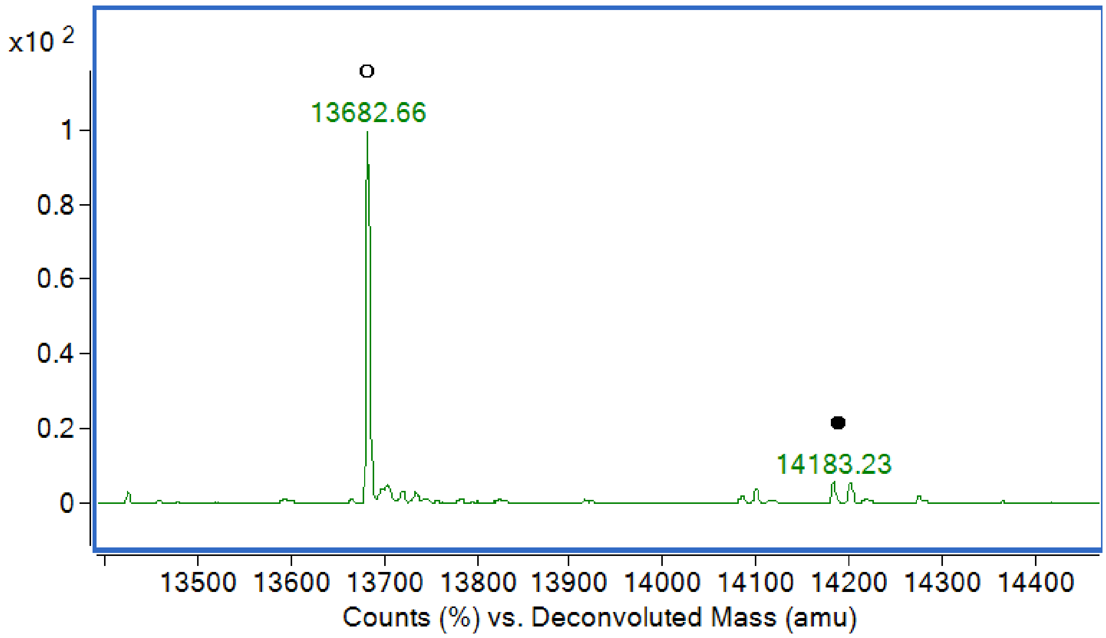

Supplementary Fig. 86 Deconvoluted mass spectrum in LC−MS analysis of the reaction mixtures in the modification of (a) lysozyme and (d) RNase A using fluorescein-linked 2-ethynylbenzaldehyde (**2u**) in 50 mM PBS at pH 7.4.

(a)

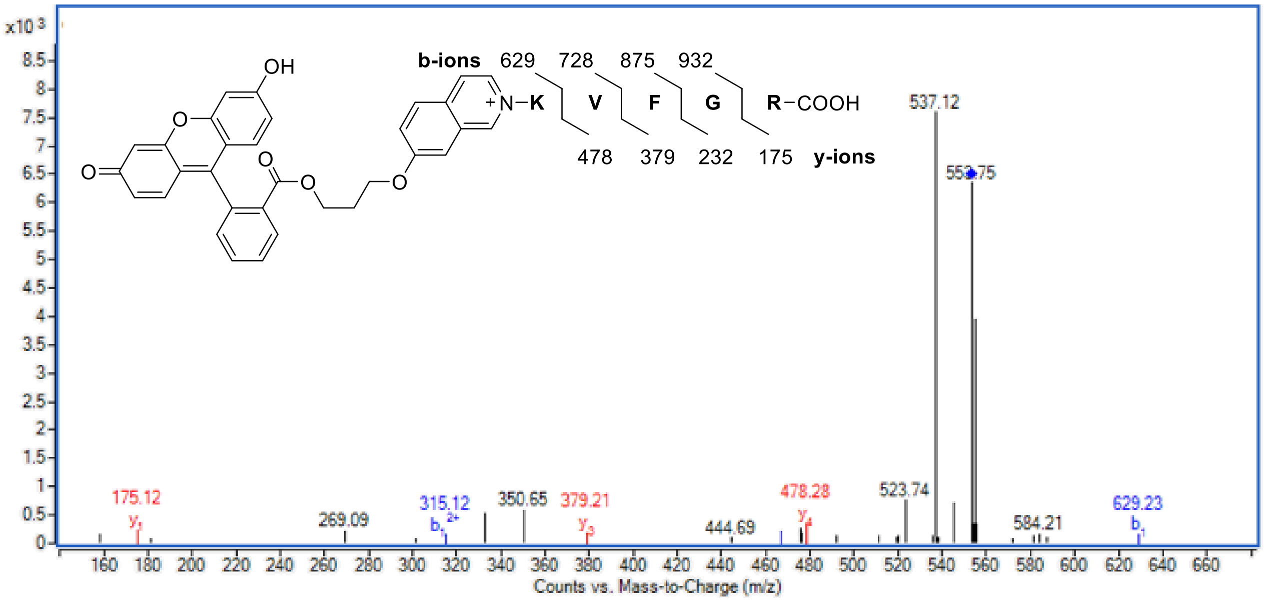

(b)

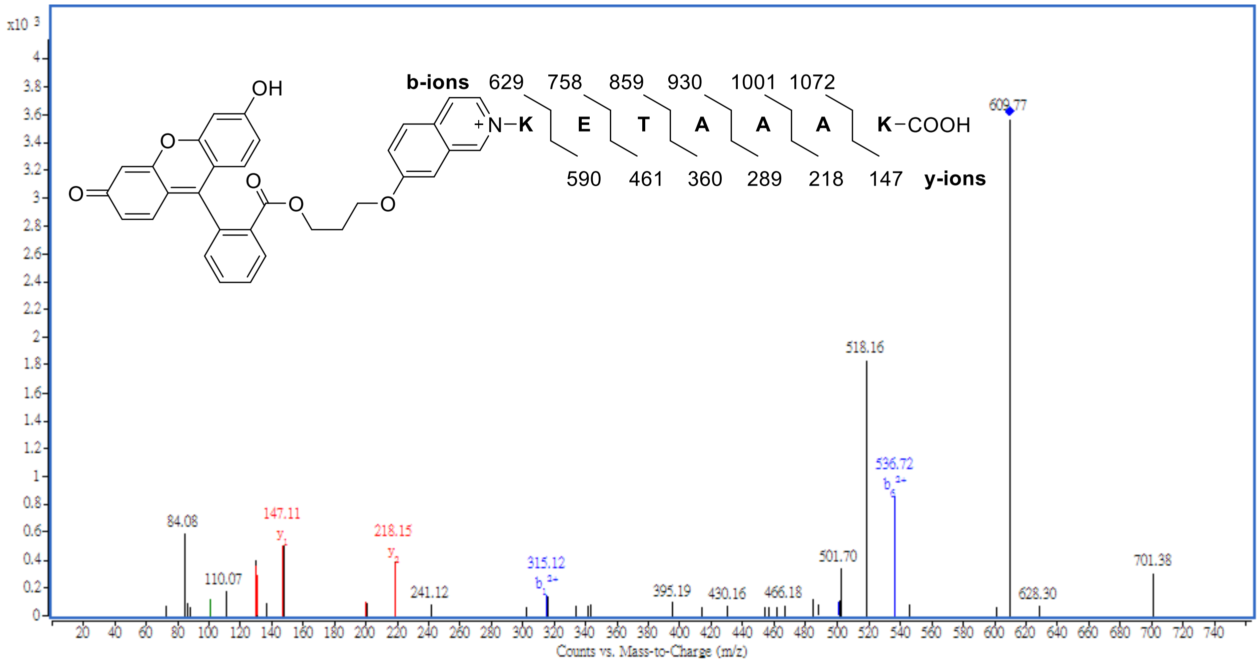

Supplementary Fig. 87 The MS/MS spectra of the N-terminal **2u**-linked fragment of (a) lysozyme (the doubly charged ion of *m/z* = 553.75) and (b) RNase A (the doubly charged ion of *m/z* = 609.77) in 50 mM PBS at pH 7.4.

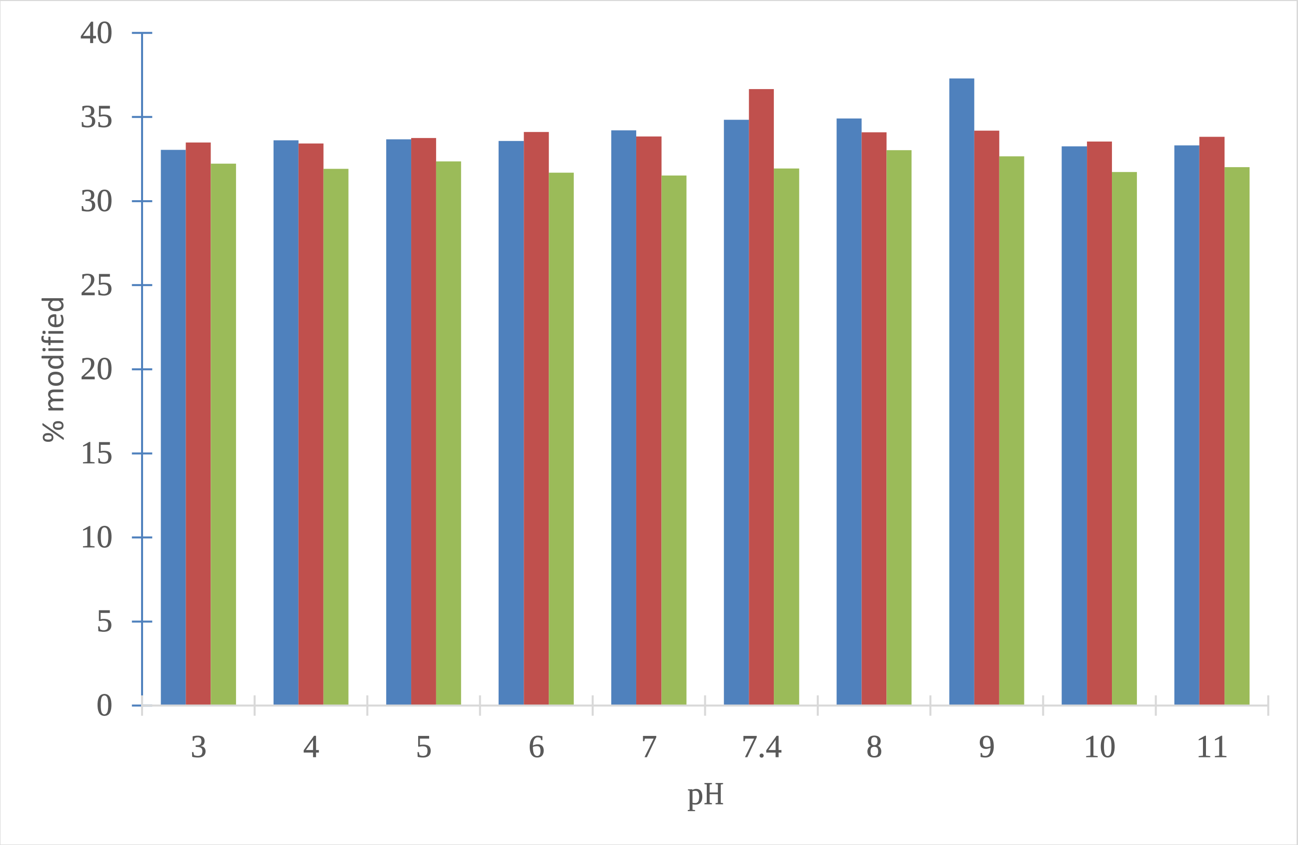

Supplementary Fig. 88 The stability of **2t**-modified RNaseA in different pH(s) of 50 mM phosphate-buffered saline at room temperature (blue bar), 37 ^{o}C for 1 h (red bar) and 37 ^{o}C for 12 h (green bar). The data shown are based on LC-MS analysis and represent a single experiment observation.

(a) ○ unmodified ● mono-modified ◌ di-modified

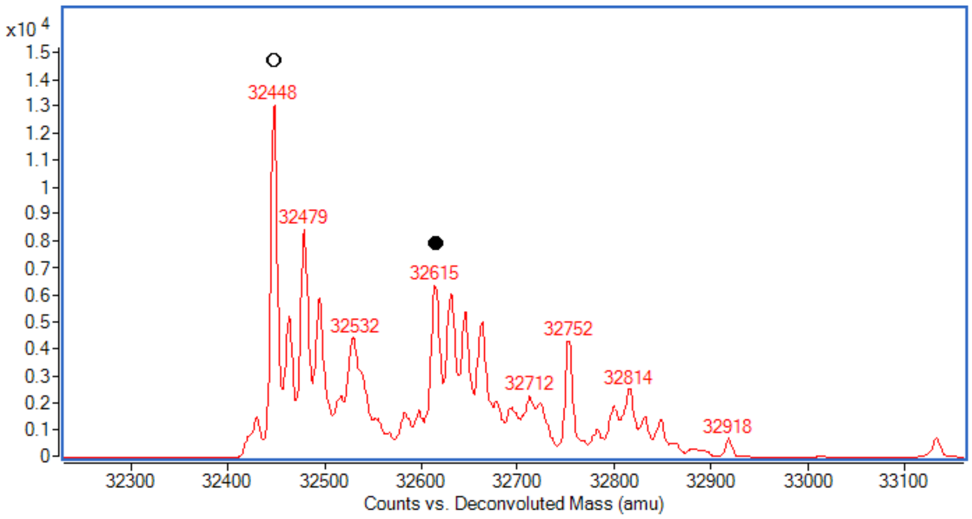

(b)

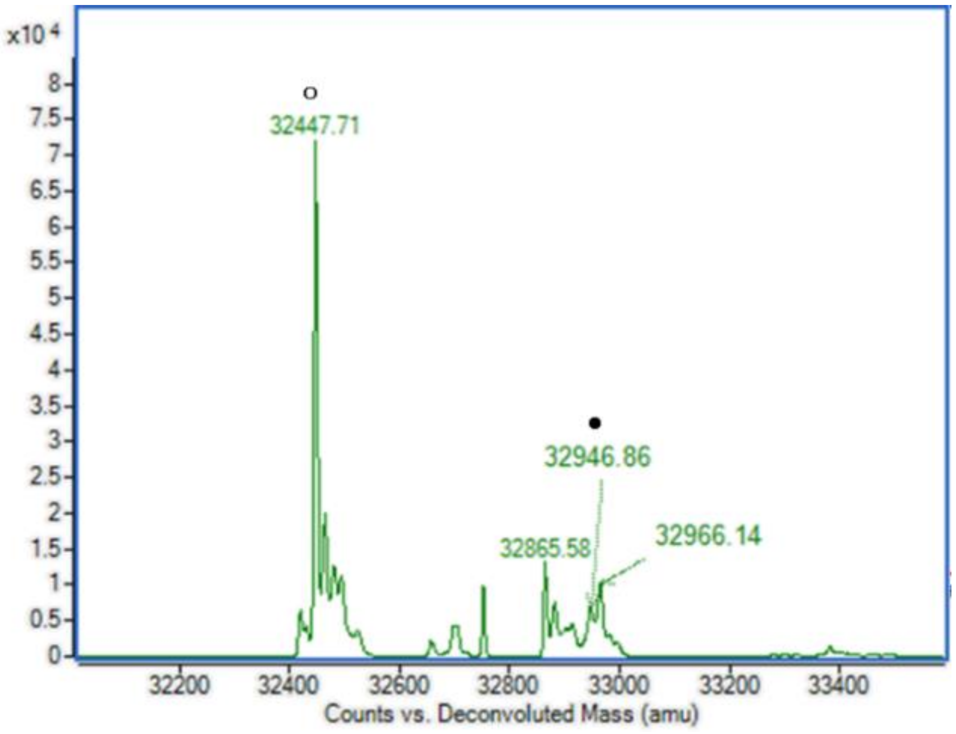

Supplementary Fig. 89 Deconvoluted mass spectrum in LC−MS analysis of the reaction mixtures in the modification of (a) BCArg mutant with **2t** and (B) BCArg mutant with **2u** in 50 mM PBS at pH 7.4.

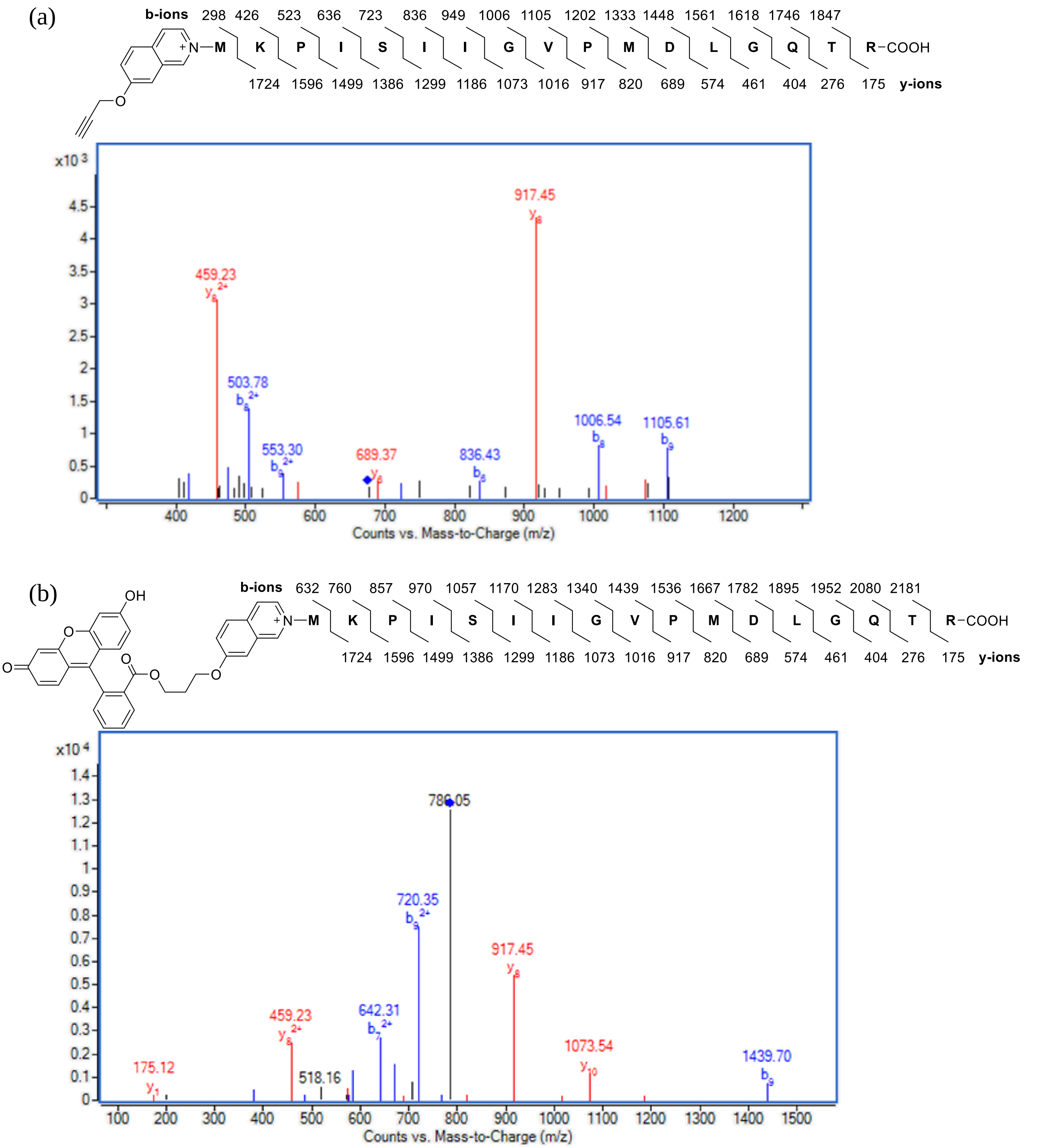

Supplementary Fig. 90 The MS/MS spectra of (a) the N-terminal **2t**-linked fragment of BCArg mutant (the triply charged ion of *m/z* = 674.67) and (b) N-terminal **2u**-linked fragment of BCArg mutant (the triply charged ion of *m/z* = 786.05) in 50 mM PBS at pH 7.4.

Sequential Modification of 2t-linked BCArg mutant *via* Copper(I)-catalyzed Azide-Alkyne Cycloaddition Reaction

A mixture of 50 μL of **2t**-modified BCArg mutant (0.1 mM in 50 mM pH 7.4 PBS buffer), 5 μL of rhodamine azide (5 mM in DMSO), 5 μL of TBTA (5 mM in DMSO), 5 μL of TCEP (5 mM in H_2O), 5 μL of $CuSO_4$ solution (5 mM in H_2O) and 30 μL of pH 7.4 PBS buffer was treated in a 1.5 mL Eppendorf tube at 25 °C for 1 h. Rhodamine-labelled BCArg mutant was characterized by LC-MS analysis.

○ unmodified ● **2r**-modified ◊ rhodamine-labelled

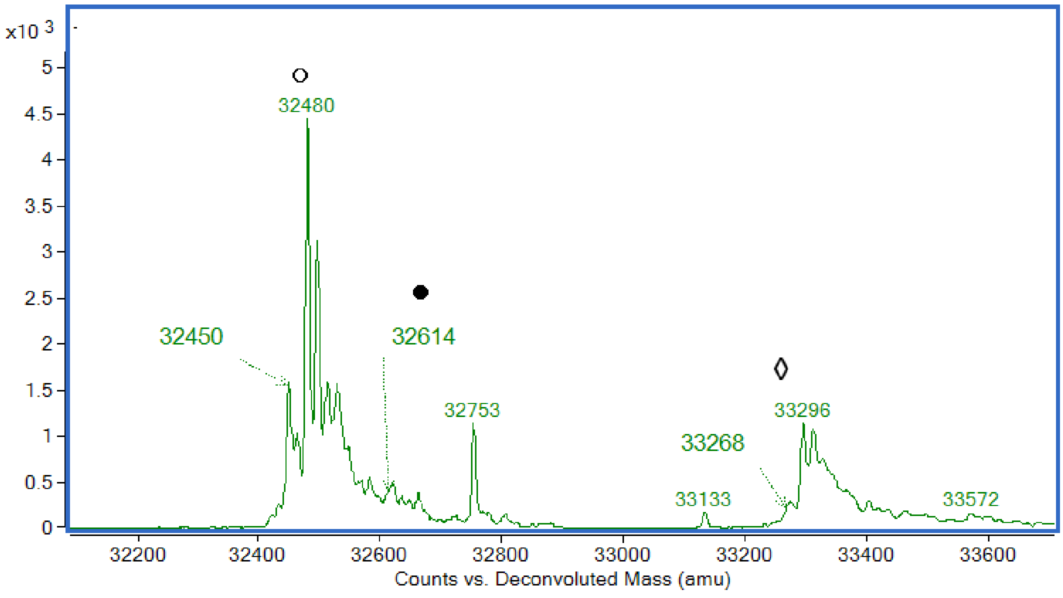

Supplementary Fig. 91 Deconvoluted mass spectrum in LC−MS analysis of the reaction mixture in the cycloaddition reaction of **2t**-modified BCArg mutant with rhodamine azide in 50 mM PBS at pH 7.4.

SDS-PAGE Analysis of Native and Modified Proteins

Scaning of fluorescent signal (with 526, and 628 nm band pass filters) and Coomassie blue staining of the gel was performed by the Azure™ Biosystems c600 imager.

	BCArg mutant	**2t**-modified BCArg mutant	rhodamine-labelled BCArg mutant
UV (365 nm)	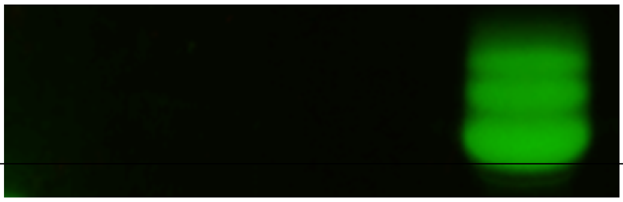		
Coomassie Blue	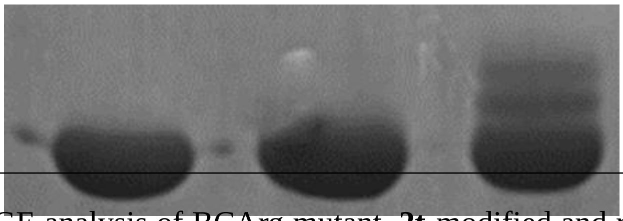		

Supplementary Fig. 92 SDS-PAGE analysis of BCArg mutant, **2t**-modified and rhodamine-labelled BCArg mutants.

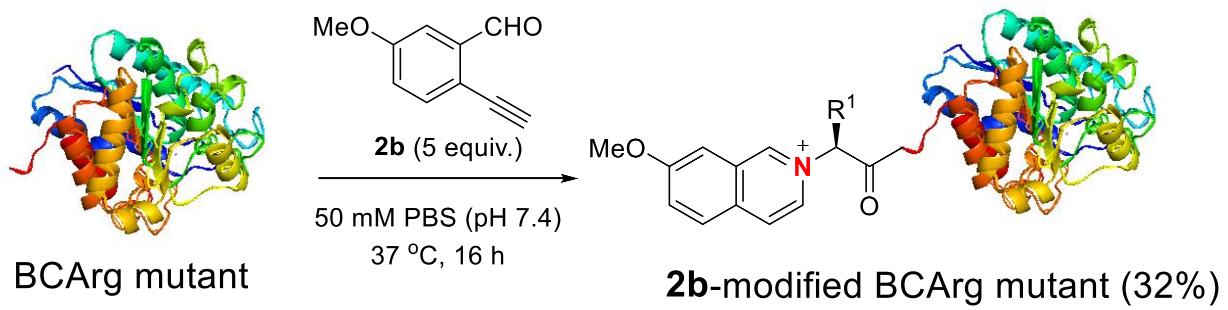

Supplementary Fig. 93 Modification of BCArg mutant using **2b**.

○ unmodified ● mono-modified

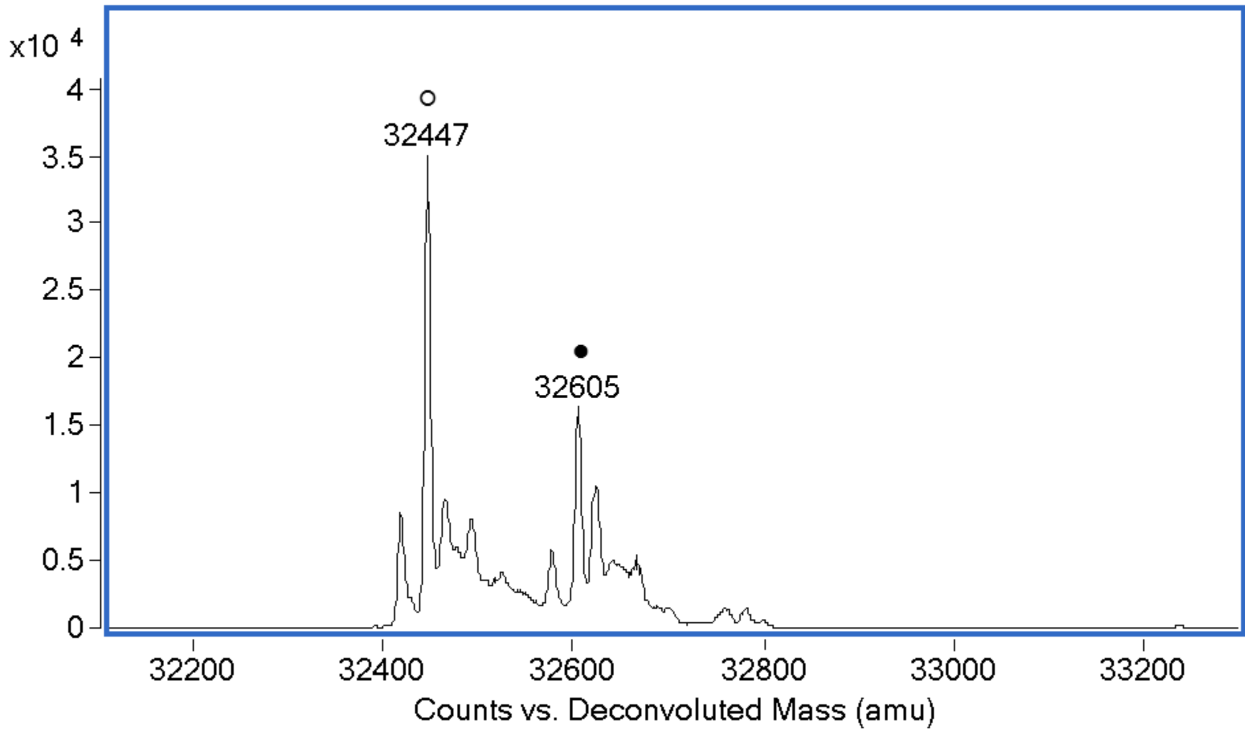

Supplementary Fig. 94 Deconvoluted mass spectrum in LC−MS analysis of the reaction mixtures in the modification of BCArg mutant with **2b** in 50 mM PBS at pH 7.4.

Procedure for Site-Directed Mutagenesis of BCArg mutant

A Cys161 was mutated on Ser161 of BCArg mutant following the procedures as shown below.

Cloning

The BCArg mutant plasmid was constructed following the procedures depicted in the following patent: Leung, Y. C.; Lo, W. Site-directed pegylation of arginases and the use thereof as anti-cancer and anti-viral agents, U.S. Patent 8507245B2, Aug 13, 2013.

Protein Expression and Purification

DNA plasmid pET3a/BCArg mutant was transformed into competent *E.coli* BL21 (DE3) and the cells were incubated in a Lysogeny broth (LB) plate containing 100 μg/ml ampicillin at 37 ^{o}C overnight. Glycerol stocks were prepared with 18% glycerol and stored at -80 ^{o}C. BCArg mutant proteins were expressed in pET3a plasmid containing strong T7 promoter by 0.06% lactose induction at 30 ^{o}C for 18 h. Cells were collected by centrifugation and the cell pellet was lysed by an ultrasonic homogenizer (QSonica sonicators) in resuspension buffer (1 mM $MnCl_2$, 20 mM Tris buffer, pH 7.4). The crude cell lysates were centrifuged at 16,000 rpm for 2 hours at 4 ^{o}C. The clarified supernatant was incubated with 10 mM $MnCl_2$ at 80 ^{o}C for 15 min and any white precipitates formed were removed by centrifugation. Proteins were buffer exchanged into binding buffer (20 mM Tris buffer, pH 7.0) and then applied to HiTrap Q HP (GE Healthcare) column. The target proteins bound on the column were eluted by 30% step elution buffer (1 M NaCl/20 mM Tris buffer, pH 7.0). Proteins were buffer exchanged to 20 mM Tris buffer, pH 7.0 and stored in 4 ^{o}C.

Enzyme Activity

The chromophore compound was detected at a wavelength A530 nm in the presence of diacetyl monoxime, thiosemicarbzide, urea and Fe^{3+} under high temperature.[2] This assay was used to determine the amount of urea produced by BCArg mutant per second. Stock L-Arg (102.4 mM) solution was prepared using 1 X GIBCO® PBS, pH 7.4 and diluted to 20 mM. L-Arg solutions (200 μL) were incubated at 37 ^{o}C using a heat block. Reactions were started by adding 5 μL of arginase (0.02 mg/ml) and stopped with 15 μL 80% trichloroacetic acid. The reaction time was 30 s. A coloring reagent was prepared by 1 volume of a mixture of 80 mM diacetyl monoxime and 2.0 mM thiosemicarbzide and 3 volumes of a mixture of 3 M H_3PO_4, 6 M H_2SO_4, 2 mM $FeCl_3$. 800 μL of the coloring reagent were added to each reaction, and then the reaction mixtures were incubated at 100 ^{o}C for 15 min followed by cooling down for 5 min at room temperature. A530 nm was determined using UV-Vis spectroscopy (Spectronic 20 Genesys Spectrometer). The specific activity of BCArg mutant enzyme is defined as the micromoles of urea formed per minute under given conditions per milligram proteins at 37 ^{o}C, pH 7.4 in phosphate buffered saline buffer (expressed in μmol $min^{-1}mg^{-1}$).

BCArg mutant Peptide Sequence

MKPISIIGVP MDLGQTRRGP DMGPSAMRYA GVIERLERLH YDIEDLGDIP

IGKAERLHEQ GDSRLRNLKA VAEANEKLAA AVDQVVQRGR FPLVLGGDHS

IAIGTLAGVA KHYERLGVIW YDAHGDVNTA ETSPSGNIHG MPLAASLGFG HPALTQIGGY

CPKIKPEHVV LIGVRSLDEG EKKFIREKGI KIYTMHEVDR LGMTRVMEET IAYLKERTDG

VHLSLDLDGL DPSDAPGVGT PVIGGLTYRE SHLAMEMLAE AQIITSAEFV EVNPILDERN

KTASVAVALM GSLFGEKLM

Cell Culture

Two human cancer cell lines (breast cancer cell lines MDA-MB-231 and MDA-MB-468) were purchased from ATCC. They were cultured in medium according to ATCC guidelines. Culture media (DMEM, RPMI-1640 and MEM), FBS and P/S were purchased from Thermo Fisher Scientific.

Cell Proliferation Assay

Two human cancer cell lines (breast cancer cell line MDA-MB-231 and MDA-MB-468) were seeded in 96-well plate at densities of 5 x 10^3 cells / well. After 1 d of incubation, the culture medium was replaced with medium containing different concentrations of BCArg mutant (20, 4, 0.8, 0.16, 0.032 U/ml). MTT assay was conducted at Day 3 (3 d after BCArg mutant treatment) to determine the IC_{50} at day 3. The culture medium was replaced with MTT solution (1 mg/ml) (Invitrogen) and incubated at 37 ^{o}C for 4 h. After 4 h of incubation, MTT solution was replaced with DMSO, and the absorbance at 570 nm was measured with a reference of 650 nm. The cell viability was determined by dividing the absorbance of BCArg mutant-treated cells by the average absorbance of untreated cells. The IC_{50} of different BCArg mutants on different cancer cell lines (at 95% confidence interval) were analyzed by software Prism 6.0. Three independent sets of experiments (n = 3) were performed for each cell line.

Supplementary References:

1. Sonogashira, K.; Tohda, Y.; Hagihara, N. A Convenient Synthesis of Acetylenes: Catalytic Substitutions of Acetylenic Hydrogen with Bromoalkenes, Iodoarenes and Bromopyridines. *Tetrahedron Lett.* **1975**, *16*, 4467.
2. Knipp, M.; Vašák, M., A Colorimetric 96-Well Microtiter Plate Assay for the Determination of Enzymatically Formed Citrulline. *Anal. Biochem.* **2000**, *286*, 257.
