# Supplementary material for: N-Terminal selective modification of peptides and proteins using 2-ethynylbenzaldehydes
Source: Commun Chem. 2020 May 29;3:67. doi: 10.1038/s42004-020-0309-y (PMC9814395; doi:10.1038/s42004-020-0309-y)
Supplement: Supplementary file 2 — Reporting Summary [file 42004_2020_309_MOESM2_ESM.pdf]

nature research

Corresponding author(s): Karen Ka-Yan Kung, Yun-Chung Leung and Man-Kin Wong

Last updated by author(s): Apr 7, 2020

Reporting Summary

Nature Research wishes to improve the reproducibility of the work that we publish. This form provides structure for consistency and transparency in reporting. For further information on Nature Research policies, see Authors & Referees and the Editorial Policy Checklist .

Statistics

For all statistical analyses, confirm that the following items are present in the figure legend, table legend, main text, or Methods section.

n/a	Confirmed	
☐	☒	The exact sample size (*n*) for each experimental group/condition, given as a discrete number and unit of measurement
☐	☒	A statement on whether measurements were taken from distinct samples or whether the same sample was measured repeatedly
☐	☒	The statistical test(s) used AND whether they are one- or two-sided<br>*Only common tests should be described solely by name; describe more complex techniques in the Methods section.*
☒	☐	A description of all covariates tested
☒	☐	A description of any assumptions or corrections, such as tests of normality and adjustment for multiple comparisons
☐	☒	A full description of the statistical parameters including central tendency (e.g. means) or other basic estimates (e.g. regression coefficient) AND variation (e.g. standard deviation) or associated estimates of uncertainty (e.g. confidence intervals)
☒	☐	For null hypothesis testing, the test statistic (e.g. *F*, *t*, *r*) with confidence intervals, effect sizes, degrees of freedom and *P* value noted<br>*Give P values as exact values whenever suitable.*
☒	☐	For Bayesian analysis, information on the choice of priors and Markov chain Monte Carlo settings
☒	☐	For hierarchical and complex designs, identification of the appropriate level for tests and full reporting of outcomes
☒	☐	Estimates of effect sizes (e.g. Cohen's *d*, Pearson's *r*), indicating how they were calculated

Our web collection on statistics for biologists contains articles on many of the points above.

Software and code

Policy information about availability of computer code

Data collection	The data collected were basically mass spectrometric measurements , which did need software (MassHunter) to be collected.
Data analysis	Statistical analyses were performed using Excel from Windows 10 version and Prism 6.0. SDS-PAGE analysis was performed using Azure™ Biosystems c600 imager. Enzyme activity was performed using Spectronic 20 Genesys Spectrometer.

For manuscripts utilizing custom algorithms or software that are central to the research but not yet described in published literature, software must be made available to editors/reviewers. We strongly encourage code deposition in a community repository (e.g. GitHub). See the Nature Research guidelines for submitting code & software for further information.

Data

Policy information about availability of data

All manuscripts must include a data availability statement. This statement should provide the following information, where applicable:

- Accession codes, unique identifiers, or web links for publicly available datasets
- A list of figures that have associated raw data
- A description of any restrictions on data availability

Supplementary Information.

Field-specific reporting

Please select the one below that is the best fit for your research. If you are not sure, read the appropriate sections before making your selection.

☒ Life sciences ☐ Behavioural & social sciences ☐ Ecological, evolutionary & environmental sciences

For a reference copy of the document with all sections, see nature.com/documents/nr-reporting-summary-flat.pdf

Life sciences study design

All studies must disclose on these points even when the disclosure is negative.

Sample size	Please see Supplementary Information.
Data exclusions	No data were excluded in all measurements.
Replication	As usual in enzymatic activity and cell proliferation assays.
Randomization	Cancer cells were uniformly seeded in 96-well plate
Blinding	Blinding was not relevant to our study.<br>It is because no clinical study was conducted in this manuscript.

Reporting for specific materials, systems and methods

We require information from authors about some types of materials, experimental systems and methods used in many studies. Here, indicate whether each material, system or method listed is relevant to your study. If you are not sure if a list item applies to your research, read the appropriate section before selecting a response.

Materials & experimental systems

n/a	Involved in the study
☒	☐ Antibodies
☐	☒ Eukaryotic cell lines
☒	☐ Palaeontology
☒	☐ Animals and other organisms
☒	☐ Human research participants
☒	☐ Clinical data

Methods

n/a	Involved in the study
☒	☐ ChIP-seq
☒	☐ Flow cytometry
☒	☐ MRI-based neuroimaging

Eukaryotic cell lines

Policy information about cell lines

Cell line source(s)	MDA-MB-468 and MDA-MB-231 were obtained from American Type Culture Collection
Authentication	All cell lines were authenticated as 100% match by STR DNA analysis with PowerPlex® 16HS kit.
Mycoplasma contamination	No mycoplasma was detected in all cell lines using MycoFluor™ Mycoplasma Detection Kit (Thermofisher).
Commonly misidentified lines<br>(See ICLAC register)	No commonly misidentified cell line were used in this study.
